# *CCNE1* amplification is synthetic lethal with PKMYT1 kinase inhibition

**DOI:** 10.1038/s41586-022-04638-9

**Published:** 2022-04-20

**Authors:** David Gallo, Jordan T. F. Young, Jimmy Fourtounis, Giovanni Martino, Alejandro Álvarez-Quilón, Cynthia Bernier, Nicole M. Duffy, Robert Papp, Anne Roulston, Rino Stocco, Janek Szychowski, Artur Veloso, Hunain Alam, Prasamit S. Baruah, Alexanne Bonneau Fortin, Julian Bowlan, Natasha Chaudhary, Jessica Desjardins, Evelyne Dietrich, Sara Fournier, Chloe Fugère-Desjardins, Theo Goullet de Rugy, Marie-Eve Leclaire, Bingcan Liu, Vivek Bhaskaran, Yael Mamane, Henrique Melo, Olivier Nicolas, Akul Singhania, Rachel K. Szilard, Ján Tkáč, Shou Yun Yin, Stephen J. Morris, Michael Zinda, C. Gary Marshall, Daniel Durocher

**Affiliations:** 1 Lunenfeld-Tanenbaum Research Institute, Mount Sinai Hospital, Toronto, Ontario Canada; 2 Repare Therapeutics, Saint-Laurent, Quebec Canada; 3 Repare Therapeutics, Cambridge, MA USA; 4 Department of Molecular Genetics, University of Toronto, Toronto, Ontario Canada

**Keywords:** Oncogenes, Cell division, Target identification, Functional genomics, Genomic instability

## Abstract

Amplification of the *CCNE1* locus on chromosome 19q12 is prevalent in multiple tumour types, particularly in high-grade serous ovarian cancer, uterine tumours and gastro-oesophageal cancers, where high cyclin E levels are associated with genome instability, whole-genome doubling and resistance to cytotoxic and targeted therapies^1–4^. To uncover therapeutic targets for tumours with *CCNE1* amplification, we undertook genome-scale CRISPR–Cas9-based synthetic lethality screens in cellular models of *CCNE1* amplification. Here we report that increasing *CCNE1* dosage engenders a vulnerability to the inhibition of the PKMYT1 kinase, a negative regulator of CDK1. To inhibit PKMYT1, we developed RP-6306, an orally bioavailable and selective inhibitor that shows single-agent activity and durable tumour regressions when combined with gemcitabine in models of *CCNE1* amplification. RP-6306 treatment causes unscheduled activation of CDK1 selectively in *CCNE1-*overexpressing cells, promoting early mitosis in cells undergoing DNA synthesis. *CCNE1* overexpression disrupts CDK1 homeostasis at least in part through an early activation of the MMB–FOXM1 mitotic transcriptional program. We conclude that PKMYT1 inhibition is a promising therapeutic strategy for *CCNE1*-amplified cancers.

## Abstract (web summary)

Genome-scale CRISPR–Cas9-based synthetic lethality screens identify *PKMYT1* as a potential therapeutic target in tumours with CCNE1 amplification.

## Main

In ovarian cancer, *CCNE1* amplification is detected in about 20% of tumours, in a manner largely mutually exclusive with homologous recombination deficiency, and is enriched in platinum-refractory tumours^2,5^. The paucity of therapeutic options for *CCNE1*-amplified tumours makes the development of novel therapeutic agents that target this amplification a critical unmet need^6^. Cyclin E itself is not considered to be a druggable target but its cognate cyclin-dependent kinase (CDK) CDK2 is. CDK2 inhibition shows promising activity in *CCNE1*-amplified cell lines^7^ and selective CDK2 inhibitors are starting to enter clinical development. As an alternative approach, we surmised that a synthetic-lethality approach^8^ exploiting vulnerabilities caused by the increase in cyclin E levels may provide much-needed novel therapeutic options for *CCNE1*-amplified tumours.

## PKMYT1 is essential in *CCNE1*-high cells

**Fig. 1:**
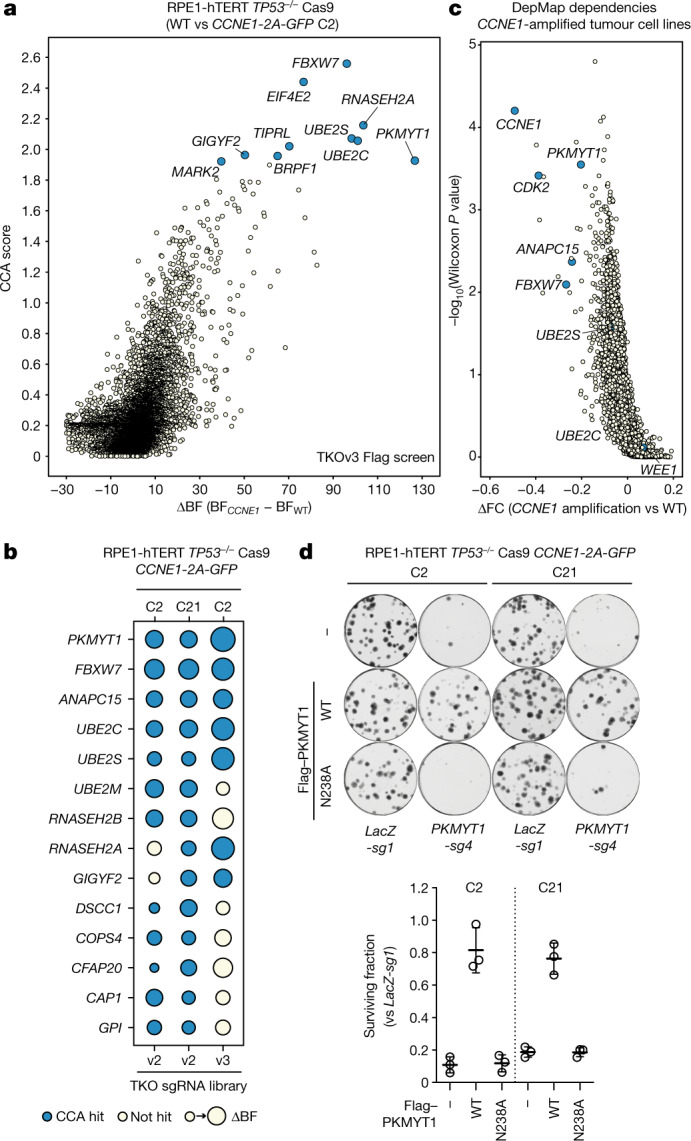
PKMYT1 is synthetic lethal in combination with *CCNE1* overexpression. **a**, Results of a CRISPR-based synthetic lethal screen in RPE1-hTERT *TP53*^*−/−*^ Cas9 *CCNE1-2A-GFP* (C2) cells with CCA and ΔBF scores plotted. **b**, Dot plot of the synthetic lethal hits from three screens. The size of the dots is proportional to the ΔBF score and blue indicates a hit using CCA (Jenks ranks > 2). **c**, Volcano plot of gene dependencies in cancer cell lines from the DepMap project grouped according to their *CCNE1* amplification status. **d**, Clonogenic survival assays of the indicated RPE1-hTERT *TP53*^*−/−*^ Cas9 cell lines transduced with lentivirus expressing sgRNA targeting *LacZ* (*LacZ-sg1*) or *PKMYT1* (*PKMYT1-sg4*) or doxycycline-inducible sgRNA-resistant Flag alone (−), *Flag–PKMYT1* or *Flag-PKMYT1*^*N238A*^. Top, representative images of plates stained with crystal violet. Bottom, quantification of clonogenic survival assays. Data are mean ± s.d. (*n* = 3). Source data

**Extended Data Fig. 1:**
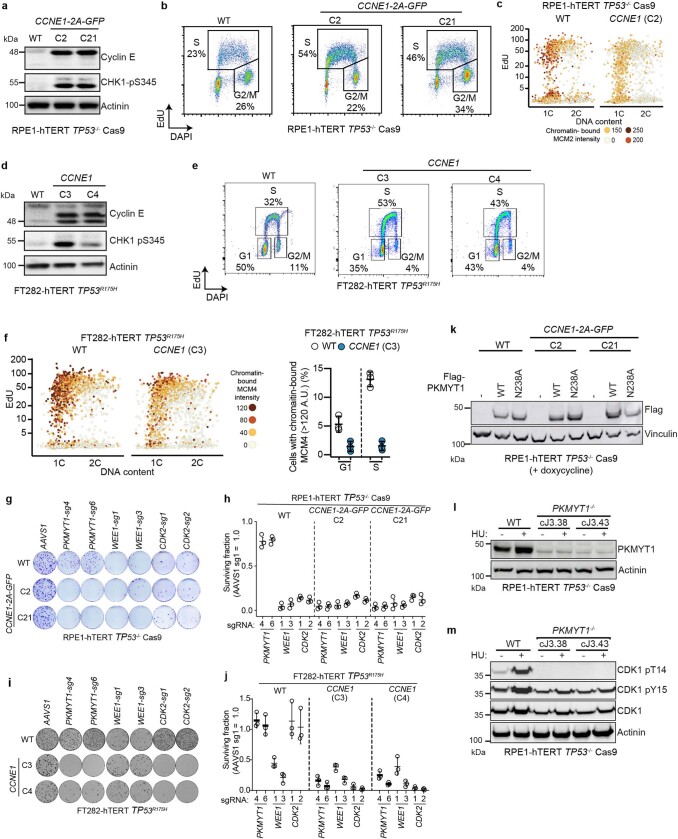
Characterization of *CCNE1*-high isogenic cell lines and validation of PKMYT1 synthetic lethality. **a,d**, Whole cell lysates of RPE1-hTERT* TP53*^-/-^ Cas9 *CCNE1-2A-GFP* (**a**) and FT282-hTERT *TP53*^*R175H*^ (**d**) *CCNE1*-high and parental (WT) cells were immunoblotted with cyclin E, CHK1-pS345 and actinin specific antibodies. **b**, FACS analysis of RPE1-hTERT *TP53*^*-/-*^ Cas9 *CCNE1*-high and parental (WT) cells for EdU incorporation and DNA content (DAPI). Percentage of each population gated for EdU^+^ (S) and EdU^-^ 2C (G2/M) are indicated. **c**. QIBC analysis of RPE1-hTERT *TP53*^*-/-*^ Cas9 *CCNE1*-high and parental cells for chromatin-bound MCM2 nuclear intensity, EdU incorporation and DNA content (DAPI). **e**, FACS analysis FT282-hTERT *TP53*^*R175H*^ *CCNE1*-high and parental (WT) cells for EdU incorporation and DNA content (DAPI). Percentage of each population gated for EdU^-^ 1C (G1), EdU^+^ (S) and EdU^-^ 2C (G2/M) are indicated. **f**, Left, QIBC analysis of FT282-hTERT *TP53*^*R175H*^ *CCNE1*-high and parental cells for chromatin-bound MCM4 nuclear intensity, EdU incorporation and DNA content (DAPI). Right, quantitation of EdU^-^ 1C (G1) and EdU^+^ (S) nuclei with chromatin-bound MCM4 (A.U. > 120). Data are shown as mean ± s.d. (n = 3). **g–j**, Clonogenic survival assays of (**g**,**h**) the indicated RPE1-hTERT*TP53*^*-/-*^ Cas9 cell lines transduced with lentivirus expressing the indicated sgRNAs and (**i**,**j**) the indicated FT282-hTERT *TP53*^*R175H*^ cell lines nucleofected with Cas9 ribonucleoproteins assembled with the indicated sgRNAs. Shown in (g,i) are representative plates with colonies stained with crystal violet. Quantitation is shown in (**h**, **j**). Data are shown as mean ± s.d. (n = 3). **k**, Whole cell lysates of RPE1-hTERT *TP53*^*-/-*^ Cas9 *CCNE1*-high and parental cells expressing doxycycline-inducible sgRNA-resistant Flag alone (-), *Flag-PKMYT1* or *Flag-PKMYT1*^*N238A*^ were immunoblotted with Flag and actinin antibodies. **l, m**, Whole cell lysates of RPE1-hTERT *TP53*^−/−^ Cas9 parental and two independent *PKMYT1*^*-/-*^ clones either untreated or treated for 24 h with 3 mM hydroxyurea (HU) were immunoblotted for PKMYT1 (**l**) and either total CDK1, CDK1-pT14 or CDK-pY15 (**m**). Actinin was used as loading control. For gel source data, see Supplementary Fig. 1. Source data

To identify genetic vulnerabilities to increased *CCNE1* dosage, we developed an isogenic pair of cell lines that stably overexpress cyclin E from a *CCNE1-2A-GFP* fusion integrated into the genome of RPE1-hTERT *TP53*^*−/−*^ Cas9 cells^9^, hereafter referred to as ‘*CCNE1-*high’ (Extended Data Fig. 1a). We characterized two clones, C2 and C21, which showed accumulation of cells in early S phase, elevated DNA replication stress and MCM helicase loading defects (Extended Data Fig. 1a–c). We performed genome-scale CRISPR–Cas9 screens in the parental and both *CCNE1*-high clones using the TKOv2 single guide RNA (sgRNA) library^10^ and subsequently rescreened clone C2 with the TKOv3 sgRNA library, which has improved performance^11^ (Fig. 1a). Using two CRISPR screen scoring methods, CCA^12^ and BAGEL2^13^, we identified five genes whose mutation caused a selective loss of fitness in *CCNE1*-high cells, in all three screens: *ANAPC15*, *FBXW7*, *PKMYT1*, *UBE2C* and *UBE2S* (Fig. 1b, Supplementary Table 1). To prioritize this list, we mined data from the cancer dependency (DepMap) project^14^. This analysis identified *PKMYT1* as the gene that displayed the strongest dependency in *CCNE1*-amplified tumour cell lines (Fig. 1c). *PKMYT1* encodes an evolutionarily conserved protein kinase, also known as Myt1, whose primary role is the negative regulation of CDK1 both by its inhibitory phosphorylation on Thr14 and its sequestration in the cytoplasm^15–19^. PKMYT1 is structurally related to—and much less studied than—WEE1, which phosphorylates the adjacent Tyr15 residue on CDK1 and CDK2 to inhibit these kinases^20–22^. Unlike WEE1, which is nuclear-localized, PKMYT1 is cytoplasmic owing to an interaction with endomembranes of the Golgi and the endoplasmic reticulum^16,23^. *WEE1* did not score as a hit in either of our isogenic synthetic lethal screens or in our analysis of the DepMap data (Fig. 1c) indicating that *CCNE1*-amplified cells may have a unique vulnerability to the loss of PKMYT1.

The identification of a protein kinase, part of a highly druggable enzyme class, prompted us to validate the synthetic lethality between the loss of *PKMYT1* and elevated *CCNE1* levels in the original RPE1 cell line background and in an isogenic set of immortalized FT282-hTERT fallopian tube cell lines expressing *TP53*^*R175H*^ with or without *CCNE1* overexpression^24^. The FT282 *CCNE1-*high cells show accumulation in early S phase, evidence of replication stress, and MCM loading defects^24^ (Extended Data Fig. 1d–f). Two sgRNAs targeting *PKMYT1* caused selective lethality in the *CCNE1*-high cells of both backgrounds, while sparing their parental counterparts (Extended Data Fig. 1g–j, Supplementary Table 2 for tracking of indels by decomposition (TIDE) analysis). Reintroduction of an sgRNA-resistant *PKMYT1* transgene protected RPE1 *CCNE1*-high cells from depletion of endogenous *PKMYT1*, whereas expression of *PKMYT1*^*N238A*^, which encodes a kinase-dead protein, did not (Fig. 1d, Extended Data Fig. 1k). We conclude that the protein kinase activity of PKMYT1 is essential in cells that are engineered to overexpress cyclin E.

Introduction of sgRNAs targeting *WEE1* was lethal in the RPE1- and FT282-derived cells irrespective of their *CCNE1* status, whereas sgRNAs targeting *CDK2* were lethal in all RPE1 cell lines and selectively lethal in FT282 *CCNE1*-high cells (Fig. 1g–j, Supplementary Table 2). We could generate and propagate clonal knockouts of *PKMYT1* in *TP53*^*−/−*^ RPE1-hTERT cells that displayed complete loss of CDK1 Thr14 phosphorylation without grossly affecting CDK1 Tyr15 phosphorylation, indicating that *PKMYT1* can be dispensable for normal cell viability (Extended Data Fig. 1l, m). As loss of WEE1 affects both CDK1 and CDK2^22^, and as cyclin E activates CDK2, these observations suggested a simple model in which activation of CDK1 is incompatible with viability of cells overexpressing *CCNE1*.

## Characterization of RP-6306

**Fig. 2:**
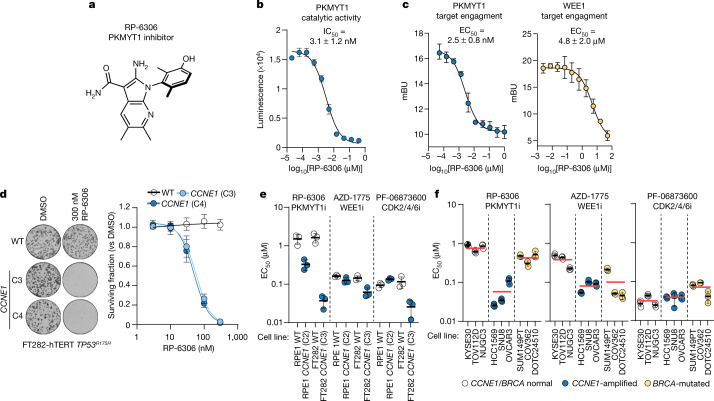
RP-6306 is a selective PKMYT1 inhibitor with activity in CCNE1-amplified cells. **a**, Chemical structure of the PKMYT1 inhibitor RP-6306. **b**, Dose–response of PKMYT1 catalytic activity to RP-6306 as measured with the ADP-Glo kinase assay. Data are mean ± s.d. (*n* = 3). **c**, Target engagement of RP-6306 on PKMYT1 (left) and WEE1 (right) in a NanoBRET assay reported in milliBRET units (mBU). Data are mean ± s.d. (*n* = 3). **d**, Clonogenic survival of the indicated FT282-hTERT *TP53*^*R175H*^ derivatives treated with RP-6306. Left, representative images of plates stained with crystal violet. Right, quantification of clonogenic survival assays. Data are mean ± s.d. (*n* = 3). **e**, **f**, EC_50_ determination for growth inhibition for the parental and *CCNE1*-high cells in the RPE1-hTERT *TP53*^*−/−*^ Cas9 (RPE1) and FT282-hTERT *TP53*^*R175H*^ (FT282) backgrounds (**e**) and indicated cancer cell lines (**f**) treated with the indicated compounds. Growth was monitored with an Incucyte live-cell imager for up to six population doublings. Data are mean ± s.d. (*n* = 3). Additional data are presented in Extended Data Fig. 2g, h. In **f**, cell lines are also grouped according to their *CCNE1* or *BRCA* status and the red bar indicates the mean of each grouping. Source data

**Extended Data Fig. 2:**
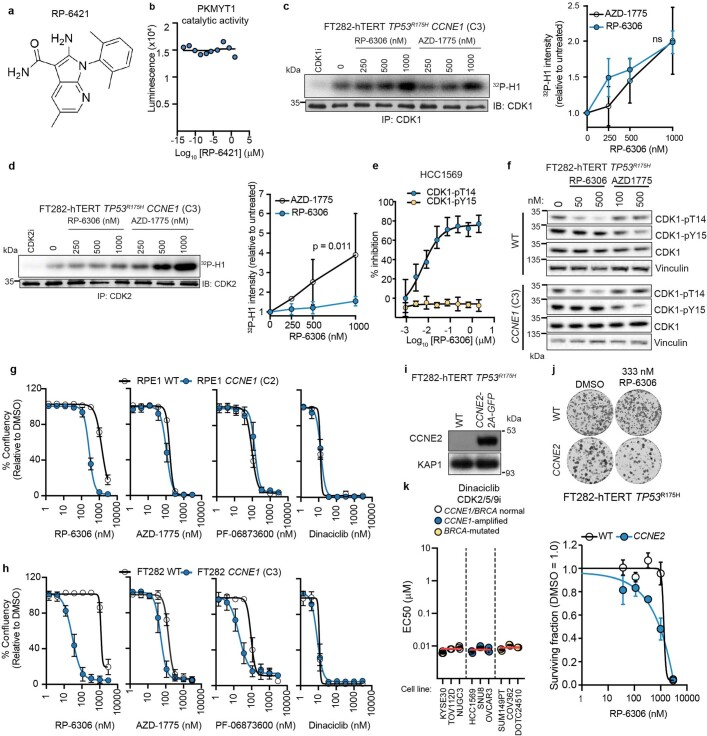
Development of RP-6306 and comparison of PKMYT1i, WEEi and CDK2i. **a**, Chemical structure of RP-6421, an analog of RP-6306 **b**, Dose-response of RP-6421 on PKMYT1 catalytic activity as measured with ADP-Glo kinase assay. **c, d**, Cell culture of FT282-hTERT*TP53*^*R175H*^ *CCNE1*-high cells were treated with the indicated doses of RP-6306 and AZD-1775 for 24 h. Left, cellular extracts were prepared and immunoprecipitated (IP) with agarose-coupled CDK1 (**c**) or CDK2 (**d**) antibodies. Immunoprecipitates were subjected to in vitro kinase assays using [γ^32^P]-ATP and recombinant histone H1 as a substrate. Reactions were resolved by SDS-PAGE and gels were imaged using a phosphor screen. A sample of each immunoprecipitate was immunoblotted (IB) with CDK1 or CDK2 antibodies. CDK1i (RO-3306) or CDK2i (PF-06873600) was added to indicated in vitro reactions. Right, quantitation ^32^P-H1 band intensity. Data are shown as mean ± s.d. (n = 3) and *P* value was determined by one-sided sum-of-squares *f*-test. **e**, HCC1569 cells were treated with the indicated doses of RP-6306 for 2 h and subjected to the AlphaLISA assay using CDK1-pT14, CDK1-pY15 and total CDK1 antibodies. Data are shown as mean ± s.d. (n = 3). **f**, FT282-hTERT *TP53*^*R175H*^ parental and *CCNE1-*high cells were treated with the indicated doses of RP-6306 and AZD-1775 for 4 h. Whole cell lysates were prepared and immunoblotted with antibodies against CDK1-pT14, CDK1-pY15, CDK1 and vinculin as a loading contol. Representative of two immunoblots. **g,h,k**, EC50 determination for growth inhibition for the parental and *CCNE1*-high cells in the (**g**) RPE1-hTERT *TP53 *^−/−^ Cas9 (RPE1) (**h**) FT282 *TP53*^*R175H*^ (FT282) and (**k**) indicated cancer cell lines treated with the indicated compounds. Growth was monitored with an Incucyte for up to 6 population doublings. Data are shown as mean ± s.d. (n = 3). In (**k**) cell lines are also grouped according to their *CCNE1* or *BRCA* status and the red bar indicates the mean of each grouping. **i**, Whole cell lysates of FT282-hTERT *TP53*^*R175H*^ parental and *CCNE2-2A-GFP* expressing cells were immunoblotted with cyclin E2 and KAP1 specific antibodies. Representative of two immunoblots. **j**, Clonogenic survival of FT282-hTERT *TP53*^*R175H*^ *CCNE2-2A-GFP (CCNE2)* and WT parental cells treated with RP-6306. Shown on top are representative images of plates stained with crystal violet and bottom is the quantitation. Data are shown as mean ± s.d. (n = 3). For gel source data, see Supplementary Fig. 1. Source data

Using a structure-guided medicinal chemistry approach, we identified RP-6306 (Fig 2a), a highly selective inhibitor of PKMYT1 that has desirable pharmacological properties and is orally bioavailable^25^ (Supplementary Table 3). RP-6306 inhibits PKMYT1 catalytic activity at a half-maximal inhibitory concentration (IC_50_) of 3.1 ± 1.2 nM (Fig. 2b), whereas RP-6421, a closely related analogue that was predicted to be inactive, had no effect (Extended Data Fig. 2a, b). Using nanoBRET^26^, we found that RP-6306 has a cellular target engagement half-maximal effective concentration (EC_50_) with PKMYT1 of 2.5 ± 0.8 nM, 1,920-fold lower than that of WEE1 (EC_50_ of 4.8 ± 2.0 μM; Fig. 2c). RP-6306 treatment led to activation of CDK1 kinase—but not CDK2 kinase—in FT282 *CCNE1-*high cells, whereas WEE1 inhibition by AZD-1775 led to activation of both kinases, as expected (Extended Data Fig. 2c, d). In line with PKMYT1 phosphorylating primarily CDK1 Thr14, RP-6306 has an IC_50_ for reducing CDK1-pT14 of 7.5 ± 1.8 nM, whereas for CDK1-pY15, the IC50 is over 2 μM (Extended Data Fig. 2e, f). The *CCNE1*-high cell lines in both the RPE1 and FT282 backgrounds were selectively sensitive to PKMYT1 inhibition, whereas the WEE1 inhibitor (AZD-1775) and two partially selective CDK2 inhibitors (dinaciclib and PF-0687360) did not show consistent *CCNE1* level-dependent sensitivity (Fig. 2d, e, Extended Data Fig. 2g, h). Increasing *CCNE2* dosage in FT282 cells did not lead to RP-6306 sensitivity (Extended Data Fig. 2i, j). The pharmacological inhibition of PKMYT1 therefore recapitulates the synthetic lethality caused by *PKMYT1* loss in *CCNE1*-high cell lines.

## RP-6306 inhibits *CCNE1*-amplified cell growth

To test whether PKMYT1 displayed the same selectivity against *CCNE1* amplification in tumour-derived cell lines, we next assembled a panel of nine cell lines: three with amplification or gain of the *CCNE1* locus (HCC1569, SNU8 and OVCAR3), three with *BRCA1* or *BRCA2* biallelic mutations that are common in ovarian cancer (SUM149PT, COV362 and DOTC24510), and three that are wild type for *CCNE1*,* BRCA1* and *BRCA2* (KYSE30, TOV112D and NUGC3). For each cell line, we measured the EC_50_ values for RP-6306, AZD-1775, dinaciclib and PF-0687360 treatment. We found that RP-6306 was, on average, 14.1-fold more cytotoxic to the *CCNE1*-amplified cell lines, with EC_50_ values ranging from 26 to 93 nM. By contrast, the WEE1 and CDK2 inhibitors displayed blunted or absent selectivity towards *CCNE1*-amplified cell lines (Fig. 2f, Extended Data Fig. 2k). We conclude that PKMYT1 inhibition is selectively cytotoxic to tumour cells displaying *CCNE1* amplification, consistent with the genetic observations made in the isogenic cell lines.

## PKMYT1 inhibition causes DNA damage

**Fig. 3:**
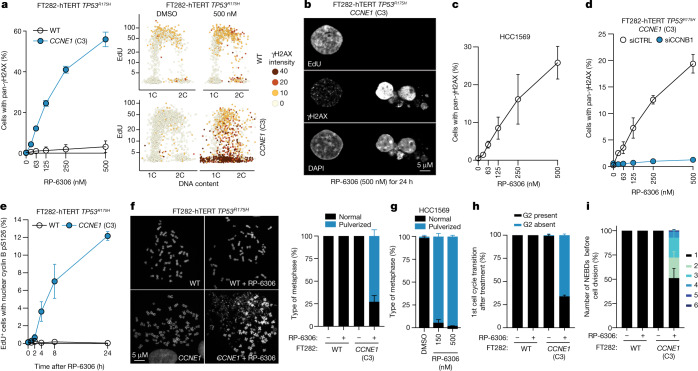
PKMYT1 inhibition causes unscheduled CDK1 activation and mitotic entry in CCNE1-high cells. **a**, QIBC analysis of γH2AX nuclear intensity, EdU incorporation and DNA content (measured with DAPI) in FT282-hTERT *TP53*^*R175H*^ cell lines. Representative QIBC (right) and quantification (left) of cells with pan-γH2AX. **b**, Representative micrograph showing EdU staining, γH2AX localization and nuclear morphology of FT282 *CCNE1*-high cells. Representative of *n* = 3 experiments. **c**, QIBC quantification of HCC1569 cells with pan-γH2AX as a function of RP-6306 dose. **d**, Quantification of FT282-hTERT *TP53*^*R175H*^ *CCNE1-*high cells transfected with siRNAs targeting cyclin B1 (siCCNB1) or non-targeting siRNA (siCTRL) with pan-γH2AX as a function of RP-6306 dose. **e**, QIBC quantification of FT282-hTERT *TP53*^*R175H*^ cells of the indicated genotype positive for EdU and cyclin B (CCNB1) pS126 as a function of RP-6306 dose. **f**, **g**, RP-6306 induces chromosome pulverization. Representative micrographs of metaphase spreads of FT282 parental (WT) and *CCNE1-*high cells left untreated or following treatment with either RP-6306 (500 nM) for 24 h (**f**, left) and quantification of FT282 cells (**f**, right) and HCC1569 cells (**g**) with pulverized chromosomes with at least 40 metaphases counted per replicate. **h**, **i**, Quantification of the first observed G2 phase (**h**) and the number of nuclear envelope breakdowns (NEBDs) before the first observed cell division (**i**) using time-lapse imaging of FT282-hTERT *TP53*^*R175H*^ PCNA–chromobody–TagRFP (WT) and *CCNE1-*high (*CCNE1*) cells treated with DMSO or RP-6306 (500 nM) for 23 h. QIBC validation is shown in Extended Data Figs. 3a, 4a, e, f, h, i. Data in **a**, **c**–**i** are mean ± s.d. (*n* = 3). Source data

**Extended Data Fig. 3:**
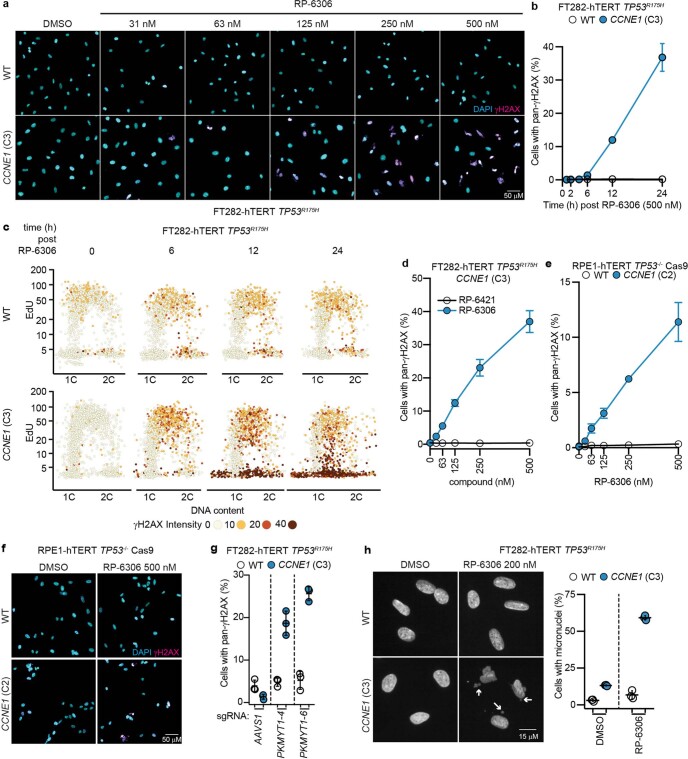
RP-6306 induces DNA damage specifically in *CCNE1*-high cells. **a**, Representative QIBC micrographs of FT282-hTERT *TP53*^*R175H*^ (WT) and *CCNE1-*high (*CCNE1*) cells treated either with DMSO or increasing doses of RP-6306. The DAPI (cyan) and γH2AX (magenta) channels are merged. **b, c**, QIBC analysis of γH2AX nuclear intensity, EdU incorporation and DNA content (DAPI) with the quantitation of cells with pan-γH2AX as a function of time after addition of RP-6306 (500 nM) shown in (**b**) and representative QIBC plots shown in (**c**). Data are shown as mean ± s.d. (n = 3). **d**, QIBC quantitation of FT282-hTERT *TP53*^*R175H*^ *CCNE1-*high cells with pan-γH2AX as a function of RP-6306 and RP-6421 dose. Data are shown as mean ± s.d. (n = 3). **e, f**, QIBC analysis of γH2AX nuclear intensity of RPE1-hTERT *TP53*^*-/-*^ Cas9 parental (WT) and *CCNE1-2A-GFP* (*CCNE1*) cells. Quantitation of cells with pan-γH2AX as a function of RP-6306 dose is shown in (**e**) and representative micrographs of cells treated with DMSO or 500 nM RP-6306 with the DAPI (cyan) and γH2AX (magenta) channels merged shown in (**f**). Data are shown as mean ± s.d. (n = 3). **g**, QIBC Quantitation of cells with pan-γH2AX after transduction with lentivirus expressing the indicated sgRNAs. **h**, RP-6306 induces micronucleation in FT282 *CCNE1*-high cells. Left, representative micrographs of cells with micronuclei (white arrows) in FT282 parental and *CCNE1-*high cells following treatment with either DMSO or RP-6306 (200 nM) for 72 h. Right, quantitation of cells with micronuclei. Data are shown as mean ± s.d. (n = 3). Source data

**Extended Data Fig. 4:**
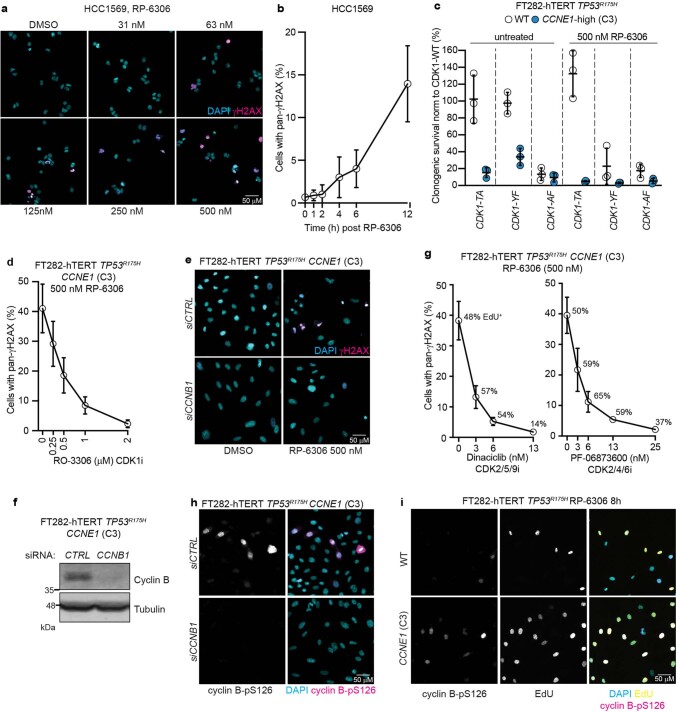
RP-6306 activates cyclin B-CDK1. **a**, Representative QIBC micrographs of HCC1569 cells treated with increasing doses of RP-6306. The DAPI (cyan) and γH2AX (magenta) channels are merged. **b**, QIBC quantitation of pan-γH2AX staining as a function of time after addition of RP-6306 (500 nM) in HCC1569 cells. Data are shown as mean ± s.d. (n = 3). **c**, Clonogenic survival assays of the indicated FT282-hTERT *TP53*^*R175H*^ Cas9 cell lines transduced with lentivirus expressing *CDK1-T14A-GFP*, *CDK1-Y15F-GFP* or *CDK1*-*T14A/Y15F-GFP* relative to WT *CDK1-GFP*. Data are shown as mean ± s.d. (n = 3). **d**, QIBC quantitation of pan-γH2AX staining in FT282-hTERT *TP53*^*R175H*^ *CCNE1* cells treated with RP-6306 (500 nM) as a function of CDK1 inhibitor RO-3306 dose. Data are shown as mean ± s.d. (n = 3). **e, f**, FT282-hTERT *TP53*^*R175H*^ *CCNE1* cells transfected with either non-targeting siRNA (*siCTRL*) or siRNA targeting cyclin B (*siCCNB1)* were treated with RP-6306 (500 nM). Representative QIBC micrographs with DAPI (cyan) and γH2AX (magenta) channels merged are shown in (**e**). Immunoblot analysis of cyclin B levels in lysates prepared from DMSO-treated cells is shown in (**f**). Tubulin was used as a loading control and are representative of three immunoblots **g**, QIBC quantitation of pan-γH2AX staining in FT282-hTERT *TP53*^*R175H*^ *CCNE1* treated with RP-6306 (500 nM) as a function of the dose of dinaciclib or (left) PF-06873600 (right). Data are shown as mean ± s.d. (n = 3). **h**, Representative QIBC micrographs of FT282-hTERT *TP53*^*R175H*^ (WT) and *CCNE1-*high (*CCNE1*) cells transfected with the indicated siRNA and stained with DAPI (cyan) and cyclin B-pS126 antibody (magenta). The channels are merged and image represents one replicate. **i**, Representative QIBC micrographs of FT282-hTERT *TP53*^*R175H*^ (WT) and *CCNE1-*high (*CCNE1*) cells treated with RP-6306 (500 nM) and stained with DAPI (cyan), EdU (yellow) and a cyclin B-pS126 antibody, (magenta). The channels are merged and image is representative of three replicates. For gel source data, see Supplementary Fig. 1. Source data

We next assessed whether PKMYT1 inhibition led to DNA damage in *CCNE1*-high cells by monitoring γH2AX levels using quantitative image-based cytometry^27^ (QIBC). Treatment of these cells with RP-6306 showed that PKMYT1 inhibition led to a dose- and time-dependent accumulation of γH2AX-positive cells solely in the *CCNE1*-high cells in both FT282 and RPE1 backgrounds, whereas the inactive analogue RP-6421 had no effect (Fig. 3a, Extended Data Fig. 3a–f). Induction of γH2AX in *CCNE1*-high FT282 cells was recapitulated using sgRNA guides targeting *PKMYT1* (Extended Data Fig. 3g). Further examination of the γH2AX^+^ population showed that it had DNA content between 1C and 2C but was EdU-negative, indicating that the cells were not actively replicating DNA (Fig. 3a). Imaging of the γH2AX-positive cells by microscopy revealed a pan-nuclear γH2AX instead of punctate foci, and fragmented or multilobular nuclei (Fig. 3b). We also observed high levels of micronucleation in FT282 *CCNE1*-high cells treated with RP-6306 (Extended Data Fig. 3h), consistent with PKMYT1 inhibition causing genome instability. RP-6306 treatment induced pan-γH2AX in an HCC1569 breast cancer cell line, indicating that tumour-derived *CCNE1* amplification also renders cells vulnerable to DNA damage induction following PKMYT1 inhibition (Fig. 3c, Extended Data Fig. 4a, b).

To assess whether the lethality in FT282 *CCNE1*-high cells caused by RP-6306 treatment was due to the activation of CDK1 driven by PKMYT1 inhibition, we expressed CDK1 variants that remove its inhibitory sites on Thr14 or Tyr15 (CDK1(T14A), CDK1(Y15F) or CDK1(T14A/Y15F)) or, as a control, wild-type CDK1, in these cells. Expression of CDK1(T14A) or CDK1(Y15F) blunted the clonogenic potential of *CCNE1*-high cells but not of their parental counterpart (Extended Data Fig. 4c). Expression of CDK1(T14A), which cannot be phosphorylated by PKMYT1, had the largest effect on the viability of *CCNE1*-high cells. These data indicate that loss of negative regulation of CDK1 by PKMYT1 is toxic in *CCNE1*-high cells. Expression of CDK1(T14A/Y15F) or treatment of cells expressing CDK1(Y15F) with RP-6306 was lethal regardless of *CCNE1* levels, suggesting that complete lack of CDK1 inhibitory phosphorylation is detrimental to cell viability irrespective of genotype (Extended Data Fig. 4c).

In parallel experiments, we found that co-treatment of cells with the CDK1 inhibitor RO-3306 abolished RP-6306-dependent pan-γH2AX induction in a dose-dependent manner (Extended Data Fig. 4d). Similarly, depletion of cyclin B1 (encoded by *CCNB1*) blocked γH2AX induction, suggesting that CDK1 activity causes DNA damage in *CCNE1*-high cells (Fig. 3d, Extended Data Fig. 4e, f). Induction of γH2AX was also dampened by treatment with dinaciclib and PF-06873600 at concentrations that allow for S phase entry, consistent with cyclin E-driven CDK2 activity also being necessary for damage induction (Extended Data Fig. 4g). However, the lack of selectivity of these inhibitors over other CDKs means that we cannot fully exclude the contribution of other kinases to the phenotype.

## PKMYT1 safeguards unscheduled mitosis

**Extended Data Fig. 5:**
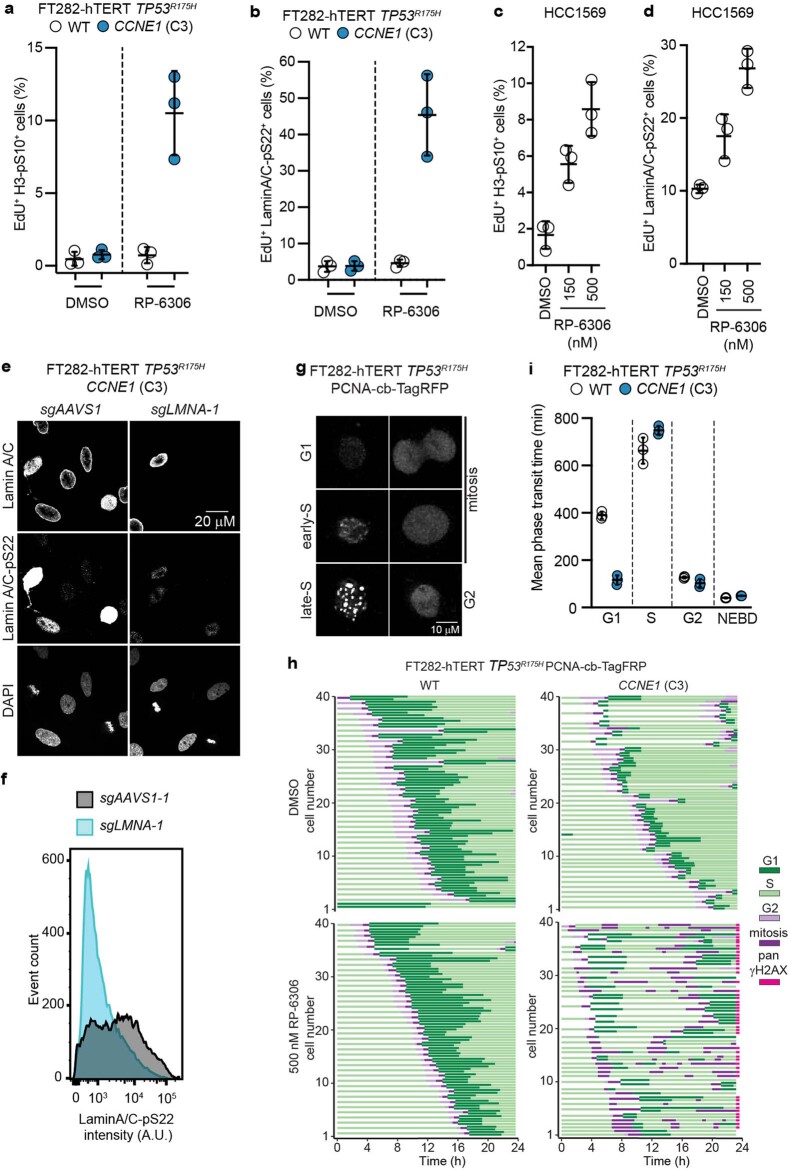
RP-6306 causes unscheduled mitosis in *CCNE1*-high cells. **a,b**, Quantitation of double-positive staining for EdU and either histone H3-pS10 (**a**) or Lamin A/C-pS22 (**b**) by FACS following vehicle (DMSO) or RP-6306 treatment (500 nM) for 24 h. Data are shown as mean ± s.d. (n = 3). The gating strategy is shown in Supplementary Figure 2. **c,d**, Quantitation of double-positive staining for EdU and either histone H3-pS10 (**c**) or Lamin A/C-pS22 (**d**) by FACS in HCC1569 cells following vehicle (DMSO) or the indicated dose of RP-6306 for 24 h. Data are shown as mean ± s.d. (n = 3). The gating strategy is shown in Extended Data Fig. 6b. **e,f**, Validation of the lamin A/C-pS22 antibody. (**e**) Representative micrographs of FT282-hTERT *TP53*^*R175H*^ *CCNE1* cells transduced with lentivirus expressing sgRNAs targeting either *AAVS1* (*sgAAVS1*) or *LMNA* (*sgLMNA-1*) stained with DAPI and the indicated antibodies. (**f**) Flow cytometry histograms of the same cells showing loss of Lamin A/C-pS22 signal in the *sgLMNA-1* condition. **g–i**, Time-lapse imaging of FT282-hTERT *TP53*^*R175H*^ PCNA-chromobody-TagRFP (WT) and *CCNE1-*high (*CCNE1*) cells treated with DMSO or RP-6306 (500 nM) for 23 h. (**g**) Pattern of PCNA localization used to identify cell cycle stages. (**h**) Cell cycle profile of individual cells (each bar represents one cell). 30 cells were analysed at random for each condition. (**i**) Quantitation of cell cycle phase transit time of indicated samples treated with DMSO for 23 h. Data are shown as mean ± s.d. (n = 3). Source data

We posited that the pan-γH2AX terminal phenotype could be secondary to a premature entry into mitosis while cells are still undergoing DNA replication, a phenomenon previously described for both WEE1 inhibition^28,29^ and in the *Cdk1*^*AF/AF*^ mouse^30^. Cyclin B–CDK1 accumulates in the cytoplasm in interphase before its rapid activation, which is linked to nuclear translocation and autophosphorylation at the onset of prophase^31,32^. We observed that upon RP-6306 treatment, FT282 *CCNE1*-high cells, but not their parental counterpart, accumulate nuclear cyclin B phosphorylated at Ser126 (pS126) in EdU-positive cells (Fig. 3e, Extended Data Fig. 4h, i). Furthermore, following PKMYT1 inhibition in both FT282 *CCNE1*-high and HCC1569 cells, a portion of EdU-positive cells showed evidence of premature entry into mitosis, as measured by histone H3 Ser10 phosphorylation (H3-pS10) and lamin A/C Ser22 phosphorylation (Lamin A/C-pS22; Extended Data Fig. 5a–f, Supplementary Fig. 2). PKMYT1 inhibition also induced chromosome pulverization, a phenotype that was completely dependent on high *CCNE1* levels (Fig. 3f). Chromosome pulverization is associated with premature mitotic entry of actively replicating cells^33^ and was also observed in HCC1569 cells (Fig. 3g).

To characterize the effect of PKMYT1 inhibition on mitotic entry, we carried out time-lapse microscopy of cells expressing a PCNA chromobody fused to TagRFP^34^. By combining PCNA-localization dynamics with nuclear envelope breakdown to mark mitotic entry (Extended Data Fig. 5g), we measured cell cycle phase transit times in cells incubated with RP-6306 or DMSO as control (Supplementary Videos 1–4). Compared with their parental cells, DMSO-treated FT282 *CCNE1*-high cells display shortened G1 phase and a slightly extended S phase (Extended Data Fig. 5h, i). The majority of *CCNE1*-high cells treated with RP-6306 entered the first mitosis before completion of DNA replication, whereas most parental cells had a clear G2 phase (Fig. 3h, Extended Data Fig. 5h). Many of the *CCNE1*-high cells that skipped G2 in response to PKMYT1 inhibition did not go through a normal cell division but rather toggled between mitotic and interphase before terminating with high pan-γH2AX signal (Fig. 3i, Extended Data Fig. 5h). This phenotype is reminiscent of that observed in cells expressing the constitutively active CDK1(T14A/Y15F) mutant^35^. We conclude that PKMYT1 inhibition triggers unscheduled mitotic entry selectively in *CCNE1*-high cells. The lack of premature mitotic entry in parental FT282 cells following PKMYT1 inhibition is consistent with the observation that *PKMYT1* depletion does not trigger unscheduled mitotic entry in HeLa cells^36,37^.

## CCNE1 activates MMB–FOXM1

**Fig. 4:**
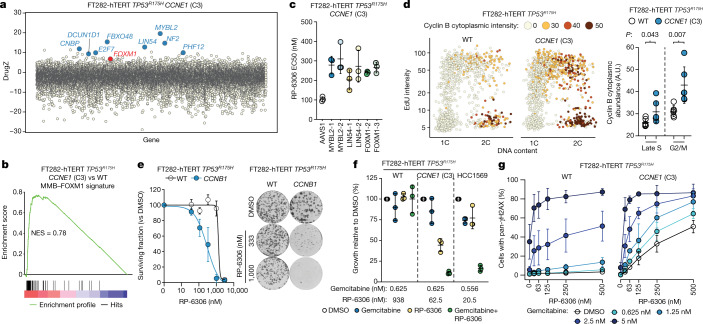
Replication stress and FOXM1–MMB activity underlie vulnerability to PKMYT1 inhibition. **a**, RP-6306 resistance screen for dose required to kill 80% of cells (LD_80_) performed in FT282-hTERT *TP53*^*R175H*^ *CCNE1* (C3 and C4) with DrugZ scores for C3 plotted. Genes with DrugZ > 9 in both C3 and C4 screens (blue) and *FOXM1* (red) are shown for reference. **b**, Gene set enrichment analysis (GSEA) of differential gene expression in FT282 parental (WT) versus *CCNE1*-high (C3) cells for genes co-regulated by MMB–FOXM1. **c**, EC_50_ values for RP-6306 in *CCNE1*-high FT282-hTERT *TP53*^*R175H*^ cells nucleofected with Cas9 ribonucleoproteins assembled with the indicated sgRNAs. Growth was monitored by clonogenic survival assay. **d**, QIBC analysis of cyclin B cytoplasmic intensity, EdU incorporation and DNA content (measured with DAPI). Representative QIBC plots (left) and cytoplasmic cyclin B intensity (right) quantification in late S or G2/M. *P* values determined by two-tailed *t*-test. **e**, Clonogenic survival of FT282-hTERT *TP53*^*R175H*^ *CCNB1-2A-GFP* and wild-type parental cells treated with RP-6306. Quantification (left) and representative images of plates stained with crystal violet (right). **f**, Growth inhibition relative to DMSO control of parental (WT) and *CCNE1-*high FT282-hTERT *TP53*^*R175H*^ cells and HCC1569 cells after the indicated treatments. Growth was monitored with an Incucyte live-cell imager for up to six population doublings. **g**, QIBC quantification of cells with pan-γH2AX in response to the indicated RP-6306–gemcitabine combinations. Data in **c**–**g** are mean ± s.d. (*n* = 3). Source data

**Extended Data Fig. 6:**
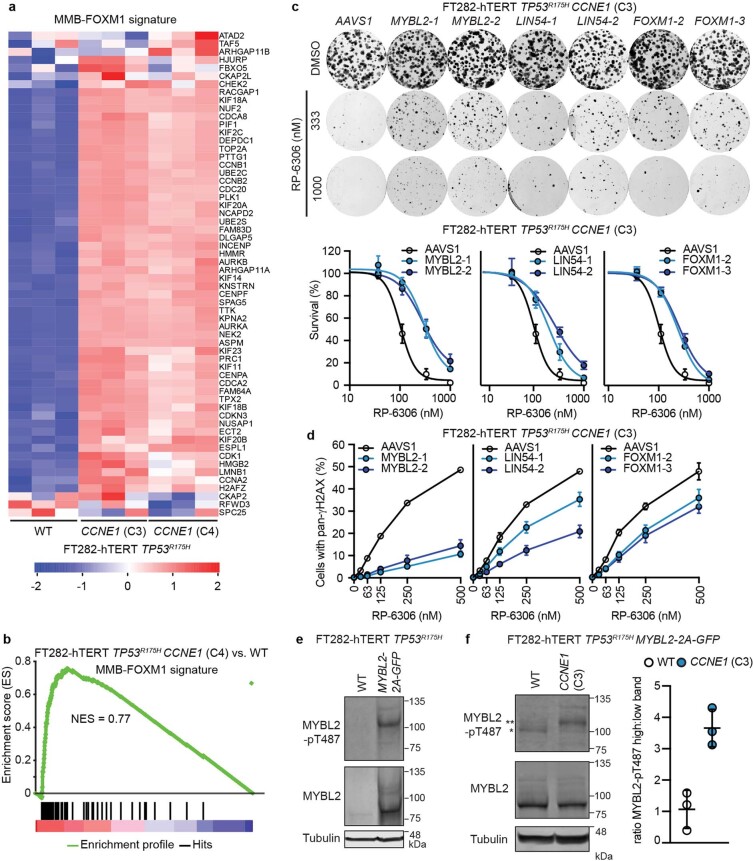
MMB-FOXM1 is hyperactivated in *CCNE1*-high cells. **a,b**, heat map (**a**) and gene set enrichment analysis (GSEA) (**b**) of differential gene expression in FT282 parental (WT) vs *CCNE1*-high (C3 and C4 for heat map, C4 for GSEA) cells for a gene set comprising genes co-regulated by MMB-FOXM1. Development of the co-regulated MMB-FOXM1 gene list is available in Methods and the list of genes is available in Extended Data Table 3. **c**, Clonogenic survival of *CCNE1*-high FT282-hTERT *TP53*^*R175H*^ cells nucleofected with Cas9 ribonucleoproteins assembled with the indicated sgRNAs. Shown on top are representative images of plates stained with crystal violet and below is the quantitation. Data are shown as mean ± s.d. (n = 3). **d**, QIBC quantitation of cells with pan-γH2AX as a function of RP-6306 dose of *CCNE1*-high FT282-hTERT *TP53*^*R175H*^ cells nucleofected with Cas9 ribonucleoproteins assembled with the indicated sgRNAs. Data are shown as mean ± s.d. (n = 3). **e,f**, Whole cell lysates of FT282-hTERT *TP53*^*R175H*^ parental and *MYBL2-2A-GFP* expressing (**e**) and FT282-hTERT *TP53*^*R175H*^ *MYBL2-2A-GFP* WT and *CCNE1*-high cells (**f**, left) were immunoblotted with MYBL2, MYBL2-pT487 and Tubulin specific antibodies. (**f**, left) * and ** denote the lower and upper phosphorylated MYBL2 bands respectively. (**f**, right) Quantitation of upper to lower band intensity. Data are shown as mean ± s.d. (n = 3). (**e**) Representative of two immunoblots. For gel source data, see Supplementary Fig. 1. Source data

To understand how *CCNE1* overexpression leads to vulnerability to PKMYT1 inhibition, we conducted a CRISPR-based RP-6306-resistance screen in FT282 *CCNE1*-high cells. This screen found that mutations in *MYBL2*, *LIN54*, *E2F7*, *FBXO48*, *DCUN1D1*, *CNBP*, *NF2* and *PHF12* engender resistance to RP-6306 in both of the *CCNE1*-high clones that we screened (Fig. 4a and Supplementary Table 1). *MYBL2*, *LIN54* and *FOXM1* were of interest as they encode members of the MYBL2–MuvB (also known as MMB)–FOXM1 complex that regulates the expression of *CCNB1*,* CDK1* and other mitotic genes^38^. Transcriptome profiling of the isogenic pair of FT282 cells revealed that the MMB–FOXM1 transcriptional program was activated in *CCNE1*-high cells (Fig. 4b, Extended Data Fig. 6a, b, Supplementary Table 4). We validated that sgRNA-mediated disruption of *MYBL2*, *LIN54* or *FOXM1* increased the EC_50_ of RP-6306 in FT282 *CCNE1*-high cells with a concomitant decrease in pan-γH2AX cells (Fig. 4c, Extended Data Fig. 6c, d). These data suggest that transcriptional regulation by MMB–FOXM1 contributes to the sensitivity of *CCNE1*-high cells to PKMYT1.

As CDK2 phosphorylation drives MYBL2 transactivation^39^, we assessed whether *CCNE1* levels affect the MMB–FOXM1 complex in FT282 cells. To circumvent challenging low levels of MYBL2 expression, we overexpressed MYBL2 (Extended Data Fig. 6e). Analysis of MYBL2 by immunoblotting revealed slow migrating bands that were recognized by a MYBL2 pT487 antibody (Extended Data Fig. 6f). The *CCNE1*-driven phosphorylation of MYBL2 therefore links cyclin E and CDK1 activity.

**Extended Data Fig. 7:**
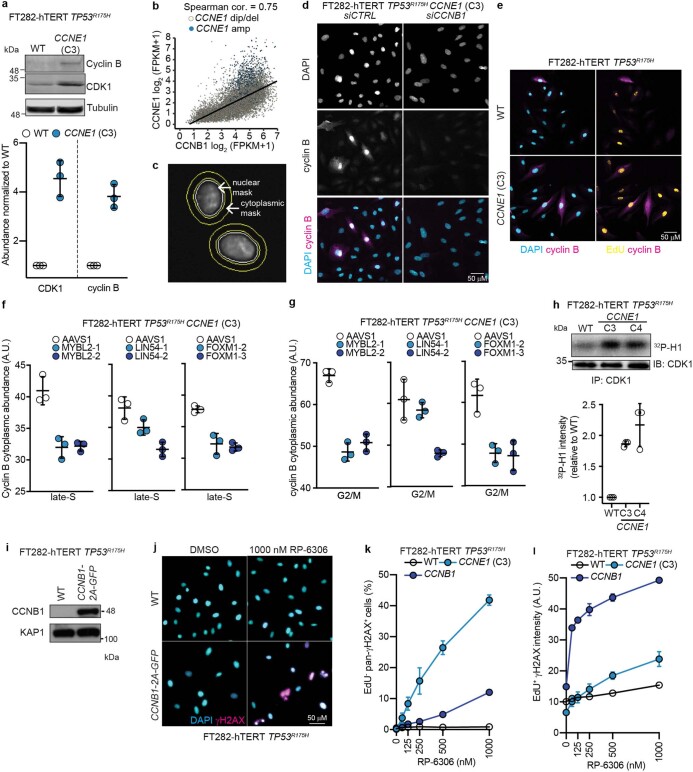
Increased Cyclin B and CDK1 abundance and activity render cell sensitive to RP-6306. **a**, Whole cell lysates of FT282-hTERT *TP53*^*R175H*^ WT and *CCNE1*-high (C3) cells were immunoblotted with antibodies against cyclin B, CDK1 and Tubulin. Top is representative immunoblot images and bottom are quantitation. Data are shown as mean ± s.d. (n = 3). **b**, Correlation of CCNE1 and CCNB1 mRNA expression in TCGA pan-cancer tissues (n = 10,304). **c**, Approach for cytoplasmic cyclin B quantitation by QIBC. The nuclear mask edge was expanded by 2 and 12 pixels to create a doughnut mask used to estimate cytoplasmic signal intensity. **d**, Representative micrographs of FT282-hTERT *TP53*^*R175H*^ *CCNE1-*high cells transfected with the indicated siRNAs and stained with DAPI (cyan) and cyclin B antibodies (magenta). The channels are merged in the bottom panels. **e**, Representative QIBC micrographs of FT282-hTERT *TP53*^*R175H*^ parental (WT) and *CCNE1-*high cells stained with cyclin B antibodies (magenta) and either DAPI (cyan) or EdU (yellow) channels merged. **f,g**, QIBC analysis of cyclin B cytoplasmic intensity, EdU incorporation and DNA content (DAPI) of *CCNE1*-high FT282-hTERT *TP53*^*R175H*^ cells nucleofected with Cas9 ribonucleoproteins assembled with the indicated sgRNAs. The quantitation of cells with cytoplasmic cyclin B in late S (**f**) or G2/M (**g**) are shown. Data are shown as mean ± s.d. (n = 3) **h**, *CCNE1*-high cells have increased CDK1 activity. Top, cellular extracts of the indicated cell lines were prepared and immunoprecipitated with agarose-coupled CDK1 antibodies. Immunoprecipitates (IP) were subjected to in vitro kinases assays using [γ-^32^P]-ATP and recombinant histone H1 as a substrate. Reactions were resolved by SDS-PAGE and gels were imaged using a phosphor screen. A sample of each immunoprecipate was immunoblotted (IB) with a CDK1 antibody as loading control. Bottom, quantitation ^32^P-H1 band intensity. Data are shown as mean ± s.d. (n = 3). **i**, Whole cell lysates of FT282-hTERT *TP53*^*R175H*^ parental and *CCNB1-2A-GFP* expressing cells were immunoblotted with cyclin B and KAP1 specific antibodies. **j**, Representative QIBC micrographs of FT282-hTERT *TP53*^*R175H*^ (WT) and *CCNB1-2A-GFP* (*CCNB1*) cells treated either with DMSO or 1000 nM RP-6306. The DAPI (cyan) and γH2AX (magenta) channels are merged. **k,l**, QIBC quantitation of FT282-hTERT *TP53*^*R175H*^ WT, *CCNB1-2A-GFP* (*CCNB1*) and *CCNE1-*high (*CCNE1*) with pan-γH2AX^+^ EdU^-^ cells (**k**) and γH2AX intensity in EdU^+^ cells (**l**) as a function of RP-6306 dose. Data are shown as mean ± s.d. (n = 3). For gel source data, see Supplementary Fig. 1. Source data

We confirmed that cyclin B and CDK1, two targets of MMB–FOXM1, are upregulated at the transcript and protein levels in *CCNE1*-high cells (Extended Data Figs. 6a, 7a). Tumour *CCNB1* mRNA levels were also positively correlated with those of *CCNE1* and were particularly high in tumours with *CCNE1* amplification, suggesting that this relationship was also relevant to tumours (Extended Data Fig. 7b). We also observed that cytoplasmic cyclin B levels were increased in the late-S and G2/M phases of *CCNE1*-high cells compared with their parental counterparts (Fig. 4d, Extended Data Fig. 7c–e). Disrupting MMB–FOXM1 transcriptional activity with sgRNAs targeting *MYBL2*, *LIN54* or *FOXM1* decreased late-S and G2/M cytoplasmic cyclin B levels (Extended Data Fig. 7f, g). The build-up of cyclin B-CDK1 levels was accompanied by slightly higher levels of CDK1 activity in FT282 *CCNE1*-high cells, as measured with immune complex kinase assays, although not enough to trigger unscheduled mitotic entry (Fig. 3h, Extended Data Fig. 7h). We conclude that *CCNE1*-high cells have elevated cytoplasmic cyclin B–CDK1, suggesting it may be primed to become fully activated following PKMYT1 inhibition.

To test whether higher levels of cyclin B–CDK1 are sufficient to cause sensitivity to RP-6306, we overexpressed cyclin B from a *CCNB1-2A-GFP* transgene using the piggyBAC system in FT282 cells (Extended Data Fig. 7i). RP-6306 treatment reduced clonogenic survival of *CCNB1* overexpressing cells compared to the parental cell line (Fig. 4e). QIBC confirmed the presence of multilobular, EdU^−^ and pan-γH2AX^+^ nuclei, suggesting that cyclin B overexpression phenocopies *CCNE1*-high cells (Extended Data Fig. 7j, k). However, we noted that γH2AX intensity also increased in EdU^+^ cells (Extended Data Fig. 7l), which is reminiscent of WEE1 inhibition^40^. We conclude that the MMB–FOXM1-dependent increase in cyclin B–CDK1 expression partly explains the vulnerability of *CCNE1*-high cells to PKMYT1 inhibition, and that additional factors contribute to this vulnerability.

## Replication stress synergizes with RP-6306

**Extended Data Fig. 8:**
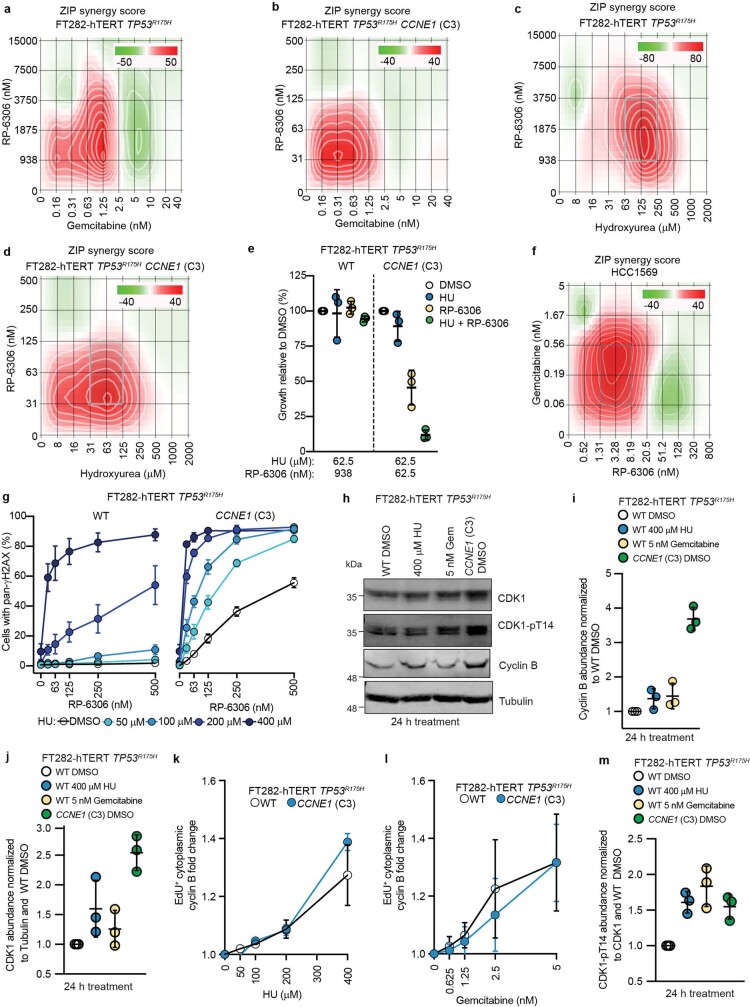
HU and gemcitabine synergize with RP-6306. **a-d,f**, ZIP synergy scores at various dose combinations of RP-6306 and gemcitabine (**a**,**b**,**f**) or hydroxyurea (HU) (**c**,**d**) in FT282-hTERT *TP53*^*R175H*^ parental (WT) (**a**,**c**), *CCNE1-*high (**b**,**d**) and HCC1569 (**f**) cells. Score ≥10 (red color) represents synergy, ≤-10 (green) represents antagonism. Values were obtained by analyzing mean data from 3 independent biological replicates with SynergyFinder. Growth was monitored with an Incucyte for up to 6 population doublings. **e**, Growth inhibition relative to DMSO control of FT282-hTERT *TP53*^R175H^ parental (WT) and *CCNE1-*high cells after treatment with the indicated dose of hydroxyurea (HU), RP-6306 or the combination of both. Growth was monitored with an Incucyte for up to 6 population doublings. Data are shown as mean ± s.d. (n = 3). **g**, QIBC quantitation of FT282-hTERT *TP53*^R175H^ parental (WT) and *CCNE1*-high cells with pan-γH2AX in response to the indicated RP-6306/HU combinations. Data are shown as mean ± s.d. (n = 3). **h–j, m**, Replication stress activates phosphorylation of CDK1-T14. (**h**) Whole cell lysates of the indicated cells and conditions were immunoblotted with CDK1, CDK1-pT14, cyclin B and Tubulin specific antibodies. Quantitation of cyclin B (**i**), CDK1 (**j**) and CDK1-pT14 (**m**). Data are shown as mean ± s.d. (n = 3). **k, l**, QIBC quantitation of the fold-change of cytoplasmic cyclin B intensity in EdU^+^ cells following treatment with either HU (**k**) or gemcitabine (**l**) at the indicated doses for 48 h. Data are shown as mean ± s.d. (n = 3). For gel source data, see Supplementary Fig. 1. Source data

Cyclin E overexpression causes DNA replication stress and extends S phase, which may also impose a need for CDK1 inhibitory phosphorylation. We therefore tested whether agents that perturb DNA replication, such as hydroxyurea or the nucleoside analogue gemcitabine, rendered cells sensitive to PKMYT1 inhibition. We observed synergistic cytotoxicity when combining RP-6306 with either hydroxyurea or gemcitabine in both FT282 parental and *CCNE1*-high cells (Extended Data Fig. 8a–d). However, combining gemcitabine (at 0.625 nM) with 62.5 nM of RP-6306 was highly toxic in *CCNE1*-high cells whereas combining the same dose of gemcitabine with higher concentrations of RP-6306 (938 nM) was innocuous in the parental cell line (Fig. 4f). The same trend was observed for combinations of hydroxyurea and RP-6306 (Extended Data Fig. 8e) suggesting conservation of a therapeutic index between wild-type and *CCNE1*-high cells. The synergy was also observed in *CCNE1*-amplified HCC1569 cells indicating that combined dosing may afford an attractive therapeutic strategy for *CCNE1*-amplified tumours (Fig. 4f, Extended Data Fig. 8f).

Combining hydroxyurea or gemcitabine with RP-6306 in FT282 parental cells also caused a synergistic increase in pan-γH2AX cells, similar to that seen in *CCNE1*-high cells (Fig. 4g, Extended Data Fig. 8g) and analogous results were reported with WEE1 inhibition, which also activates CDK1^29,41^. Therefore, replication stress caused by increased *CCNE1* dosage may also contribute to the vulnerability to PKMYT1 inhibition.

An explanation for the above results may be that DNA replication stress renders cells susceptible to unscheduled CDK1 activation through an increase in cyclin B–CDK1 levels or activity. However, we did not detect increased levels of cyclin B or CDK1 following hydroxyurea or gemcitabine treatment (Extended Data Fig. 8h–l). CDK1 pT14 levels were increased following hydroxyurea or gemcitabine treatment, reaching levels similar to those in *CCNE1*-high cells when controlling for total CDK1 (Extended Data Fig. 8h, m). We conclude that replication stress either activates PKMYT1 to dampen CDK1 activity or that the resulting extended S phase causes a higher proportion of cyclin B–CDK1 complexes in a Thr14-phosphorylated inhibited state.

## *CCNE1*-amplified tumour inhibition in vivo

**Fig. 5:**
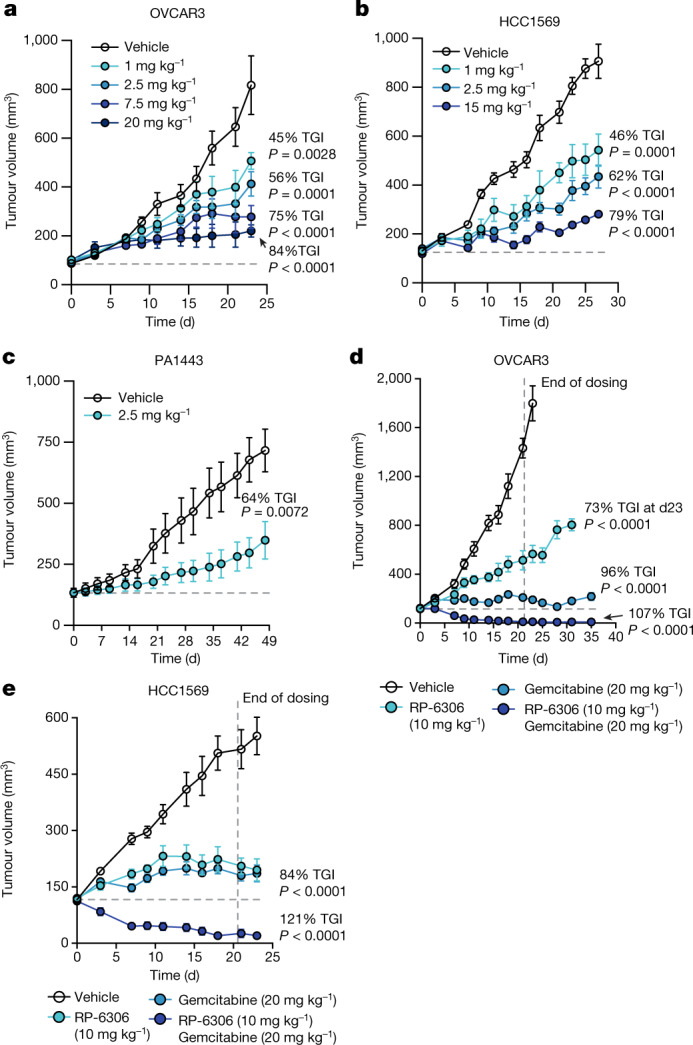
RP-6306 shows single-agent anti-tumour activity and profound tumour regressions in combination with gemcitabine. **a**, **b**, Growth of OVCAR3 (**a**) and HCC1569 (**b**) xenografts in CB-17 SCID and SCID-beige mice treated with either RP-6306 or vehicle. RP-6306 was administered orally twice daily at the indicated doses for the duration of the experiment. Results are expressed as mean tumour volume ± s.e.m. (OVCAR3 *n* = 8 (vehicle), 7 (1 mg kg^−1^), 8 (2.5 mg kg^−1^), 7 (7.5 mg kg^−1^), 8 (20 mg kg^−1^); HCC1569 *n* = 8 (vehicle),7 (1 mg kg^−1^), 8 (2.5 mg kg^−1^), 6 (15 mg kg^−1^)). Percentage tumour growth inhibition (% TGI) and *P* values relative to vehicle as determined by one-way ANOVA are shown. **c**, Tumour growth of a *CCNE1*-amplified pancreatic cancer (PA1443) patient-derived xenograft implanted in BALB/c nude mice treated either with RP-6306 or vehicle. RP-6306 was administered orally twice daily at 2.5 mg kg^−1^ for the duration of the experiment. Results are expressed as mean tumour volume ± s.e.m. (*n* = 8) with % TGI and *P* value relative to vehicle as determined by unpaired one-sided *t*-test. **d**, **e**, Tumour growth of OVCAR3 (**d**) and HCC1569 (**e**) xenografts in mice treated with either RP-6306, gemcitabine or both. Gemcitabine was administered once weekly intraperitoneally starting at day 0 and RP-6306 was given oral twice daily for 21 days after which all treatments were stopped, and tumour size was monitored for the remainder of the experiment. Results are expressed as tumour volume mean ± s.e.m. (OVCAR3 *n* = 7 (vehicle), 6 (10 mg kg^−1^ RP-6306), 7 (20 mg kg^−1^ gemcitabine), 7 (10 mg kg^−1^ RP-6306 + 20 mg kg^−1^ gemcitabine); HCC1569 *n* = 7 (vehicle), 7 (10 mg kg^−1^ RP-6306), 7 (20 mg kg^−1^ gemcitabine), 7 (10 mg kg^−1^ RP-6306 + 20 mg kg^−1^ gemcitabine)). % TGI and *P* values relative to vehicle as determined by one-way ANOVA are shown. Source data

**Extended Data Fig. 9:**
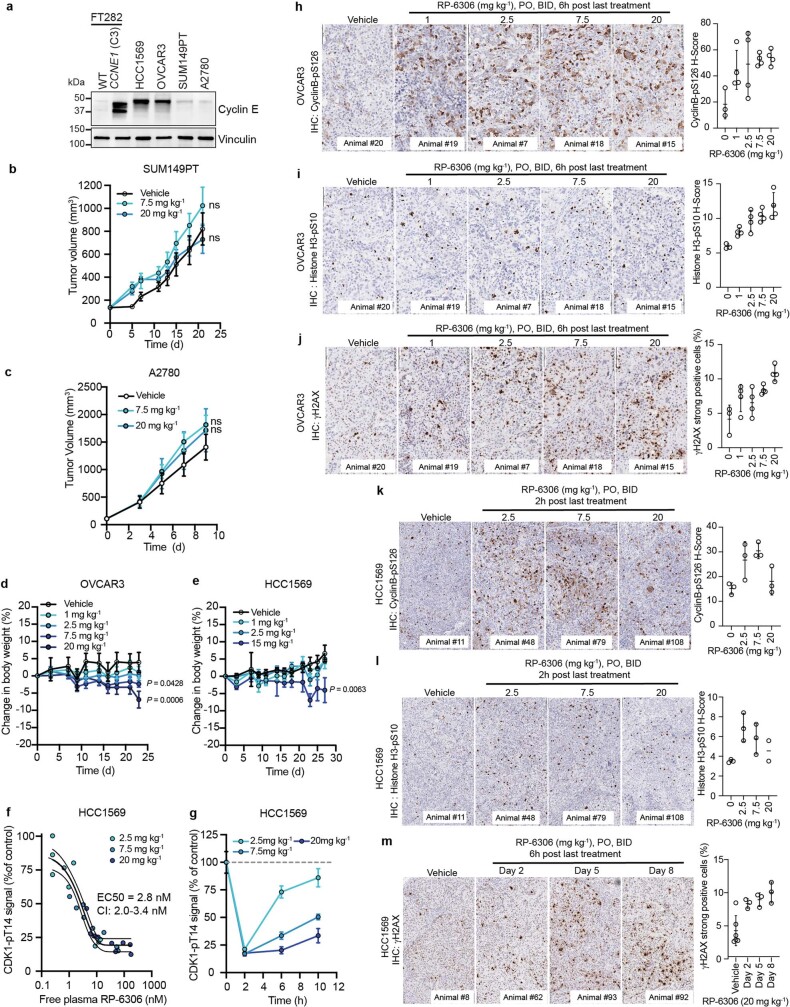
Characterization of CCNE1-normal and *CCNE1*-amplifed CDX models. **a**, Whole cell lysates of the indicated cell lines were immunoblotted with antibodies against cyclin E and vinculin. Representative of two immunoblots. **b–e**, RP-6306 was administered orally BID at the indicated doses for the duration of the experiment. **b, c**, Tumor growth of SUM149PT (**b**) and A27080 (**c**) xenografts in NOD-SCID mice treated with either RP-6306 or vehicle BID for the duration of the experiment. Results are expressed as mean tumor volume ± s.e.m. (n = 8). *P* values relative to vehicle were determined by a one-way ANOVA. n.s. = non-significant *P *value **d**, **e** Changes in body weight in tumor-bearing OVCAR3 (**d**) and HCC1569 (**e**) CB-17 SCID and SCID-beige mice treated with either RP-6306 or vehicle. Results are expressed as mean % body weight change ± s.e.m. (OVCAR3 *n* = 8 (vehicle), 7 (1 mg kg^−1^), 8 (2.5 mg kg^−1^), 7 (7.5 mg kg−1), 8 (20 mg kg^−1^); HCC1569 *n* = 8 (vehicle),7 (1 mg kg^−1^), 8 (2.5 mg kg^−1^), 6 (15 mg kg^−1^)). *P* values relative to vehicle were determined by a one-way ANOVA. **f, g** Mice bearing HCC1569 tumors were treated with RP-6306 at the indicated doses BID for 1.5 days and tumor tissue and whole blood sampled at 2, 6 and 10 h post last dose. **f**, Data represent the free (unbound to plasma protein) blood concentration of RP-6306 for mouse relative to tumor CDK1-pT14 signal inhibition quantified by ELISA relative to vehicle-treated control tumors (N = 3/group/time point). The tumor EC50 was determined by a non-linear least square’s regression to a normalized dose-response model with 95% confidence intervals. **g**, Kinetics of CDK1-pThr14 inhibition with time. Data presented as the mean ± s.e.m. (n = 3). **h–m**, OVCAR3 (**h**-**j**) and HCC1569 (**k**-**m**) tumor-bearing mice were administered the indicated dose of RP-6306 orally BID for 8 days and sacrificed at 6 h post last treatment (**h**-**j**) or administered the indicated dose of RP-6306 orally BID 1.5 days and sacrificed at 2 h post last treatment (**k**,**l**) and tumor tissue prepared for FFPE. HCC1569 tumors (**m**) were treated with 20 mg kg^−1^ RP-6306 BID for 2, 5 or 8 days and tumor tissue harvested 6 h post last treatment. Tumor tissues were stained with cyclin B-pS126 (**h**,**k**), histone H3-pS10 (**i**,**l**) or γH2AX (**j**,**m**) antibodies and the H-score (**h**,**i**,**k**,**l**) or percentage of strong positive γH2AX cells present in the tumor area (**j**,**m**) was quantified by HALO software. Results are expressed as mean ± s.e.m. (**h**-**j**: n = 3,4,4,4; **k**: n = 3,3,3,3; **l**: n = 3,3,3,2; **m**: n = 6,3,3,3). Source data

We next assessed whether RP-6306 displays anti-tumour activity first as a single-agent in tumour xenograft models. This allowed us to explore the pharmacokinetic and pharmacodynamic properties of the compound. We implanted *CCNE1*-amplified (HCC1569 and OVCAR3), *BRCA1*-mutated SUM149PT and *BRCA-* and *CCNE1*-normal A2780 cells (Extended Data Fig. 9a) to generate tumour xenografts in mice that were randomized to receive either RP-6306 or vehicle orally twice daily. We observed dose-dependent tumour growth inhibition in both HCC1569 and OVCAR3 cell lines that reached 79 and 84% tumour growth inhibition at 20 mg kg^−1^ and 15 mg kg^−1^, respectively. (Fig. 5a, b), whereas RP-6306 had no effect on the growth of SUM149PT or A2780-derived tumours at the same dose (Extended Data Fig. 9b, c). Mice at each dose level experienced less than 7% body weight loss, indicating that RP-6306 was well tolerated (Extended Data Fig. 9d, e). We observed a direct dose- and time-dependent relationship between RP-6306 plasma concentration and inhibition of CDK1 Thr14 phosphorylation in tumours (Extended Data Fig. 9f, g), with an EC_50_ of 2.8 nM (95% confidence interval 2.0–3.4 nM), indicating potent on-target activity in vivo. RP-6306 treatment caused dose-dependent increases in levels of cyclin B1 pS126 and histone H3 pS10, markers of CDK1 activity and M-phase, respectively (Extended Data Fig. 9h, i, k, l). We also observed a dose- and time-dependent increase of γH2AX following treatment with 20 mg kg^−1^ RP-6306, suggesting that cells with DNA damage accumulate in tumours over time (Extended Data Fig. 9j, m).

**Extended Data Fig. 10:**
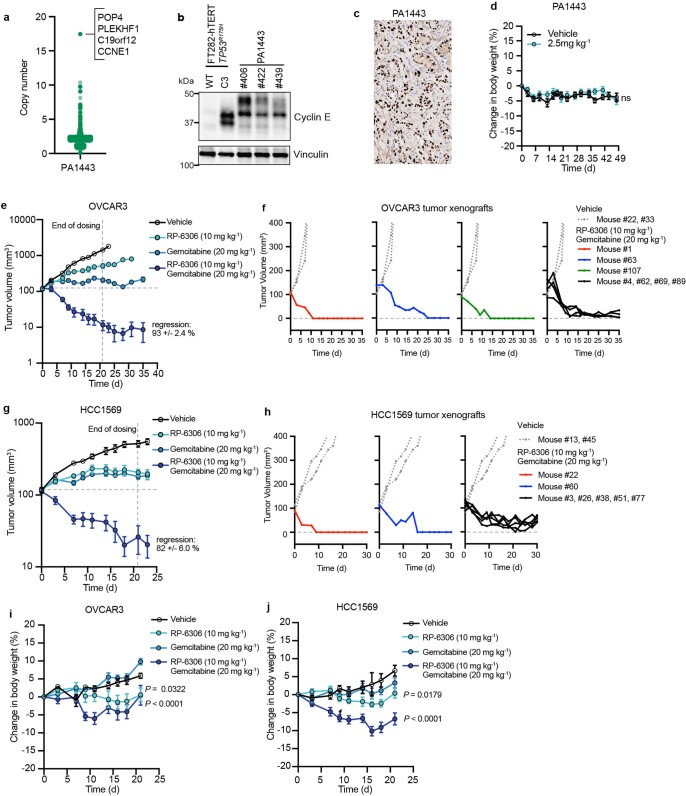
Characterization of the PDX PA1443 and RP-6306/gemcitabine in combination drive tumor regression in *CCNE1*-amplified CDX models. **a**, Distribution of the gene-level copy number in the *CCNE1*-amplified pancreatic cancer (PA1443) patient-derived xenograft (PDX). Highlighted is the amplicon containing *CCNE1*. **b**, Whole cell lysates from FT282-hTERT *TP53*^*R175H*^ parental (WT) and *CCNE1*-high (C3) cell lines and PA1443 PDX tumor tissue were immunoblotted with antibodies to cyclin E and vinculin. Representative of two immunoblots. **c**, Tumor tissues of PA1443 PDX implanted in BALB/c nude mice were prepared for FFPE and stained with a cyclin E1 antibody. **d**, Changes in body weight in tumor-bearing PA1443 PDX implanted in BALB/c nude mice treated either with RP-6306 or vehicle. RP-6306 was administered orally BID at 2.5 mg kg^−1^ for the duration of the experiment. Results are expressed as mean % body weight ± s.e.m. (n = 8). *P* value relative to vehicle was determined with a one-way ANOVA test. Only *P* values < 0.05 are indicated. ns, not significant (*P* value > 0.05). **e, g**, Same data as presented in Fig. 5d, e with tumour growth values plotted in log scale for OVCAR3 (**e**) and HCC1569 (**g**). The percent regression of the RP-6306 10 mg kg^−1^ and Gemcitabine 20 mg kg^−1^ combination arm is indicated. Results are expressed as mean tumor volume ± s.e.m. (OVCAR3 *n* = 7 (vehicle), 6 (10 mg kg^−1^ RP-6306), 7 (20 mg kg^−1^ gemcitabine), 7 (10 mg kg^−1^ RP-6306 + 20 mg kg^−1^ gemcitabine); HCC1569 *n* = 7 (vehicle), 7 (10 mg kg^−1^ RP-6306), 7 (20 mg kg^−1^ gemcitabine), 7 (10 mg kg^−1^ RP-6306 + 20 mg kg^−1^ gemcitabine)). Please refer to Fig. 5 for TGI and *P* values. **f, h**, Growth traces for individual OVCAR3 (**f**) and HCC1569 (**h**) tumors from the experiments shown in Fig. 5d, e from mice treated with the gemcitabine/RP-6306 combination. For comparison, tumor growth data for two mice treated with vehicle are shown. **i, j**, Changes in body weight in tumor-bearing OVCAR3 (**i**) and HCC1569 (**j**) CB-17 SCID and SCID-beige mice treated with either RP-6306, gemcitabine or both. Gemcitabine was delivered once weekly intraperitoneally and RP-6306 was given orally BID for 21 d after which all treatments were stopped, and body weight monitored for the remainder of the experiment. Results are expressed as mean ± s.e.m. (OVCAR3 *n* = 7 (vehicle), 6 (10 mg kg^−1^ RP-6306), 7 (20 mg kg^−1^ gemcitabine), 7 (10 mg kg^−1^ RP-6306 + 20 mg kg^−1^ gemcitabine); HCC1569 *n* = 7 (vehicle), 7 (10 mg kg^−1^ RP-6306), 7 (20 mg kg^−1^ gemcitabine), 7 (10 mg kg^−1^ RP-6306 + 20 mg kg^−1^ gemcitabine)) . Also indicated are *P* values relative to vehicle were determined with a one-way ANOVA test. Only *P* values < 0.05 are indicated. For gel source data, see Supplementary Fig. 1. Source data

The single-agent activity of RP-6306 was also tested in a patient-derived xenograft (PDX) model obtained from a pancreatic adenocarcinoma (model PA1443). This tumour displays moderate *CCNE1* amplification (Extended Data Fig. 10a) and increased cyclin E protein levels (Extended Data Fig. 10b, c). PA1443 tumours also harbour *TP53* loss-of-function (G245S) and *KRAS* activating (G12D) mutations. Twice-daily dosing of RP-6306 at 2.5 mg kg^−1^ led to a dose-dependent tumour growth inhibition, reaching 64% over 48 days (Fig. 5c) with less than 5% loss of body weight (Extended Data Fig. 10d). Together, these data indicate that PKMYT1 inhibition displays single-agent tumour growth inhibition in a variety of *CCNE1*-amplified models.

The observation that replication stress can sensitize *CCNE1*-high tumour cells to PKMYT1 inhibition prompted us to test whether gemcitabine synergized with RP-6306 in vivo. We tested a dosing regimen in which gemcitabine was delivered intraperitoneally once weekly and RP-6306 was given orally twice daily for 21 days. The combined treatment with gemcitabine and RP-6306 led to profound and durable tumour regressions in both OVCAR3 and HCC1569 tumours, with tumours showing no sign of regrowth for up to 15 days following cessation of treatment (Fig. 5d, e, Extended Data Fig. 10e–h). At day 35, termination of the OVCAR3 model, 3 out of 7 mice were tumour-free (Extended Data Fig. 10f) and in the HCC1569 tumour group, 2 out of 7 mice remained tumour-free at the termination of the experiment on day 30 (Extended Data Fig. 10h). These results indicate a profound tumour response in both models. A maximum of 10% loss in body weight was observed in the combination arm demonstrating tolerability of the combination (Extended Data Fig. 10i, j). We conclude that enhancing DNA replication stress in *CCNE1*-high tumours may be an effective approach to drive tumour regression in combination with PKMYT1 inhibition.

## Discussion

Oncology drug discovery based on the identification of synthetic lethal interactions holds great promise but very few drug candidates have so far been developed, ab initio, using this approach. In this Article, we present how a genome-scale genetic interaction screen in a cellular model of *CCNE1* amplification led to the identification of a vulnerability to PKMYT1 inhibition and report the discovery of RP-6306, a selective inhibitor of the PKMYT1 kinase that inhibits *CCNE1*-amplified cell and tumour growth. RP-6306 is orally bioavailable and recently entered first-in-human clinical studies as monotherapy (ClinicalTrials.gov identifier NCT04855656) and in combination with gemcitabine (ClinicalTrials.gov identifier NCT05147272) or FOLFIRI (ClinicalTrials.gov identifier NCT05147350). This work further demonstrates the applicability of uncovering oncology drug targets from genetic interaction screens.

We propose a model in which the basis for the observed synthetic lethality between PKMYT1 is the result of a two-stage activation, in which both *CCNE1*-driven DNA replication stress and MMB–FOXM1 transcription increase cyclin B–CDK1 levels and activity in S phase, rendering cells highly vulnerable to the loss of PKMYT1-driven inhibitory CDK1 Thr14 phosphorylation (Supplementary Fig. 3). The resulting activation of CDK1 causes unscheduled mitotic entry and mitotic-interphase oscillations that are associated with catastrophic genome instability. We note that prior to mitosis, the cyclin B–CDK1 complex accumulates in the cytoplasm and therefore the cytoplasmic PKMYT1 kinase is ideally placed to modulate the latent pool of CDK1. Although experimentally increasing levels of cyclin B was sufficient to engender a vulnerability to PKMYT1 inhibition, it is likely that DNA replication stress and the upregulation of other modulators of CDK1 activity, such as CDC25 phosphatases, WEE1 activity, CAK kinase regulation or CDK inhibitors^22^, participate in imposing the need for PKMYT1-driven inhibitory phosphorylation. Furthermore, we cannot rule out that active cyclin E–CDK1 complexes are formed in *CCNE1*-amplified cells and that those drive cell cycle transitions as observed in *Cdk2*^*−/−*^ mice^42^.

The two-stage activation model of CDK1 regulation may also explain the noted pan-cellular cytotoxicity of WEE1 loss or inhibition. Inhibition of WEE1, but not that of PKMYT1, leads to CDK2 activation, which is also a consequence of *CCNE1* overexpression. The role of CDK2 in mediating WEE1 cytotoxicity has been demonstrated in multiple cell lines that do not harbour *CCNE1* amplification^40,43^. Therefore, we speculate that the reason why PKMYT1, but not WEE1, shows synthetic lethality with *CCNE1* amplification is owing to the selectivity of PKMYT1 for CDK1, which in turn makes PKMYT1 inhibition selective for conditions of *CCNE1* overexpression.

Finally, our work suggests avenues for drug combinations that may either drive more durable therapeutic responses or expand patient populations beyond *CCNE1* amplification. Indeed, we show that hydroxyurea or gemcitabine treatment enhances cyclin E-driven DNA replication stress leading to sensitization of cells and tumours to RP-6306. These observations also suggest that other agents that perturb DNA replication such as inhibitors of topoisomerase I, PARP, ATR or CHK1 may similarly display synergy with PKMYT1 inhibition. With respect to additional genetic alterations that could benefit from PKMYT1 inhibition, tumours with mutations in *FBXW7* (which encodes a substrate adaptor for the E3 ligase that targets cyclin E for ubiquitin-dependent proteolysis^44^) represents a target, given that cyclin E drives genome instability in these tumours^45^. Finally, we note that alterations in MMB–FOXM1-driven transcription are seen in multiple cancers^46,47^, including human papillomavirus-positive head and neck squamous cell carcinoma, where it causes sensitivity to WEE1 inhibition^48^. Therefore, determining whether MMB–FOXM1-driven transcription can be targeted following the loss of CDK1 inhibitory phosphorylation may expand the range of tumours that could benefit from PKMYT1 inhibitors.

## Methods

### Cell lines and cell culture

All cell lines were grown at 37 °C and 5% CO_2_. RPE1-hTERT *TP53*^*−/−*^ Cas9 (ref.^9^) and RPE1-hTERT *TP53*^*−/−*^ Cas9 *PKMYT1*^*−/−*^ cells were grown in DMEM (Life Technologies catalogue (cat.) no. 11965-092) with 10% FBS (Wisent cat. no. 080150) and 1% penicillin-streptomycin (Wisent cat. no. 450-201-EL). RPE1-hTERT Cas9 *TP53*^-/-^ *PKMYT1*^−/−^ cells were constructed by nucleofection of the parental cell line with *PKMYT1-7* sgRNA targeting exon 4 and single cell clones were generated by limiting dilution. Two clones were confirmed to be *PKMYT1*^−/−^ using western blot (clone J3.38 and J3.43). RPE1-hTERT *TP53*^*−/−*^ Cas9 *CCNE1*-high cell lines were constructed by piggyBac transposition of *CCNE1-2A-GFP* into the parental cell line and selection of clones with mid-range GFP expression. FT282-hTERT *TP53*^R^^*175H*^ wild-type (empty vector) and *CCNE1*-high cell lines were obtained from R. Drapkin^24^ and cultured in DMEM: F-12(1:1) (Life Technologies cat. no. 11330-032) with 5% FBS, 1% UltroserG (Pall Life Sciences cat. no.15950-017) and 1% penicillin-streptomycin. FT282-hTERT *TP53*^R^^*175H*^ *CCNE2*, *MYBL2* and *CCNB1* overexpressing cell lines were also constructed by piggyBac transposition of *CCNE2-2A-GFP, MYBL2-2A-GFP* or *CCNB1-2A-GFP* into the parental cell line and selection of clones with high GFP expression. FT282-hTERT *TP53*^R^^*175H*^ PCNA-cb-TagRFP expressing cell lines (wild-type and *CCNE1*-high) were transduced with PCNA-cb-TagRFP lentiviral particles and high RFP-expressing cells were selected. 293T cells (ATCC) were cultured in DMEM with 10% FBS and 1% penicillin-streptomycin. HEK293T cells (ATCC) were cultured in DMEM with 10% FBS and 1% penicillin-streptomycin. HCC1569 cells (ATCC) were cultured in RPMI 1640 (Life Technologies cat. no. 118575-093) with 10% FBS and 1% penicillin-streptomycin. SNU8 cells (KCLB) were cultured in RPMI 1640 with 10% FBS, 1% penicillin-streptomycin, 25 mM HEPES. OVCAR3 cells (ATCC) were cultured in RPMI 1640 with 20% FBS, 1% penicillin-streptomycin and 0.01 mg ml^−1^ insulin. A2780 cells (Sigma) were cultured in RPMI 1640 with 10% FBS and 1% penicillin-streptomycin. SUM149PT cells (Asterand Bioscience) were cultured in Ham’s F12 (Life Technologies cat. no. 11765-054) with 5% FBS, 10 mM HEPES, 1% penicillin-streptomycin, 1 μg ml^−1^ hydrocortisone and 5 μg ml^−1^ insulin. KYSE30 cells (DSMZ) were cultured in 45% RPMI 1640 with 45% Ham’s F12, 10% heat-inactivated FBS and 1% penicillin-streptomycin. TOV112D cells (ATCC) were cultured in 42.5% MCDB 105, 42.5% Medium 199 (Life Technologies cat. no. 11150-059), 15% FBS and 1% penicillin-streptomycin. NUGC3 cells (JCRB) were cultured in RPMI 1640 with 10% FBS and 1% penicillin-streptomycin. COV362 cells (Sigma) were cultured in DMEM with 10% FBS and 1% penicillin-streptomycin. DOTC24510 cells (ATCC) were cultured in DMEM with 10% FBS and 1% penicillin-streptomycin. None of the cell lines used were authenticated after reception. All cell lines used tested negative for mycoplasm contamination using MycoAlert. The OVCAR3 and HCC1569 cells have been shown to have amplified *CCNE1*^49,50^, whereas SNU8 has been shown to have *CCNE1* copy number gain (CCLE database (https://portals.broadinstitute.org/ccle)). SUM149PT cells are reported to have high cyclin E levels due to an *FBXW7* mutation^51^ but the clone we use does not display this cyclin E increase (Extended Data Fig. 9a).

### Plasmids

For CRISPR–Cas9 genome editing, sgRNAs were cloned either in lentiCRISPRv2 or in lentiguide NLS–GFP as described^52^. For PKMYT1 overexpression in cells, an N-terminally 3×Flag-tagged PKMYT1 open reading frame (CCDS10486.1) was cloned into the pDONR221 Gateway entry vector (Thermo Fisher Scientific, 12536017). Mutagenesis was performed by PCR to generate a PKMYT1 sgRNA-resistant version carrying silent mutations between nucleotides 966 and 981 (tgagttcactgccggt to cgaatttaccgctggc) and the kinase-dead mutant N238A. *PKMYT1* coding sequences were transferred by Gateway technology to the destination vector pCW57.1 (Addgene #41393) used for transduction in cells. For CDK1 mutant expression in cells the coding sequence for CDK1(T14A/Y15AF)–GFP was synthesized and cloned into the pHIV-NAT-hCD52 vector (a gift from R. Scully) using EcoRI and BamHI restriction enzymes. Mutagenesis was then performed to revert each phosphosite back to the wild type amino acids to create CDK1–GFP, CDK1(T14A)–GFP and CDK1(Y15F)–GFP. For time-lapse cell cycle microscopy, a PCNA-chromobody-TagRFP insert was amplified from pCCC-TagRFP (Chromotek) with EcoRI and BamHI restriction site sequence extensions and then cloned into pHIV-NAT-hCD52 vector. The sgRNA sequences used in this study are included in Supplementary Table 5.

### Lentiviral transduction

Lentiviral particles were produced in 293T cells in 10-cm plates by co-transfection of 10 μg of targeting vector with 3 μg VSV-G, 5 μg pMDLg/RRE and 2.5 μg pRSV-REV (Addgene #14888, #12251 and #12253) using calcium phosphate. Medium was refreshed 12–16 h later. Virus-containing supernatant was collected 36–40 h after transfection and cleared through a 0.2-μm filter. Viral transductions were performed in the presence of polybrene (Sigma-Aldrich, 4 μg ml^−1^ RPE1-hTERT *TP53*^*−/−*^ Cas9 and 16 μg ml^−1^ FT282-hTERT *TP53*^*R175H*^) at a multiplicity of infection (MOI) < 1.

### Antibodies

Primary antibodies used in this study include: histone H2A.X (phospho-S139, Cell Signalling Technologies cat. no. 2577, 1:500 for immunofluorescence), histone H2A.X (phospho-S139, Millipore Sigma cat. no. 05-636, 1:500 for immunofluorescence), CDK1 (Thermo Fisher Scientific cat. no. 33-1800, 1:1,000 for immunoblot and ELISA), CDK1-phosphoT14 (Abcam cat. no. ab58509, 1:1,000 for immunoblot and ELISA), CDK1-phoshoY15 (Cell Signaling Technology cat. no. 9111, 1:1,000 for immunoblot), PKMYT1 (Bethyl A302-424A, 1:1,000 for immunoblot), Histone H3-phosphoS10 (Cell Signaling Technology cat. no. 9706, 1:500 flow cytometry), lamin A/C (Cell Signaling Technology 4C11 cat. no. 4777, 1:500 for immunofluorescence), lamin A/C-phosphoS22 (Cell Signaling Technology D2B2E cat. no. 13448, 1:500 flow cytometry and for immunofluorescence), cyclin B1 (Cell Signalling Technologies cat. no. 2577, 1:500 for immunofluorescence, 1:1,000 for immunoblotting), α-tubulin (Millipore DM1A CP06, 1:4,000 for iimunoblotting), CDK2 (Upstate 05-596, 1:1,000 for immunoblotting), cyclin B1-phosphoS126 (Abcam ab55184, 1:500 for immunofluorescence), MCM2 (BD Biosciences 610700, 1:250 for immunofluorescence), MCM4 (Novus Biologicals H0004137-B01P, 1:500 for immunofluorescence), CHK1-phosphoS345 (Bethyl 2348, 1:1,000 for immunoblotting), cyclin E1 (Abcam ab3927, 1:1,000 for immunoblotting or Cell Marque cat. no. AC0120RUO 1:1,000 for immunohistochemistry), α-actinin (Millipore Sigma 05-384, 1:1,000 for immunoblotting), vinculin (Cell Signaling 13901S, 1:1,000 for immunoblotting), MYBL2 (Millipore MABE886, 1:1,000 for immunoblotting), MYBL2-pT487 (Abcam ab76009, 1:500 for immunoblotting). The following agarose-coupled antibodies were used for immunoprecipitation in kinase assays: CDK1 (Santa Cruz sc-54 AC) and CDK2 (Santa Cruz sc-6248 AC). The following secondary antibodies were used for immunoblotting: anti-mouse Irdye 800CW, anti-rabbit IRdye 680RD (926-32210 and 926-68071; LiCOR, 1:5,000), anti-mouse IgG–horseradish peroxidase (HRP) (Cedarlane cat. no. NA931-1ML, 1:4,000), anti-rabbit IgG–HRP (Cedarlane cat. no. 111-035-144, 1:4,000), anti-rabbit IgG–HRP (abcam 97051, 1:10,000). The secondary antibody used for ELISA was anti-rabbit IgG–HRP (Jackson Immunoresearch cat. no. 111-035-144). The following secondary antibodies were used for immunofluorescence and flow cytometry: AlexaFluor 488 donkey anti-rat IgG (Thermo Fisher Scientific A21208, 1:1,000), AlexaFluor 647 donkey anti-mouse IgG (Thermo Fisher Scientific A31571, 1:1,000), AlexaFluor 488 goat anti-mouse IgG (Thermo Fisher Scientific A11029, 1:1,000), AlexaFluor 647 goat anti-rabbit IgG (Thermo Fisher Scientific A21244, 1:1,000). Finally, the following secondary antibodies were used for AlphaLISA assays: AlphaLISA anti-rabbit IgG Acceptor beads (Perkin Elmer cat. no. AL104C) and AlphaLISA anti-mouse IgG Donor beads (Perkin Elmer cat. no. AS104D).

### Short interfering RNAs

Short interfering RNA (siRNA) oligonucleotides (siCTRL ON-TARGET Plus D-001210-03-50 and siCCNB1 ON-TARGET Plus L-003206-00-0005; Dharmacon) were transfected in Opti-MEM reduced-serum medium using Lipofectamine RNAiMAX agent (Thermo Fisher Scientific cat. no. 13778-075) following the manufacturer’s recommended protocol. Fresh medium was added to cells 16 h after transfection. Cells were used for high content imaging and immunoblotting 48 h after transfection.

### Fine chemicals

The following drugs were used in the course of the study: RP-6306 (this study), RP-6421 (this study) AZD1775 (Selleckchem, S1525), dinaciclib (MedChemExpress, HY-10492), PF-06873600 (MedChemExpress, HY-114177), RO-3306 (Selleckchem, S7747), gemcitabine (Cayman Chemicals, 9003096) and hydroxyurea (Sigma-Aldrich cat. no. H8627). Synthesis of RP-6306 and RP-6421 is described in the Supplementary Information. Concentration and duration of treatment is indicated in the legends of the corresponding figures.

### CRISPR screens

CCNE1-overexpression synthetic lethality screens were conducted as three parallel screens with a parental cell line and two isogenic clones overexpressing *CCNE1* (C2 and C21). For the screens, RPE1-hTERT Cas9 *TP53*^*−/−*^ parental and RPE1-hTERT Cas9 *TP53*^*−/−*^ *CCNE1*-overexpressing clones were transduced with the lentiviral TKOv2 sgRNA library at a low MOI (about 0.3) and medium containing 20 μg ml^−1^ puromycin (Life Technologies) was added the next day to select for transductants. The following day, cells were trypsinized and replated in the same plates while maintaining puromycin selection. Three days after infection, which was considered the initial time point (*t*_0_), cells were pooled together and divided into two sets of technical replicates. Cells were grown for a period of 18 d and cell pellets were collected every 3 d. Each screen was performed as a technical duplicate with a theoretical library coverage of ≥400 cells per sgRNA maintained at every step. Genomic DNA was isolated using the QIAamp Blood Maxi Kit (Qiagen) and genome-integrated sgRNA sequences were amplified by PCR using NEBNext Ultra II Q5 Master Mix (New England Biolabs). i5 and i7 multiplexing barcodes were added in a second round of PCR and final gel-purified products were sequenced on an Illumina NextSeq500 system at the LTRI NBCC facility (https://nbcc.lunenfeld.ca/) to determine sgRNA representation in each sample. Later, another screen was conducting using the next-generation TKOv3 library in RPE1-hTERT Cas9 *TP53*^*−/−*^ parental and RPE1-*CCNE1* (C2) cells using the same procedure outlined above.

The RP-6306 resistance screen was performed in two FT282-hTERT *TP53*^*R175H*^ *CCNE1-*high clones (C3 and C4) using TKOv3 sgRNA library at a MOI about 0.3. The screen was conducted in technical duplicates, and library coverage of >100 cells per sgRNA was maintained at every step. Puromycin-containing medium (2 µg ml^−1^) was added 2 days after infection to select for transductants. Selection was continued until 96 h after infection, which was considered the initial time point (*t*_0_). RP-6306 was added to the cells starting from time at day 6 (*t*_6_) at doses corresponding to individual LD80 (40 nM and 80 nM for clones C3 and C4, respectively). From *t*_10_ onwards, RP-6306 dose was adjusted to 60 nM for both clones and drug-containing medium was subsequently refreshed at *t*_12_, *t*_16_ and *t*_18_. The screen was terminated at *t*_21_. To identify genes whose deletion caused resistance to RP-6306, genomic DNA was isolated from surviving cells and processed as described above. Sample data analysis was performed using DrugZ algorithm previously described https://github.com/hart-lab/drugz.

### DepMap data mining

CRISPR dependency data^14,53^ (CERES scores) and gene-level copy number data^54^ were downloaded from the 2021 Q1 DepMap release using the Broad Institute’s DepMap portal. Cell lines were characterized as being ‘*CCNE1*-amplified’ if they had a copy number value that was greater than 1.58 (approximately equal to 2× total copy number relative to ploidy), or ‘WT’ if they had a copy number value that was less than or equal to 1.58; cell lines with no copy number data for *CCNE1* were removed from the analysis. From a total of 808 cell lines in the dependency dataset, 6 were removed, 20 were classified as *CCNE1*-amplified, and 782 were classified as WT. The Wilcoxon rank-sum test was used to compare dependency scores for each gene between the 2 groups. In Fig. 1b the difference in median gene depletion was plotted on the *x*-axis versus the nominal *P* value of the difference on the *y*-axis. Nominal *P* values are provided. Results of the analysis can be found in a tabular format in the source data.

### Clonogenic survival assays

Cells were seeded in 6-well plates, 300 cells per well for RPE1 and 400 for FT282. Single cells were grown out until distinct colonies formed with greater than 50 cells per colony. Colonies were rinsed with PBS and stained with 0.4% (w/v) crystal violet in 20% (v/v) methanol for 30 min. The stain was aspirated, and plates were rinsed twice in double-distilled H_2_O and air-dried. Colonies were counted using a GelCount instrument (Oxford Optronix, GelCount).

### Cell proliferation assays

RPE1-hTERT Cas9 *TP53*^*−/−*^, FT282-hTERT *TP53*^*R175H*^ and their respective *CCNE1*-high isogenic pairs were seeded in 96-well plates (Corning Costar cat. no. 5595) at a density of 150 cells per well for RPE1-hTERT Cas9 *TP53*^*−/−*^ *CCNE1* (C2) or 100 cells per well for all others. After 24 h, cells were treated using an automated D300e digital dispenser (Tecan) at drug concentrations ranging from 0.15 nM to 3 µM. Medium and drugs were refreshed every 3–4 days and cellular confluency was monitored up to 6 population doublings using an IncuCyte S3 Live-Cell Imager (Sartorius). Per cent confluence relative to a non-treated control was used to evaluate growth inhibition induced by test compounds. Synergy between RP-6306 and hydroxyurea or gemcitabine was analysed using the online SynergyFinder v2.0 tool^55^ using the ZIP model^56^ (https://synergyfinder.fimm.fi).

### Immunofluorescence

Cells were seeded onto glass coverslips and treated as indicated in the figure legends. Before collection, cells were pulsed with 20 μM EdU (5-ethynyl-2-deoxyuridine, Life Technologies cat. no. A10044) for 30 min and then washed with PBS and fixed with 4% paraformaldehyde (PFA) in PBS for 15 min at room temperature. Cells were then rinsed with PBS and permeabilized using 0.3% Triton X-100/PBS for 30 min. For chromatin-bound MCM measurements, cells were pre-extracted for 15 min on ice with CSK buffer (300 mM sucrose, 100 mM NaCl, 3 mM MgCl_2_, 10 mM PIPES pH 7.0, 0.5% v/v Triton-X 100) before PFA fixation. Cells were washed with PBS and incubated in blocking buffer (10% goat serum (Sigma cat. no. G6767), 0.5% NP-40 (Sigma-Aldrich, cat. no. I3021), 5% w/v saponin (Sigma-Aldrich, cat. no. 84510), diluted in PBS) for 30 min. Fresh blocking buffer containing primary antibodies was added for 2 h. Cells were rinsed three times with PBS and then blocking buffer, with secondary antibodies and 0.4 μg ml^−1^ DAPI (4,6-diamidino-2-phenylindole, Sigma-Aldrich, cat. no. D9542) was added for 1 h. After rinsing with PBS, immunocomplexes were fixed again using 4% PFA/PBS for 5 min. Cells were rinsed with PBS and incubated with EdU staining buffer (150 mM Tris-Cl pH 8.8, 1 mM CuSO_4_, 100 mM ascorbic acid and 10 μM AlexaFluor 555 azide (Life Technologies, cat. no. A20012) for 30 min. After rinsing with PBS coverslips were mounted onto glass slides with ProLong Gold mounting reagent (Invitrogen, cat. no. P36930). Images were acquired using a Zeiss LSM780 laser-scanning microscope (Oberkochen) with ZEN 2.3 SP1 software. Image analysis was performed using ImageJ v2.0.0.

### High content imaging and QIBC

For high-throughput analysis of nuclear γH2AX, 3,000 cells per well were seeded in 96-well plates and cultured for up to 72 h depending on the experiment. Cells were fixed, permeabilized and stained in the same manner as immunofluorescence described above. Wells were filled with 200 μl PBS and images were acquired at the Network Biology Collaborative Centre (LTRI) on an InCell Analyzer 6000 automated microscope (GE Life Sciences) with a 20× objective. Image analysis was performed using Cellprofiler 3.1.9 and RStudio v1.2.5019^57^ (Supplementary Fig. 4).

### Time-lapse microscopy

PCNA-cb-TagRFP expressing cells were maintained at 37 °C and 5% CO2 while deconvolution wide-field microscopy was performed using the DeltaVision Elite system equipped with an NA 0.75 20× UPlanSApo objective (Olympus) and an sCMOS 2,048 × 2,048 camera (Leica Microsystems). Each field was acquired every 10 min over 23 h with a *z*-step of 2 μm through the entire cell (7 sections) and deconvolved using softWoRx (v6.0, Leica Microsystems). Maximum intensity projections are shown (0.330 μm per pixel).

### Immunoblotting

Cell pellets were extracted by incubation in NP-40 lysis buffer (50 mM Tris-Cl pH 7.4, 250 mM NaCl, 5 mM EDTA, 1% NP-40, 0.02% NaN_3_, 1× protease inhibitor cocktail (Roche cat. no. 11836170001) for 30 min on ice. Extracts were cleared by centrifugation at 13,000*g* for 10 min at 4 °C. Cleared extracts were diluted in 2× sample buffer (20% glycerol, 2% SDS, 0.01% bromophenol blue, 167 mM Tris-Cl pH 6.8, 20 mM DTT) and boiled prior to separation by SDS–PAGE on Novex Tris–glycine gradient gels (Invitrogen, cat. no. XV0412PK20). Alternatively, cell pellets were boiled directly in 2× sample buffer before separation by SDS–PAGE. Proteins were transferred to nitrocellulose membranes (VWR, cat. no. CA10061-152), then blocked in 5% milk TBST and probed overnight with primary antibodies. Membranes were washed three times for five minutes with TBST, then probed with appropriate secondary antibodies for one hour, and washed again with TBST, three times for five minutes. Secondary antibody detection was achieved using an Odyssey Scanner (LiCOR) and analysed using Image Studio Lite v5.2.5 or enhanced chemiluminescence (ECL SuperSignal West Pico, Thermo Fisher Scientific cat. no. 34579).

### Flow cytometry

Cells were pulsed with 20 μM EdU (Life Technologies cat. no. A10044) for 30 min, collected by trypsinization, resuspended as single cells, washed once in PBS and pelleted at 600*g* for 3 min at 4 °C. All subsequent centrifugations were performed in this manner. Cells were fixed in 4% PFA/PBS for 15 min at room temperature, excess ice cold PBSB (1% BSA in PBS, 0.2 μM filtered) was added before pelleting. Cells were resuspended in permeabilization buffer (PBSB, 0.5% Triton-X 100) and incubated at room temperature for 15 min. Excess blocking buffer (PBSB, 0.1% NP-40) was added, cells were pelleted, resuspended in blocking buffer containing primary antibodies and incubated at room temperature for 1 h. Excess blocking buffer containing secondary antibodies was added, cells were pelleted, resuspended in blocking buffer and incubated at room temperature for 30 min. Excess blocking buffer was added, cells were pelleted and washed one additional time in PBSB. Cells were resuspended in EdU staining buffer (150 mM Tris-Cl pH 8.8, 1 mM CuSO_4_, 100 mM ascorbic acid and 10 μM AlexaFluor 555 azide (Life Technologies, cat. no. A20012)) and incubated at room temperature for 30 min. Excess PBSB was added, cells were pelleted and washed one additional time in PBSB. Cells were resuspended in analysis buffer (PBSB, 0.5 µg ml^−1^ DAPI, 250 µg µl^−1^ RNase A (Sigma-Aldrich, cat. no. R4875)) and incubated at 37 °C for 30 min or left at 4 °C overnight. Cells were analysed at the LTRI flow cytometry facility on a Fortessa X-20 (Becton Dickinson) using FACSDIVA v8.0.1 with at least 9,000 events collected and analysed using FlowJo v10.

### Immune complex histone H1 kinase assays

Cell pellets were resuspended in 250 μl EBN buffer (150 mM NaCl, 0.5% NP-40, 80 mM β-glycerol phosphate (Sigma-Aldrich, cat. no. 50020), 15 mM MgCl_2_, 20 mM EGTA, 1 mg ml^−1^ ovalbumin (Sigma-Aldrich, cat. no. 5503), 1× protease inhibitor cocktail (Roche, cat. no. 11836170001) pH 7.3) and incubated on ice for 5 min. Cell lysis was induced by two freeze–thaw cycles of incubation in liquid nitrogen and a 37 °C water bath, and lysates were cleared by centrifugation at 13,000*g* at 4 °C for 10 min. Protein concentration was determined by Bradford assay (Thermo Fisher Scientific cat. no. 1856209). For immunoprecipitation of kinases, 200 μg of extract was diluted in 750 μl EBN buffer and 10 μg of CDK1 or CDK2 primary antibody agarose bead conjugates were added to the extract and rotated at 4 °C overnight. Immunoprecipitates were pelleted by centrifugation at 2,500*g* at 4 °C for 5 min and washed 2× in 750 μl EBN followed by 1 ml EB (80 mM β-glycerol phosphate, 15 mM MgCl_2_, 20 mM EGTA, 1 mg ml^−1^ ovalbumin). After the final wash, the immunoprecipitates were resuspended in 500 μl EB and split into two samples. One sample was used for immunoblot analysis and the other used for kinase assays. Following removal of the final wash, immunoprecipitates were resuspended in 11 μl histone H1 kinase assay buffer (80 mM β-glycerol phosphate, 15 mM MgCl_2_, 20 mM EGTA, 1 mg ml^−1^ ovalbumin, 10 mM DTT, 0.15 μg μl^−1^ histone H1 (Sigma-Aldrich, cat. no. H1917), 22 μM ATP, 0.05 μCi μl^−1^ γ^32^P-ATP (Perkin Elmer NEG502A250UC), pH 7.3) and incubated at room temperature for 30 min. Reactions were quenched by addition of 5 μl 6× sample buffer (60% glycerol, 6% SDS, 0.03% bromophenol blue, 1,500 mM Tris-Cl pH 6.8, 60 mM DTT) and resolved by SDS–PAGE. Gels were exposed to a phosphor imaging screen for 1–2 d and imaged using a Typhoon FLA 9500 (GE Healthcare Life Sciences). 32P-H1 band intensity was quantified using ImageJ v2.0.0.

### Cytogenetic analyses

A total of 1.5 × 10^6^ FT282-hTERT *TP53*^*R175H*^ or HCC1569 cells was seeded in 10-cm dishes. Twenty-four hours later, RP-6306 was added at a final concentration of 500 nM for 24 h. KaryoMAX colcemid (100 ng ml^−1^) (Thermo Fisher Scientific cat. no. 15212-012) was added to the medium in the last 2 h of treatment and cells were collected as follows. Growth medium was stored in a conical tube. Cells were treated twice for 5 min with 1 ml trypsin. The growth medium and the 2 ml of trypsinization incubations were centrifuged (250*g*, 5 min, 4 °C). Cells were then washed with PBS and resuspended in 75 mM KCl for 15 min at 37 °C. Cells were centrifuged again, the supernatant was removed, and cells were fixed by dropwise addition of 1 ml fixative (ice-cold methanol:acetic acid, 3:1) with gentle vortexing. An additional 9 ml fixative was then added, and cells were fixed at 4 °C for at least 16 h. Once fixed, metaphases were dropped on glass slides and air-dried overnight. To visualize mitotic cells, slides were mounted in DAPI-containing ProLong Gold mounting medium (Invitrogen, cat. no. P36930). Images were captured on a Zeiss LSM780 laser-scanning confocal microscope with ZEN 2.3 SP1 software.

### MMB–FOXM1 transcriptional signature

Promoters of 114 protein-coding genes bound by both MYBL2 and FOXM1^58^ were used to create a MMB–FOXM1 transcriptional signature. To eliminate genes whose expression correlated poorly with other gene set members in TCGA samples the log_2_ fragments per kilobase of exon per million mapped fragments (FPKM) gene expression values were used to calculate pairwise Spearman correlations across the 11 genes in the signature. Genes with a mean correlation value below 0.4 were eliminated resulting in the 60 gene refined MMB–FOXM1 signature. The refined signature score for each TCGA sample was calculated by taking the median log_2_ FPKM value of all genes in the signature.

### RNA-seq sample preparation, sequencing and analysis

Cells were seeded in 10-cm dishes (2.5 × 10^6^ FT282-hTERT *TP53*^*R175H*^ wild type or 2 × 10^6^ *CCNE1-*high clone cells (C3 and C4)). The next day, cells were collected by trypsinization, washed once in PBS, and then pelleted. Pellets were snap-frozen in liquid nitrogen. RNA extraction and sequencing of the full transcriptome was performed using NovaSeq at BGI Hong Kong. Raw FASTQ files from a paired-end library were assessed using the FastQC v0.11.9 software (http://www.bioinformatics.babraham.ac.uk/projects/fastqc/) to determine the quality of the reads; read length was 150 bp. The FASTQ files were then aligned to the GENCODE GRCh38 v36 primary assembly of the human genome and quantified using Salmon v1.4.0^59^ with the command line flags “--validateMappings” and “--gcBias" to obtain read counts. Raw counts were processed using the bioconductor package edgeR v3.30.3 in R^60^. Genes expressed with counts per million (CPM) > 0.1 in at least two samples were considered and normalized using trimmed mean of M-values (TMM) to remove the library-specific artefacts. For subsequent analyses, voomY transformation was applied to RNA-seq count data to obtain normalized expression values on the log_2_ scale. Raw counts of sequencing reads with quality scores in FASTQ format and normalized transcript abundance measurements have been deposited in NCBI’s Gene Expression Omnibus^61^ and are accessible through GEO Series accession number GSE171453.

Heat maps were generated using the package heatmap3 v1.1.9 in R. Unsupervised hierarchical clustering was performed by calculating distances using the Pearson correlation metric and clustering using the complete method. Gene expression values were averaged and scaled across the row to indicate the number of standard deviations above (red) or below (blue) the mean, denoted as row *z*-score. GSEA^62^ was performed to identify the enrichment of genes co-regulated by MMB–FOXM1 in the FT282-hTERT *TP53*^*R175H*^ *CCNE1* C3 and C4 clones compared to parental wild-type cells. Analysis was performed using 1,000 permutations of the gene set, and normalized enrichment scores (NES) were obtained to reflect the degree to which the gene set is overrepresented in the FT282-hTERT *TP53*^*R175H*^ *CCNE1-*high C3 and C4 clones.

### ADP-Glo assay

For the ADP-Glo assay (Promega cat. no. V9103) human recombinant PKMYT1 (full-length human GST–PKMYT1 recombinant protein; Thermo Fisher Scientific A33387, lot 1938686), was diluted in enzyme assay buffer (70 mM HEPES, 3 mM MgCl_2_, 3 mM MnCl_2_, 50 μg ml^−1^ PEG20000, 3 μM sodium orthovanadate, 1.2 mM DTT) in a 5 μl volume and plated in 384-well plates (to a final concentration of 18.5 nM) followed by addition of 5 μl enzyme assay buffer. The enzyme–compound mix was incubated at room temperature for 15 min before addition of 5 μl of 30 μM ATP (diluted in enzyme assay buffer) so that the final ATP concentration was 10 μM. After incubation at 30 °C for 1 h, 15 μl of ADP-Glo reagent was added and incubated at room temperature for 40 min. Finally, 30 μl of the kinase detection reagent was added, the plate was incubated at room temperature for 30 min and luminescence was measured using an EnVision plate reader (Perkin-Elmer). Luminescence is measured for 0.25 s per well and rate per second was obtained by multiplying the luminescence value by 4.

### NanoBRET assay

To determine the affinity of RP-6306 in the PKMYT1 or WEE1 NanoBRET target engagement assay (Promega), HEK293T cells were transfected with a NanoLuc fusion vector for PKMYT1 (Promega NV1871) or WEE1 (Promega NV2231) with transfection carrier DNA (Promega E4881) using Fugene HD Transfection reagent (Promega E2311) in Opti-MEM without phenol red (Thermo Fisher Scientific, 11058021) and incubated overnight. Cells were trypsinized, counted and 17,000 cells per well were plated in 96-well plates with K-5 cell-permeable kinase NanoBRET TE tracer (Promega N2482) and RP-6306 and incubated for 2 h at 37 °C. Intracellular TE Nano-Glo Substrate/Inhibitor (Promega N2160) was added, and the intensity of the acceptor emission (610 nm) and the donor emission (450 nm) were measured using an EnVison plate reader (Perkin-Elmer).

### AlphaLISA assay

HCC1569 cells were plated into a 96-well TC-treated culture plate (30,000 cells per well) and grown overnight. The next day, RP-6306 was dispensed using a Tecan D300e digital dispenser with threefold dilutions. After compound addition, cell plates were centrifuged at 300*g* for 10 s, and then placed in the incubator for 2 h. Cells were lysed in AlphaLISA lysis buffer supplemented with 1× protease (Roche cat. no. 11836170001), and phosphatase inhibitors (Sigma-Aldrich cat. no. 4906837001) and 1 mM PMSF. Plates were rotated at 500*g* for 20 min to facilitate lysis. Plates were then sealed with aluminium foil and frozen at −80 °C for at least 1 h. Lysates were thawed at 37 °C for 10 min and 10 μl of each lysate was transferred in duplicate to 384 well assay plates. Antibodies were added at a final concentration of 5 nM or 1 nM for CDK1-pT14 and total CDK1 or CDK1-pY15 and total CDK1, respectively, sealed and stored at 4 °C overnight. Anti-rabbit IgG Acceptor and anti-mouse IgG donor beads were each added at a final concentration of 20 μg ml^−1^ and the reactions were incubated in the dark for 2 h at room temperature. Luminescence was measured using a Perkin Elmer EnVision Multimode plate reader with excitation at 680 nm and emission at 615 nm.

### Animal studies

Mice were housed and experiments were performed at Repare Therapeutics (NEOMED site, Montreal, Canada), which is a Canadian Council on Animal Care (CCAC)-accredited vivarium. Studies were conducted under a protocol approved by the NEOMED Institutional Animal Care Committee (NIACC). Mice were inspected upon arrival and group-housed (3–5 per cage) in individual HEPA ventilated autoclaved cages (Innocage IVC, Innovive) in a temperature-controlled environment (22 ± 1.5 °C, 30–80 % relative humidity, 12 h:12 h light:dark). Mice were provided with autoclaved corncob bedding, irradiated food (Harlan Teklad) and filtered water ad libitum. They were also provided with nesting material (Ketchum cat. no. 087) and a plastic shelter as enrichment. Fresh bedding, nesting material and water was replenished and replaced on a weekly basis. Mice were acclimatized in the animal facility for at least 5 days prior to use and were identified with indelible ink. Experiments were performed during the light phase of the cycle.

### Cell line-derived and patient-derived xenografts

HCC1569, OVCAR3 and SUM149PT cells were implanted at 5 × 10^6^ cells per mouse into the right flanks of female CB17-SCID, SCID-beige and NOD-SCID mice respectively (5–7 weeks old; Charles River), in 1:1 Matrigel:medium (Matrigel Corning, cat. no. CB35248). When tumours reached the target size of 100–150 mm^3^, mice (*n* = 8) were randomized to treatment groups according to tumour volume and body weight using the ‘stratified’ method in Studylogv4.4 software and treatment with RP-6306 was initiated.

In vivo studies using PDX were conducted at Crown Biosciences. Fresh primary human tumour tissue was collected and cut into small pieces (approximately 2–3 mm in diameter). These tumour fragments were inoculated subcutaneously into the right flank of female BALB/c nude mice (5–7 weeks old) for tumour development and subsequently passaged by implantation into the cohort of mice enrolled in the efficacy study. Mice were randomized according to growth rate into treatment groups (*n* = 6) when the mean tumour size reached approximately 150 (100–200) mm^3^ using the stratified method in Studylogv4.4 software. The procedures involving the care and use of animals in for the PDX model were reviewed and approved by the Institutional Animal Care and Use Committee (IACUC) of CrownBio prior to execution. During the study, the care and use of animals were conducted in accordance with the regulations of the Association for Assessment and Accreditation of Laboratory Animal Care (AAALAC).

RP-6306 was formulated in 0.5% methylcellulose and orally administered twice daily (BID, 0–8 h) for a maximum of 21 days. Gemcitabine was administered once weekly intraperitoneally in saline. Animals were monitored for tumour volume, clinical signs and body weight three times per week. Tumour volume was measured using a digital calliper and calculated using the formula 0.52 × *L* × *W*^2^, where *L* is length and *W* is width. Response to treatment was evaluated for tumour growth inhibition (% TGI). Tumour growth inhibition (TGI) was defined as: TGI = ((TV_vehicle/last_ − TV_vehicle/day0_) − (TV_treated/last_ − TV_treated/day0_))/(TV_vehicle/last_ − TV_vehicle/day0_) × 100% calculated based on the means of the treatment groups at day 0 and last day of measurement. TV is tumour volume and subscripts indicate treatment group and time of sampling. According to NIACC and IACUC approved animal protocols, mice were euthanized as soon as their tumour volume exceeded 2,000 mm^3^. Change in body weight (BW) was calculated using the formula: %BW change = (BW_last_ − BW_day0_/BW_day0_) × 100. BW change was calculated based on individual body weight changes relative to day 0. Statistical significance relative to vehicle control or other test groups was established by one-way ANOVA followed by Fisher’s least significant difference test for multiple groups and unpaired *t*-test for two group comparisons (GraphPad Prism v9.0). Investigators were not blinded during data collection and analysis.

### Blood and tumour tissue collection

Under isoflurane anaesthesia, whole blood was collected by cardiac puncture and transferred to tubes containing 0.1 M citric acid (3:1 citric acid:blood) and stored at −20 °C for LC–MS/MS analysis. Tumours were removed from mice flanks and cleared of surrounding mouse stroma. Tumour pieces between 50 mg and 100 mg were collected in a pre-weighed pre-filled bead mill tube (Fisher Scientific, cat. no. 15-340-154) and then flash-frozen in liquid nitrogen. Other tumour fragments from vehicle- and compound-treated mice were placed in 10% neutral buffered formalin (NBF) within 2–5 min of surgical excision, fixed in NBF for 18–24 h at room temperature and embedded in paraffin.

### RP-6306 quantification by LC–MS/MS

The extraction of whole blood samples was performed by protein precipitation using four volumes of acetonitrile. The sample extracts were analysed using a Transcend LX2 Ultimate 3000 liquid chromatography system coupled to a Thermo Altis triple quadrupole electrospray mass spectrometer (Thermo Fisher Scientific) operated in positive mode. Separations were performed using a 2 × 50 mm, 2.8 µm Pursuit XRS C8 HPLC column (Agilent). A reversed-phase linear gradient of water + 0.1% formic acid and 1:1 acetonitrile:MeOH was used to elute RP-6306 and the internal standard. Samples were quantified against a ten-point linear standard curve and three levels of quality control samples. Whole blood concentrations of RP-6306 were converted to free unbound plasma concentrations using an in vitro derived blood/plasma ratio = 1.2 and fraction unbound (*f*_u_) plasma = 0.185 from the CD-1 mouse strain.

### Immunohistochemistry

Histology in Extended Data Fig. 9 was performed by HistoWiz. In brief, the formalin-fixed tissues were dehydrated through a 20%, 80%, 95% and 100 % ethanol series, cleaned in xylene, embedded in paraffin then sectioned at 4 μm. Immunohistochemistry for γH2AX, CDK1-pT14 and cyclin B1-pS126 were performed on a Bond Rx autostainer (Leica Biosystems) with heat antigen retrieval. Bond polymer refine detection (Leica Biosystems) was used according to manufacturer’s protocol. After staining, sections were dehydrated and film coverslipped using a TissueTek-Prisma and Coverslipper (Sakura). Whole-slide scanning (40×) was performed on an Aperio AT2 (Leica Biosystems). Image quantification analysis was performed using HALO. H-score is given by the formula: H-score = (1 × percentage of weakly positive cells) + (2 × percentage of moderately positive cells) + (3 × percentage of strongly positive cells). Histology in Extended Data Fig. 10c was performed by NeoGenomics. In brief, formalin-fixed, paraffin-embedded tumour samples were sectioned at 4 μm, mounted on charged glass slides and baked at 60 °C for 1 h. Immunohistochemistry for cyclin E1 was performed on a Bond-III autostainer (Leica Biosystems). Bond polymer refine detection (Leica Biosystems) was used according to the manufacturer’s protocol. Slides were then removed from the instrument dehydrated, cleared and coverslipped. Bright-field images (20×) were acquired on an Aperio AT2 (Leica Biosystems).

### ELISA

Tumour samples were homogenized in MSD Tris lysis buffer (Meso Scale Discovery cat. no. R60TX-2) supplemented with 1× Halt Protease (Thermo Fisher Scientific cat. no. 78429) and phosphatase inhibitors (Thermo Fisher Scientific cat. no. 78426) using a Beadruptor tissue homogenizer (OMNI International. After homogenization, samples were centrifuged at 14,000*g* for 5 min at 4 °C. ELISA plates were coated with the capture antibody (CDK1) incubated overnight at 4 °C, washed and then blocked for 1 h at room temperature. Tissue samples (60 μg) were added to the plates to incubate for 2.5 h at room temperature. After washing, the detector antibody (CDK1-pT14) was added for 1 h at room temperature. After washing and plate drying, detection occurred using a secondary anti-rabbit HRP conjugate incubation for 1 h followed by a 10-min incubation with TMB peroxidase substrate stop solution (Thermo Fisher Scientific cat. no. N600). The absorbance was measured in 96-well plate format on an EnVision2105 at 450 nm. Samples were quantified relative to a standard protein extract and an MSD lysis buffer used as a blank to control for inter-day variability.

### Reporting summary

Further information on research design is available in the Nature Research Reporting Summary linked to this paper.

## Online content

Any methods, additional references, Nature Research reporting summaries, source data, extended data, supplementary information, acknowledgements, peer review information; details of author contributions and competing interests; and statements of data and code availability are available at 10.1038/s41586-022-04638-9.

## Supplementary information

Supplementary Information Methods. Synthesis and characterization of RP-6306 and RP-6421; Supplementary Figure 1. Immunoblot source data; Supplementary Figure 2. FACS gating strategy; Supplementary Figure 3. Model of synthetic lethal relationship between *CCNE1*-amplification and PKMYT1 inhibition; Figure 4. QIBC workflow; Supplementary Table 2. Summary of TIDE editing performed in the course of this study; Supplementary Table 3. Pharmacokinetic parameters of RP-6306 in mice; Supplementary Table 4. MMB–FOXM1 transcriptional signature; Supplementary Table 5. sgRNA guide sequences.

Reporting Summary

Supplementary Table 1 Read counts for CRISPR screens conducted in this study. Tab 1 contains read counts for CCNE1 synthetic lethal screens in parental and *CCNE1* (C2 and C21) RPE1-hTERT *TP53*^*−/−*^ using the TKOv2 library. Tab2 contains read counts for CCNE1 synthetic lethal screens in parental and *CCNE1* (C2) RPE1-hTERT *TP53*^*−/−*^ using the TKOv3 library. Tab 3 contains read counts for RP-6306 resistance screen in parental and *CCNE1* (C3 and C4) FT282-hTERT *TP53*^*R175H*^ using the TKOv3 library. See main text and methods for further details.

Supplementary Video 1 Time-lapse microscopy of WT FT282-hTERT *TP53*^*R175H*^ cells expressing PCNA-cb-TagRFP. Cells were imaged every 10 min for 23 h. Following live-cell imaging cells were fixed, probed with antibodies against γH2AX and re-imaged at the same coordinates. The fixed  γH2AX image is appended as the final frame of the movie.

Supplementary Video 2 Time-lapse microscopy of *CCNE1* (C3) FT282-hTERT *TP53*^*R175H*^ cells expressing PCNA-cb-TagRFP. Cells were imaged every 10 min for 23 h. Following live-cell imaging cells were fixed, probed with antibodies against γH2AX and re-imaged at the same coordinates. The fixed γH2AX image is appended as the final frame of the movie.

Supplementary Video 3 Time-lapse microscopy of WT FT282-hTERT *TP53*^*R175H*^ cells expressing PCNA-cb-TagRFP treated with 500 nM RP-6306. Cells were imaged immediately after RP-6306 addition every 10 min for 23 h. Following live-cell imaging cells were fixed, probed with antibodies against γH2AX and re-imaged at the same coordinates. The fixed γH2AX image is appended as the final frame of the movie.

Supplementary Video 4 Time-lapse microscopy of *CCNE1* (C3) FT282-hTERT *TP53*^*R175H*^ cells expressing PCNA-cb-TagRFP treated with 500 nM RP-6306. Cells were imaged immediately after RP-6306 addition every 10 min for 23 h. Following live-cell imaging cells were fixed, probed with antibodies against γH2AX and re-imaged at the same coordinates. The fixed γH2AX image is appended as the final frame of the movie.

## Data Availability

CRISPR screen raw counts of sequencing reads with quality scores in FASTQ format have been deposited in the NCBI Sequence Read Archive and are accessible through BioProject accession number PRJNA808613. Read counts of the CRISPR screens are available in Supplementary Table 1. RNA-seq raw counts of sequencing reads with quality scores in FASTQ format and normalized transcript abundance measurements have been deposited in NCBI’s Gene Expression Omnibus and are accessible under accession number GSE171453. All data supporting the findings of this study are available from the corresponding authors on reasonable request. Source data are provided with this paper.

## Source data

Source Data Fig. 1
Source Data Fig. 2
Source Data Fig. 3
Source Data Fig. 4
Source Data Fig. 5
Source Data Extended Data Fig. 1
Source Data Extended Data Fig. 2
Source Data Extended Data Fig. 3
Source Data Extended Data Fig. 4
Source Data Extended Data Fig. 5
Source Data Extended Data Fig. 6
Source Data Extended Data Fig. 7
Source Data Extended Data Fig. 8
Source Data Extended Data Fig. 9
Source Data Extended Data Fig. 10
