# Supplementary material for: CCNE1 amplification is synthetic lethal with PKMYT1 kinase inhibition
Source: Nature. 2022 Apr 20;604(7907):749–56. doi: 10.1038/s41586-022-04638-9 (PMC9046089; doi:10.1038/s41586-022-04638-9)
Supplement: Supplementary file 1 — Methods. Synthesis and characterization of RP-6306 and RP-6421; Supplementary Figure 1. Immunoblot source data; Supplementary Figure 2. FACS gating strategy; Supplementary Figure 3. Model of synthetic lethal relationship between CCNE1-amplification and PKMYT1 inhibition; Figure 4. QIBC workflow; Supplementary Table 2. Summary of TIDE editing performed in the course of this study; Supplementary Table 3. Pharmacokinetic parameters of RP-6306 in mice; Supplementary Table 4. MMB–FOXM1 transcriptional signature; Supplementary Table 5. sgRNA guide sequences. [file 41586_2022_4638_MOESM1_ESM.pdf]

nature portfolio

https://doi.org/10.1038/s41586-022-04638-9

Supplementary information

CCNE1 amplification is synthetic lethal with PKMYT1 kinase inhibition

In the format provided by the authors and unedited

Methods:

Synthesis of RP-6306

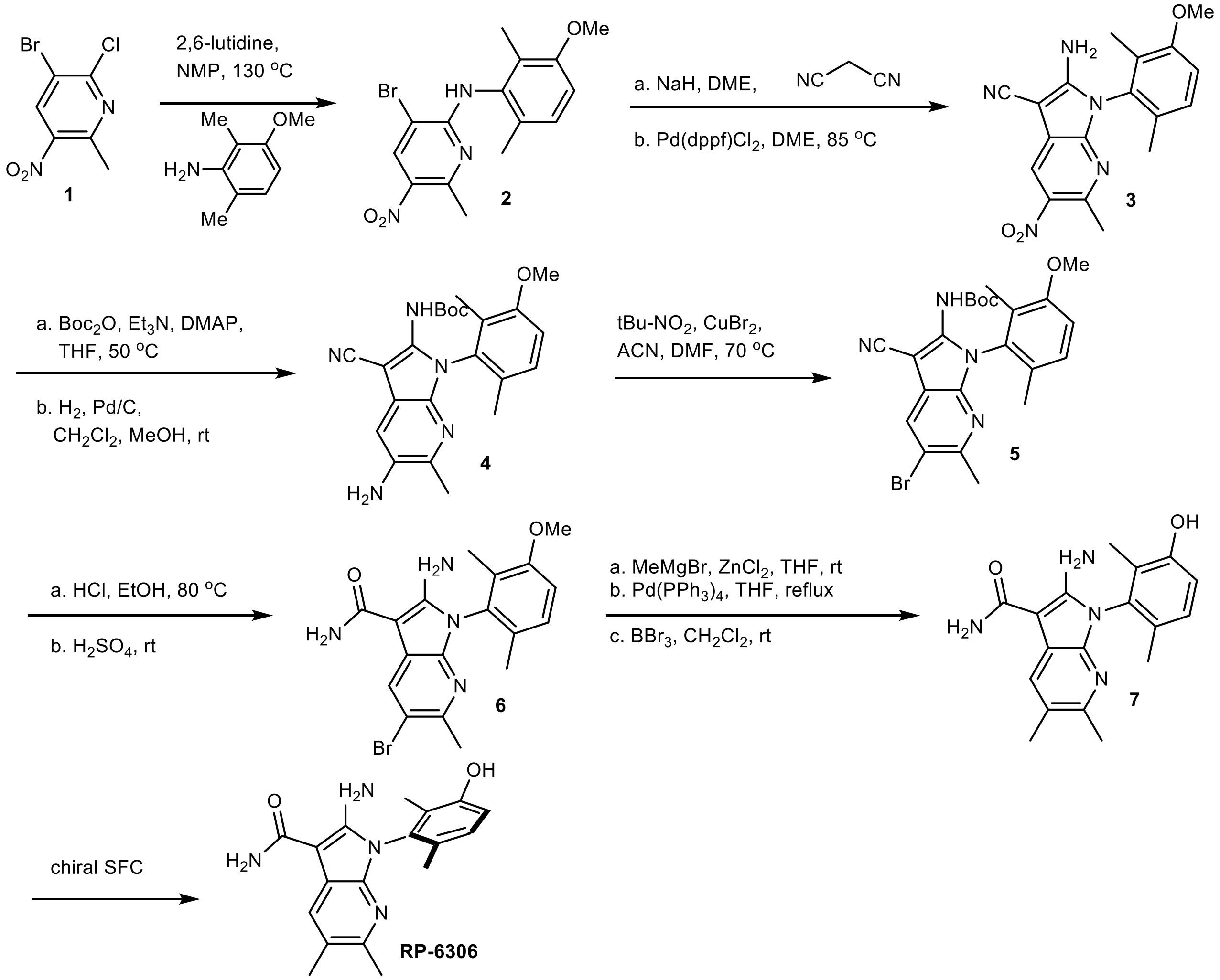

3-bromo-N-(3-methoxy-2,6-dimethyl-phenyl)-6-methyl-5-nitro-pyridin-2-amine (**2**): A pressure vessel was loaded with 3-bromo-2-chloro-6-methyl-5-nitro-pyridine (10.11 g, 40.2 mmol, Combi-blocks OS-2467) and 3-methoxy-2,6-dimethylaniline (9.20 g, 60.8 mmol, Majetich, George; Yu, Jianhua, Canadian Journal of Chemistry, 2012, vol. 90, # 1, p. 75 - 84). NMP (40 mL) and 2,6-dimethylpyridine (8.58 g, 80.1 mmol, 9.3 mL) were added and the reaction mixture was heated to 130 °C (pellet bath) for 5 days until satisfactory conversion as assessed by UPLCMS was achieved. The reaction mixture was cooled to RT and the resulting paste transferred to a conical flask and 500 mL HCl 0.5 N was added dropwise while stirring, resulting in a sticky gum. The supernatant was filtered on a Buchner funnel. The remaining gum was washed with H_2O, dissolved in DCM, and combined with the solid which had also been dissolved in DCM (200 mL total). The DCM

solution was dried over Na_2SO_4, filtered and concentrated. The crude residue was purified by silica gel chromatography (dry load) eluting with a gradient of 0 to 100% DCM in heptanes to provide 3-bromo-N-(3-methoxy-2,6-dimethyl-phenyl)-6-methyl-5-nitro-pyridin-2-amine (**2**, 10.5 g, 71% yield) as a light yellow solid. *m/z* (ESI, +ve ion): 368.0 $(M+H)^+$.

<u>2-amino-1-(3-methoxy-2,6-dimethyl-phenyl)-6-methyl-5-nitro-pyrrolo[2,3-b]pyridine-3-carbonitrile (**3**):</u> To a RBF containing sodium hydride (3.13 g, 72.2 mmol, 60% w/w in mineral oil) in DME (150 mL) was added a solution of propanedinitrile (4.75 g, 71.9 mmol) in DME (50 mL) slowly. After stirring for 1h, compound **2** (10.5 g, 28.7 mmol) and $Pd(dppf)Cl_2$•DCM (2.31 g, 2.83 mmol) were added. The resulting mixture was degassed by bubbling N_2 through solution, equipped with a condenser, and heated to reflux for 1h. The reaction mixture was cooled to RT, poured into saturated aqueous NH_4Cl and extracted with DCM (3x). The combined organic extracts were washed with H_2O, brine, dried over Na_2SO_4, filtered and adsorbed on silica. The crude residue was purified by silica gel chromatography (dry load) eluting with a gradient of 0 to 100% EtOAc in heptanes. Appropriate fractions were combined, concentrated and the resulting solid was triturated with DCM, filtered, and dried in vacuo, affording 2-amino-1-(3-methoxy-2,6-dimethyl-phenyl)-6-methyl-5-nitro-pyrrolo[2,3-b]pyridine-3-carbonitrile (7.97 g, 79% yield) as a bright yellow solid. A second crop of material was obtained from the filtrate from the previous trituration by purification by flash chromatography and trituration in the same fashion, providing additional 2-amino-1-(3-methoxy-2,6-dimethyl-phenyl)-6-methyl-5-nitro-pyrrolo[2,3-b]pyridine-3-carbonitrile (**3**, 1.04 g, 10% yield) as a dark yellow solid. *m/z* (ESI, +ve ion): 352.1 $(M+H)^+$.

<u>tert-butyl N-[5-amino-3-cyano-1-(3-methoxy-2,6-dimethyl-phenyl)-6-methyl-pyrrolo[2,3-b]pyridin-2-yl]carbamate (**4**)</u>: To a solution of compound **3** (9.0 g, 25.6 mmol) in THF (120 mL) was added triethylamine (7.99 g, 78.9 mmol, 11 mL) , DMAP (312 mg, 2.55 mmol) and tert-butoxycarbonyl tert-butyl carbonate (17.0 g, 77.9 mmol) . The mixture was stirred at 50 °C for 40 min. The heating was stopped, and ethylenediamine (6.20 g, 103 mmol, 6.90 mL) was added and the mixture was stirred at RT for 45 min, then diluted with H_2O and DCM. The layers were separated and the aqueous layer was extracted with DCM (2x). The combined organic extracts were washed with half-saturated brine, dried over Na_2SO_4, filtered and concentrated. The crude residue was purified by silica gel chromatography eluting with a gradient of 0 to 60% EtOAc in

heptanes to provide crude tert-butyl N-[3-cyano-1-(3-methoxy-2,6-dimethyl-phenyl)-6-methyl-5-nitro-pyrrolo[2,3-b]pyridin-2-yl]carbamate (13.94 g) as an off-white solid, which was contaminated with tert-butyl N-[2-(tert-butoxycarbonylamino)ethyl]carbamate (50 mol% by ^{1}H NMR). ^{1}H NMR (400 MHz, DMSO-d6) δ 10.16 (s, 1H), 8.78 (s, 1H), 7.26 (d, *J* = 8.5 Hz, 1H), 7.13 (d, *J* = 8.5 Hz, 1H), 3.85 (s, 3H), 2.66 (s, 3H), 1.77 (s, 3H), 1.66 (s, 3H), 1.40 (s, 9H). *m/z* (ESI, +ve ion): 452.1 $(M+H)^+$. To a RBF containing crude tert-butyl N-[3-cyano-1-(3-methoxy-2,6-dimethyl-phenyl)-6-methyl-5-nitro-pyrrolo[2,3-b]pyridin-2-yl]carbamate (13.94 g, 25.6 mmol) in DCM (280 mL) and MeOH (280 mL) was added palladium on carbon (2.08 g, 1.95 mmol, 10%w/w) as a slurry in some of the solvent mixture. The reaction mixture was flushed with H_2 and stirred under H_2 atmosphere (balloon) overnight. The reaction mixture was flushed with N_2, filtered on a celite pad, rinsed with DCM and MeOH. The filtrate was concentrated and dried in vacuo, affording a light yellow solid which was purified by silica gel chromatography (dry load) eluting with a gradient of EtOAc (20 to 100%) in heptanes. Appropriate fractions were combined and concentrated in vacuo to afford tert-butyl N-[5-amino-3-cyano-1-(3-methoxy-2,6-dimethyl-phenyl)-6-methyl-pyrrolo[2,3-b]pyridin-2-yl]carbamate (**4**, 9.34 g, 87% yield) as an off-white solid. ^{1}H NMR (400 MHz, DMSO-d6) δ 9.50 (s, 1H), 7.25 – 7.14 (m, 2H), 7.05 (d, *J* = 8.4 Hz, 1H), 4.95 (s, 2H), 3.82 (s, 3H), 2.21 (s, 3H), 1.75 (s, 3H), 1.63 (s, 3H), 1.35 (s, 9H). *m/z* (ESI, +ve ion): 422.2 $(M+H)^+$.

tert-butyl N-[5-bromo-3-cyano-1-(3-methoxy-2,6-dimethyl-phenyl)-6-methyl-pyrrolo[2,3-b]pyridin-2-yl]carbamate (**5**): To a solution of compound **4** (10.34 g, 24.5 mmol) in acetonitrile (100 mL) and DMF (60 mL) was added tert-butyl nitrite (5.20 g, 50.5 mmol, 6.0 mL), followed by copper(II) bromide (6.58 g, 29.4 mmol). The mixture was heated to 70 °C for 35 min, cooled to RT, diluted with H_2O (600 mL) and NH_4OH conc (30 mL) and extracted with EtOAc (3x). The combined organic extracts were washed with saturated NH4Cl (2x), half-saturated brine, dried over Na_2SO_4, filtered and concentrated. The residue was purified by silica gel chromatography (dry load) eluting with a gradient of EtOAc (0 to 100%) in heptanes. Appropriate fractions were combined and concentrated in vacuo to afford tert-butyl N-[5-bromo-3-cyano-1-(3-methoxy-2,6-dimethyl-phenyl)-6-methyl-pyrrolo[2,3-b]pyridin-2-yl]carbamate (**5**, 6.18 g, 52% yield) as an ivory solid. m/z (ESI, +ve ion): 485.1 $(M+H)^+$.

<u>2-amino-5-bromo-1-(3-methoxy-2,6-dimethyl-phenyl)-6-methyl-pyrrolo[2,3-b]pyridine-3-carboxamide (**6**):</u> Compound **5** (6.18 g, 12.7 mmol) in EtOH (60 mL) was treated with aqueous HCl (6M, 34 mL) and stirred for 70 min at 80 °C then cooled to RT and concentrated. The residue was dissolved in MeOH, made alkaline with excess Et_3N and concentrated again. The residue was purified by silica gel chromatography (dry load) eluting with a gradient of EtOAc (0 to 100%) in heptanes. Appropriate fractions were combined and concentrated in vacuo to afford 2-amino-5-bromo-1-(3-methoxy-2,6-dimethyl-phenyl)-6-methyl-pyrrolo[2,3-b]pyridine-3-carbonitrile (3.70 g, 75% yield) as a dark magenta solid. *m/z* (ESI, +ve ion): 385.1 $(M+H)^+$. 2-Amino-5-bromo-1-(3-methoxy-2,6-dimethyl-phenyl)-6-methyl-pyrrolo[2,3-b]pyridine-3-carbonitrile (3.70 g, 9.6 mmol) was stirred in concentrated sulfuric acid (18M, 25 mL) for 55 min, then the reaction mixture was quenched with crushed ice, placed in an ice bath and made alkaline to pH 8-9 with saturated NH_4OH added slowly. The resulting solid was collected by filtration on a Buchner funnel and washed with H_2O. The material was air-dried then co-evaporated twice with toluene and dried in vacuo, then stirred in 10% MeOH/DCM and filtered on a silica plug, eluting with 10% MeOH/DCM to remove residual ammonium salts. The filtrate was concentrated then dried in vacuo, affording 2-amino-5-bromo-1-(3-methoxy-2,6-dimethyl-phenyl)-6-methyl-pyrrolo[2,3-b]pyridine-3-carboxamide (**6**, 3.80 g, 98% yield) as a pink solid. *m/z* (ESI, +ve ion): 403.0 $(M+H)^+$.

<u>2-amino-1-(3-hydroxy-2,6-dimethyl-phenyl)-5,6-dimethyl-pyrrolo[2,3-b]pyridine-3-carboxamide (7):</u> To a solution of methyl magnesium chloride (3M, 18.8 mL) in THF (160 mL) in a RBF under N_2 was added a solution of zinc dichloride in THF (0.5M, 112 mL) at RT dropwise via an addition funnel. After the addition, the resulting white suspension was stirred at RT for 35 min. To the zincate solution was added compound **6** (4.48 g, 11.1 mmol), the flask was rinsed with 20 mL THF, and palladium (0) tetrakis(triphenylphosphine) (1.14 g, 0.987 mmol) was added. The mixture was bubbled through with N_2 then equipped with a condenser and refluxed (heat block set to 80 °C) for 24h. The reaction mixture was cooled to RT, then diluted with saturated aqueous NH_4Cl and extracted with EtOAc (3x). The combined organic extracts were washed with brine, dried over Na_2SO_4, filtered and concentrated. The residue was purified by silica gel chromatography (dry load) eluting with a gradient of EtOAc (0 to 100%) in heptanes then purified again by silica gel chromatography (dry load) eluting with a gradient of MeOH (1 to 15%) in DCM. Appropriate fractions from the two columns were combined and concentrated in vacuo to

afford 2-amino-1-(3-methoxy-2,6-dimethyl-phenyl)-5,6-dimethyl-pyrrolo[2,3-b]pyridine-3-carboxamide (2.22 g, 59% yield, 77% purity) as a light pink solid, which contained some 2-amino-1-(3-methoxy-2,6-dimethyl-phenyl)-6-methyl-pyrrolo[2,3-b]pyridine-3-carboxamide side product (19% by UPLCMS). *m/z* (ESI, +ve ion): 339.2 $(M+H)^+$. To a suspension of 2-amino-1-(3-methoxy-2,6-dimethyl-phenyl)-5,6-dimethyl-pyrrolo[2,3-b]pyridine-3-carboxamide (2.22 g, 6.56 mmol, 77% purity) in DCM (25 mL) was added tribromoborane in DCM (1M, 26 mmol, 26 mL) dropwise. The reaction mixture was stirred at RT for 45 min, then concentrated to dryness. The crude product was taken in DCM and placed in an ice bath and MeOH was added carefully (exotherm). The mixture was concentrated to dryness then co-evaporated twice with MeOH. The residue was triturated with saturated aqueous $NaHCO_3$. The solids were collected by filtration on a Buchner funnel, washed with H_2O and air-dried. The still wet solid was dissolved in DCM/MeOH, concentrated to dryness and triturated in 20% MeOH/DCM (50 mL). The solid was collected by filtration, washed with 20% MeOH/DCM, air-dried then dried in vacuo to afford 2-amino-1-(3-hydroxy-2,6-dimethyl-phenyl)-5,6-dimethyl-pyrrolo[2,3-b]pyridine-3-carboxamide (1.60g, 75% yield) as a light beige solid. MS: [M+1]: 325.1. A different batch was purified by preparative HPLC to yield 2-amino-1-(3-hydroxy-2,6-dimethyl-phenyl)-5,6-dimethyl-pyrrolo[2,3-b]pyridine-3-carboxamide (**7**, 63% yield) as an off-white fluffy solid. ^{1}H NMR (400 MHz, DMSO-d6) δ 9.51 (s, 1H), 7.82 (s, 1H), 7.05 (d, *J* = 8.3 Hz, 1H), 6.90 (d, *J* = 8.2 Hz, 1H), 6.71 (br s, 2H), 6.64 (br s, 2H), 2.26 (s, 3H), 2.23 (s, 3H), 1.74 (s, 3H), 1.65 (s, 3H). *m/z* (ESI, +ve ion): 325.1 $(M+H)^+$.

Chiral SFC separation of **7** (1.60g, 4.93 mmol) (Instrument: Waters Prep 100 SFC-MS; Column: Phenomenex Lux Cellulose-2, 30 x 250 mm, 5 µm; Conditions: isocratic at 55% IPA + 10mM Ammonium Formate with 45% CO2; Flow Rate: 70 mL/min) provided **RP-6306**. Peak 1 (retention time 3.94 min, 99.86%): (S)-2-amino-1-(3-hydroxy-2,6-dimethyl-phenyl)-5,6-dimethyl-pyrrolo[2,3-b]pyridine-3-carboxamide (**RP-6306**, 381 mg) was obtained as an off white fluffy solid. ^{1}H NMR (400 MHz, DMSO-d6) δ 9.49 (s, 1H), 7.84 (s, 1H), 7.06 (d, *J* = 8.0 Hz, 1H), 6.92 (d, *J* = 8.0 Hz, 1H), 6.72 (bs, 2H), 6.66 (bs, 2H), 2.27 (s, 3H), 2.25 (s, 3H), 1.76 (s, 3H), 1.68 (s, 3H). ^{13}C NMR (101 MHz, DMSO) δ 168.90, 154.62, 152.27, 145.59, 143.95, 133.10, 128.26, 127.51, 125.66, 124.47, 124.11, 116.59, 115.97, 83.55, 22.22, 19.34, 17.37, 11.35. $[a]^{28}$D +35.0 (c

5.00, EtOH). Melting point: 273.8 to 279.0 °C. *m/z* (ESI, +ve ion): 325.1 $(M+H)^+$. HRMS calculated for: $C_{18}H_{21}N_4O_2$ 325.1665; found 325.1659.

^{1}H NMR RP-6306

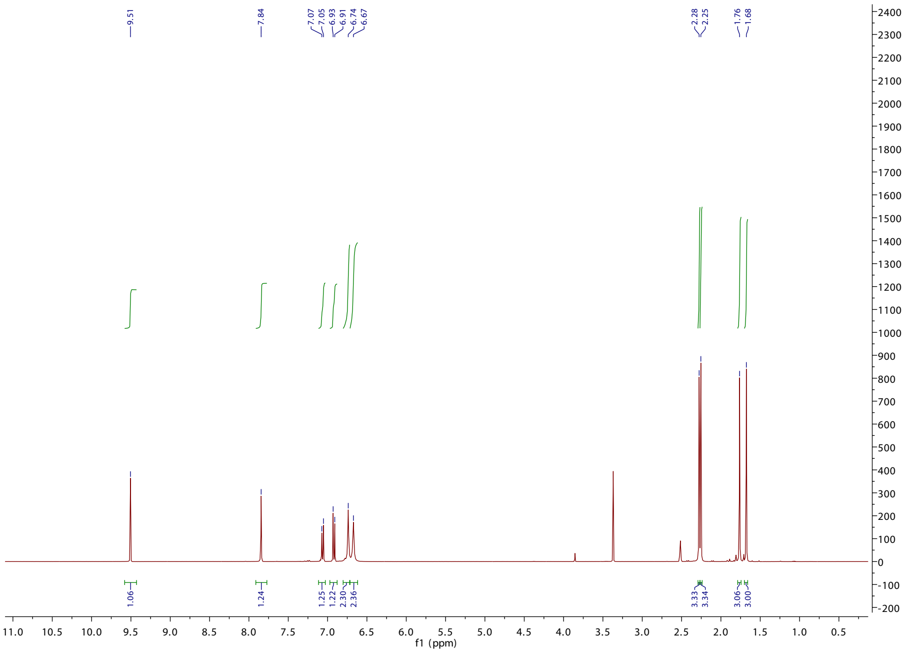

^{13}C NMR RP-6306

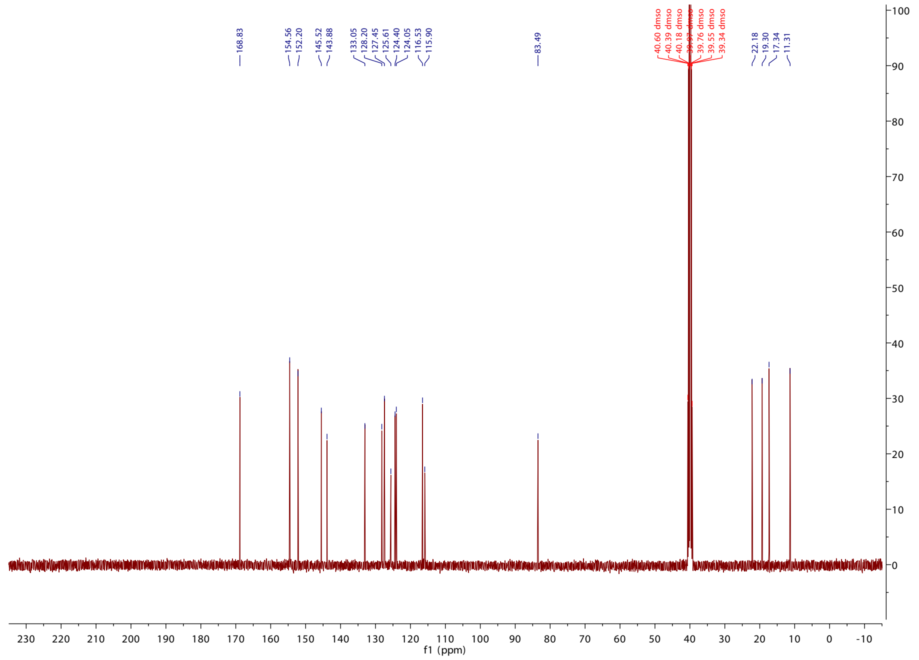

HRMS RP-6306

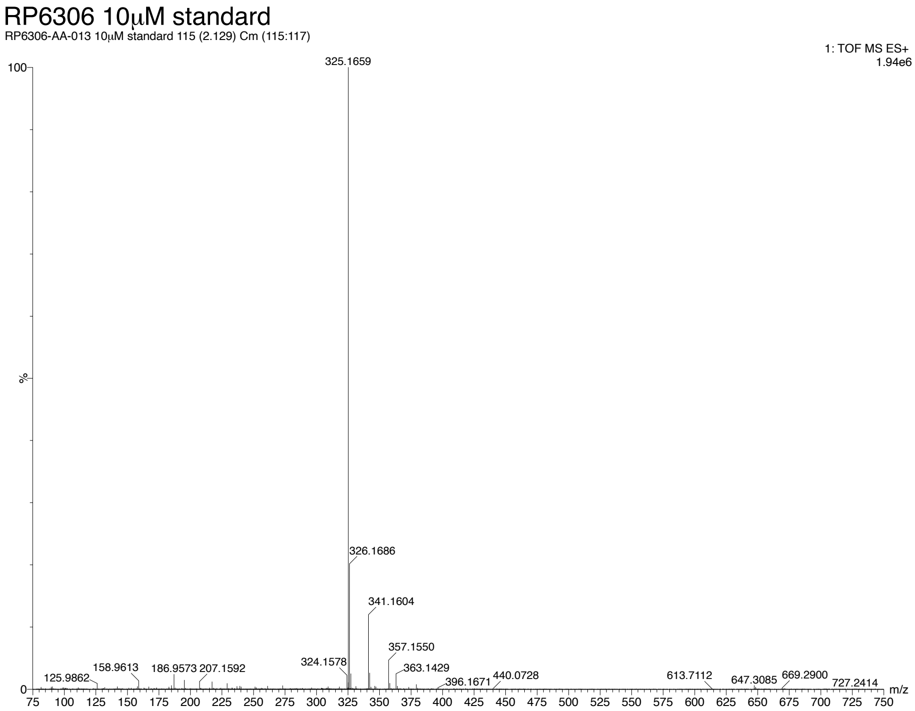

Synthesis of RP-6421

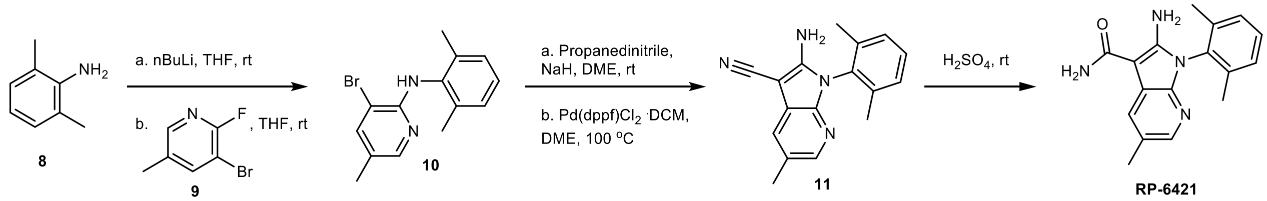

<u>3-bromo-N-(2,6-dimethylphenyl)-5-methyl-pyridin-2-amine (**10**):</u> To a solution of 2,6-dimethylaniline (**8**; 769 mg, 6.35 mmol, Sigma-Aldrich, D146005) in THF (10 mL) was added nBuLi (2.5 M solution in hexanes, 2.50 mL, 6.25 mmol) dropwise. An exotherm was observed. After 10 min, the dark anion solution was added dropwise by cannula to a solution of 3-bromo-2-fluoro-5-methyl-pyridine (**9**; 1.0 g, 5.26 mmol, Combi-Blocks OS-2139) in THF (10 mL) and the resulting mixture was stirred at ambient temperature for 1h. The reaction mixture was then quenched with saturated aqueous NH_4Cl and H_2O (1:1) and extracted with EtOAc. The organic layer was washed with brine, dried over anhydrous sodium sulfate, filtered and concentrated. Chromatographic purification of the residue (silica gel, gradient of 0-30% EtOAc in heptanes) provided **10** (1.04g, 68% yield) as a dark orange oil. *m/z* (ESI, +ve ion): 293.0 $(M+H)^+$.

2-amino-1-(2,6-dimethylphenyl)-5-methyl-pyrrolo[2,3-b]pyridine-3-carbonitrile (**11**): To a suspension of sodium hydride (60% dispersion in mineral oil, 160 mg, 4.00 mmol) in DME (5 mL) in a vial was added a solution of propanedinitrile (235 mg, 3.56 mmol) in DME (2.5 mL) dropwise and the resulting mixture was stirred for 25 min at ambient temperature. Compound **10** (500 mg, 1.72 mmol) was added, the flask was rinsed with DME (1 mL), and Pd(dppf)Cl_2•DCM (72 mg, 0.088 mmol) was added. The reaction mixture was flushed with N_2 bubbling through the solution, the vial was sealed and stirred at 100 °C for 1.25h. The reaction mixture was cooled to RT, diluted with H_2O and saturated aqueous NH_4Cl and extracted with DCM (3x) using a phase separator. The combined organic extracts were adsorbed on silica using DCM/MeOH and the residue was purified by flash chromatography (silica gel, gradient of 0-100% EtOAc in heptanes) to provide 11 (412 mg, 87% yield) as a peach sticky solid. *m/z* (ESI, +ve ion): 277.1 $(M+H)^+$.

2-amino-1-(2,6-dimethylphenyl)-5-methyl-pyrrolo[2,3-b]pyridine-3-carboxamide (**RP-6421**): Compound 11 (412 mg, 1.49 mmol) was stirred in concentrated sulfuric acid (18 M, 4 mL) for 1h. The reaction mixture was quenched with crushed ice, placed in an ice bath, and made alkaline (pH 8-9) with 1:1 H_2O/NH_4OH. The solids were collected by filtration on Buchner, washed with H_2O and air-dried overnight. The residue was purified by flash chromatography (silica gel, gradient of 0-100% EtOAc in heptanes) to provide material (351 mg, 80% yield) as a pink solid which was triturated in MeCN, collected by filtration on Buchner and air-dried, affording **RP-6421** (100 mg, 23% yield) as a white solid. ^{1}H NMR (400 MHz, DMSO-d6) δ 7.86 (dd, J = 1.9, 0.8 Hz, 1H), 7.59 (dd, J = 1.9, 0.8 Hz, 1H), 7.32 – 7.24 (m, 1H), 7.19 (dd, J = 8.0, 1.9 Hz, 1H), 7.13 (d, J = 7.9 Hz, 1H), 6.93 (br s, 2H), 6.69 (br s, 2H), 2.38 (s, 3H), 2.31 (s, 3H), 1.87 (s, 3H). ^{13}C NMR (101 MHz, dmso) δ 168.29, 152.95, 144.70, 138.65, 138.18, 136.71, 131.57, 130.02, 129.26, 127.62, 125.64, 123.99, 118.01, 83.18, 40.15, 39.94, 39.73, 39.52, 39.31, 39.10, 38.89, 20.72, 18.07, 17.16. Melting point: 141.4 to 156.1 °C. *m/z* (ESI, +ve ion): 295.2 $(M+H)^+$. HRMS calculated for $C_{17}H_{19}N_4O$ 295.1559; found 295.1551.

^{1}H NMR RP-6421

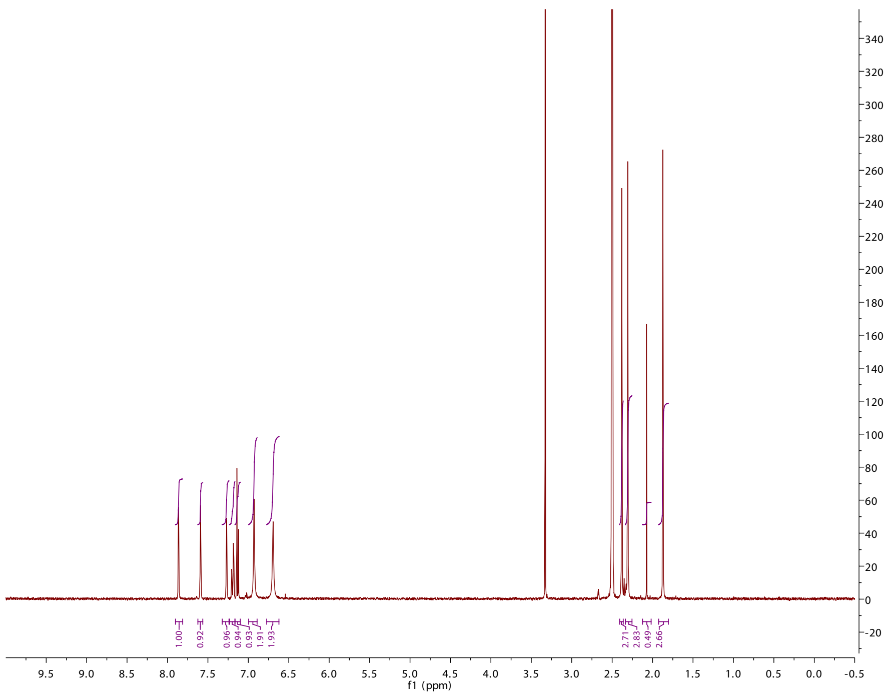

^{13}C NMR RP-6421

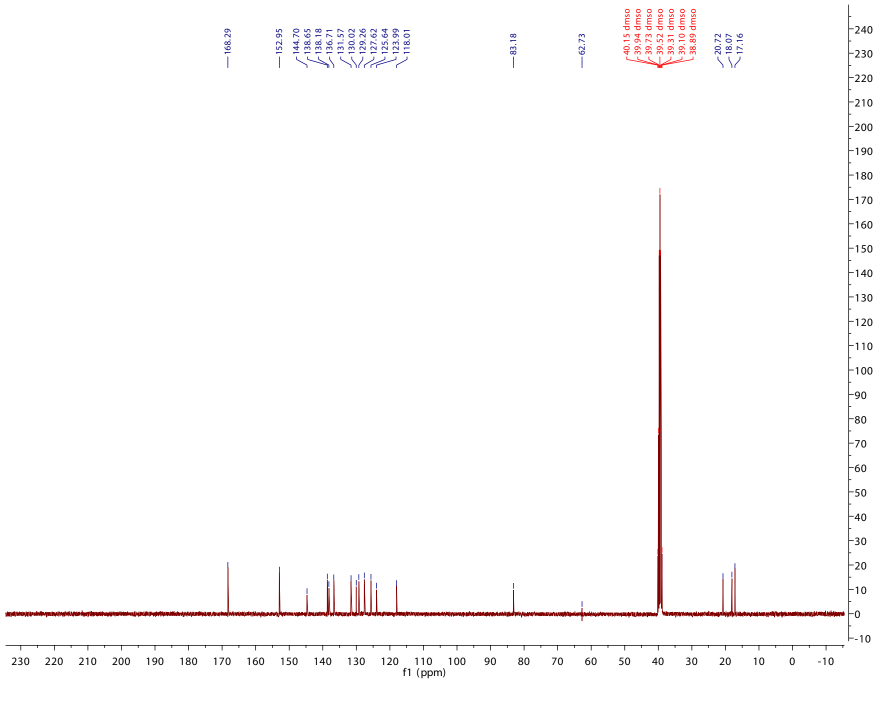

HRMS RP-6421

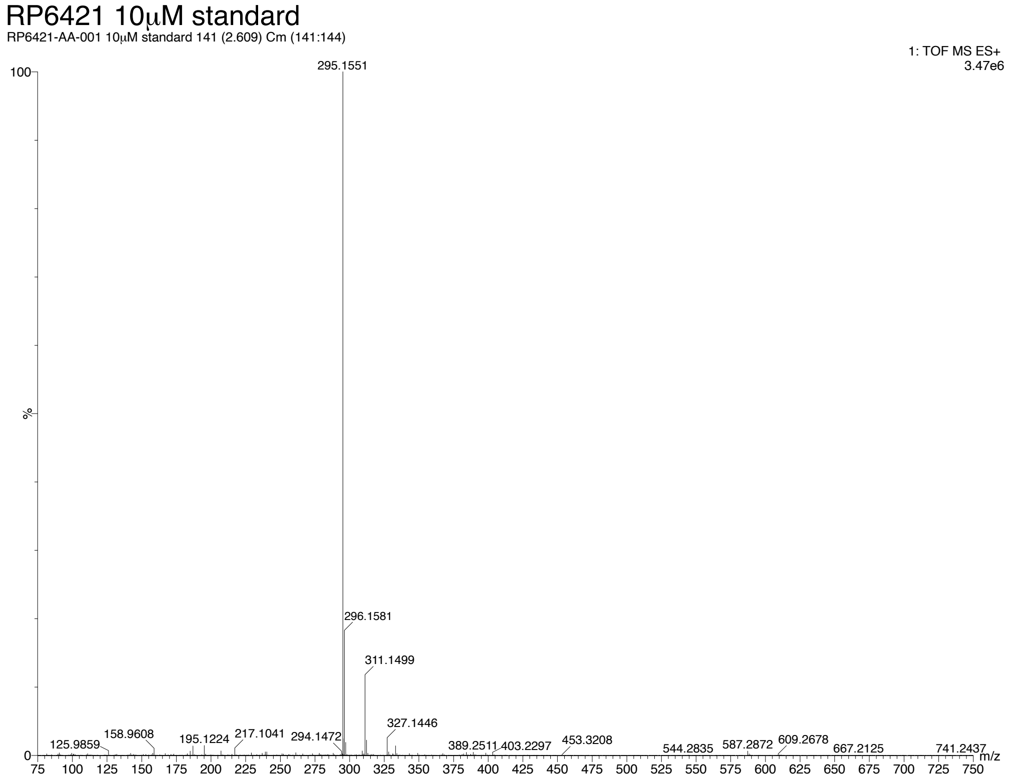
RP6421 10μM standard
RP6421-AA-001 10μM standard 141 (2.609) Cm (141:144)
1: TOF MS ES+
3.47e6
295.1551
296.1581
311.1499
327.1446
125.9859
158.9608
195.1224
217.1041
294.1472
389.2511
403.2297
453.3208
544.2835
587.2872
609.2678
667.2125
741.2437
%
m/z

Supplementary Figure 1

ED Figure 1a,d

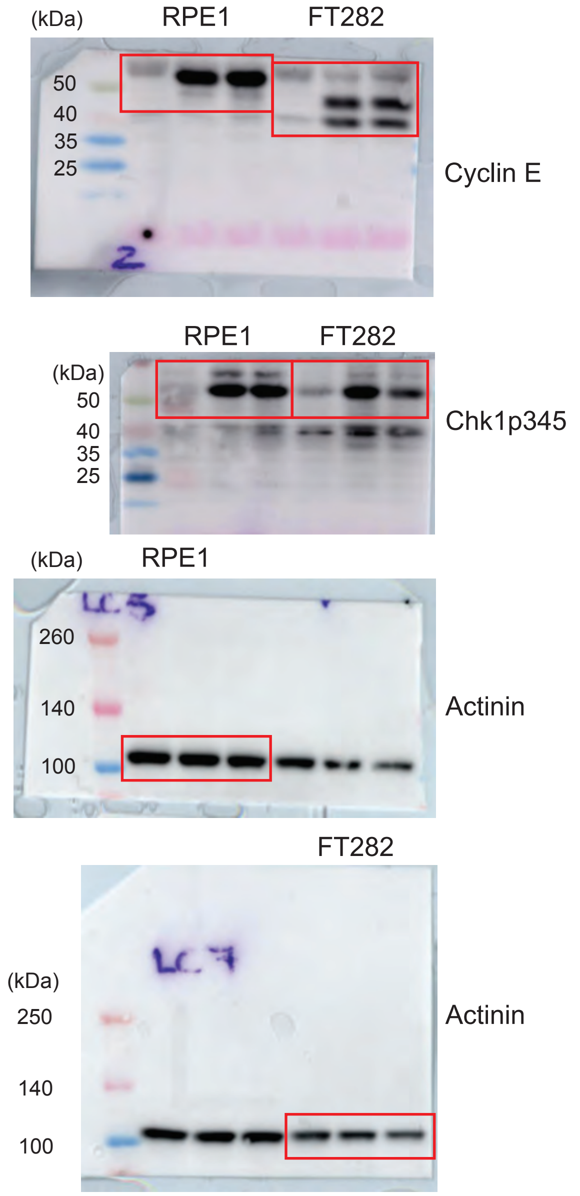

ED Figure 1g

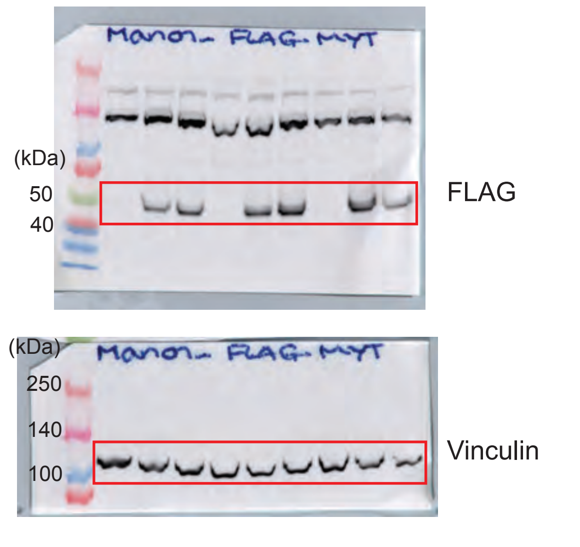

ED Figure 1i

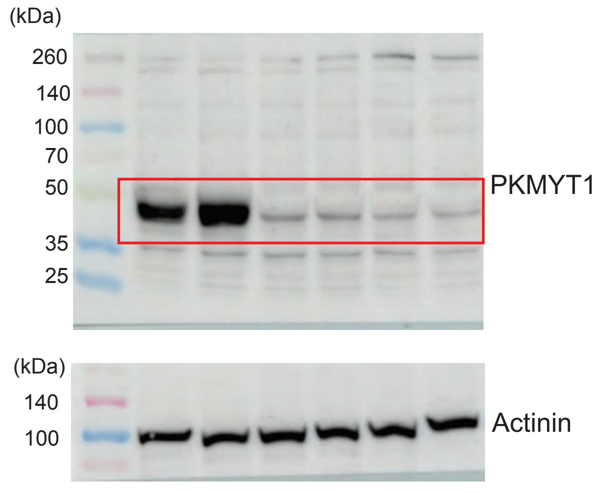

ED Figure 1j

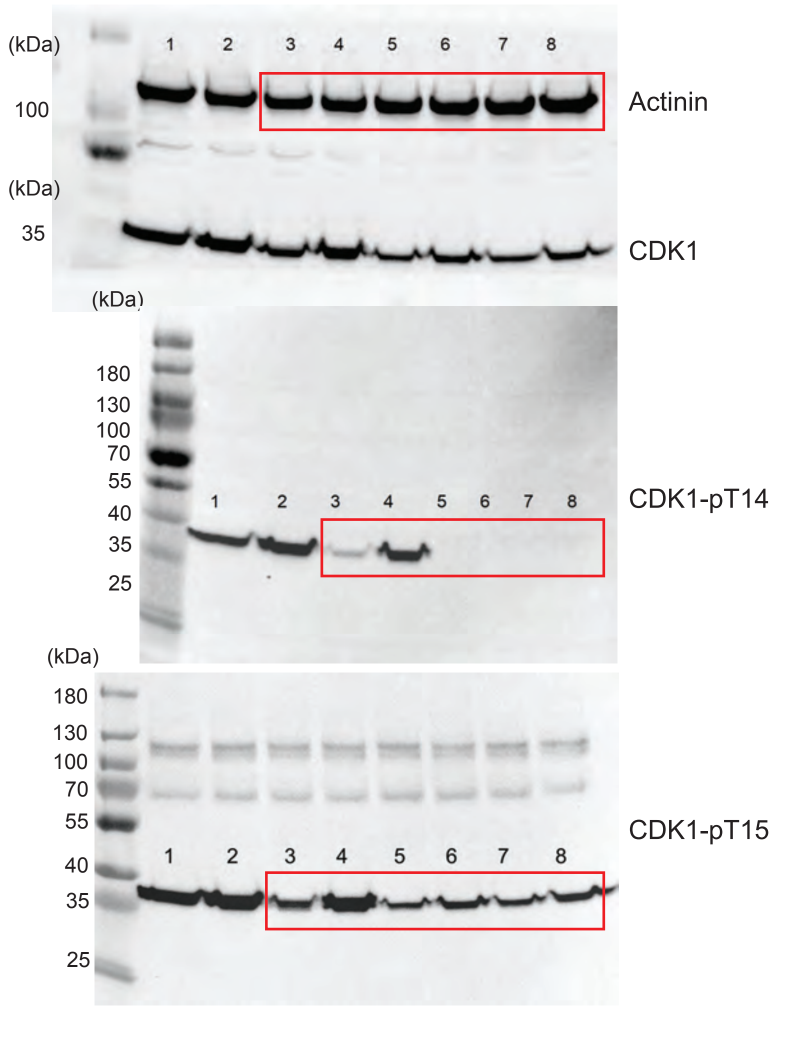

ED. figure 2c,d

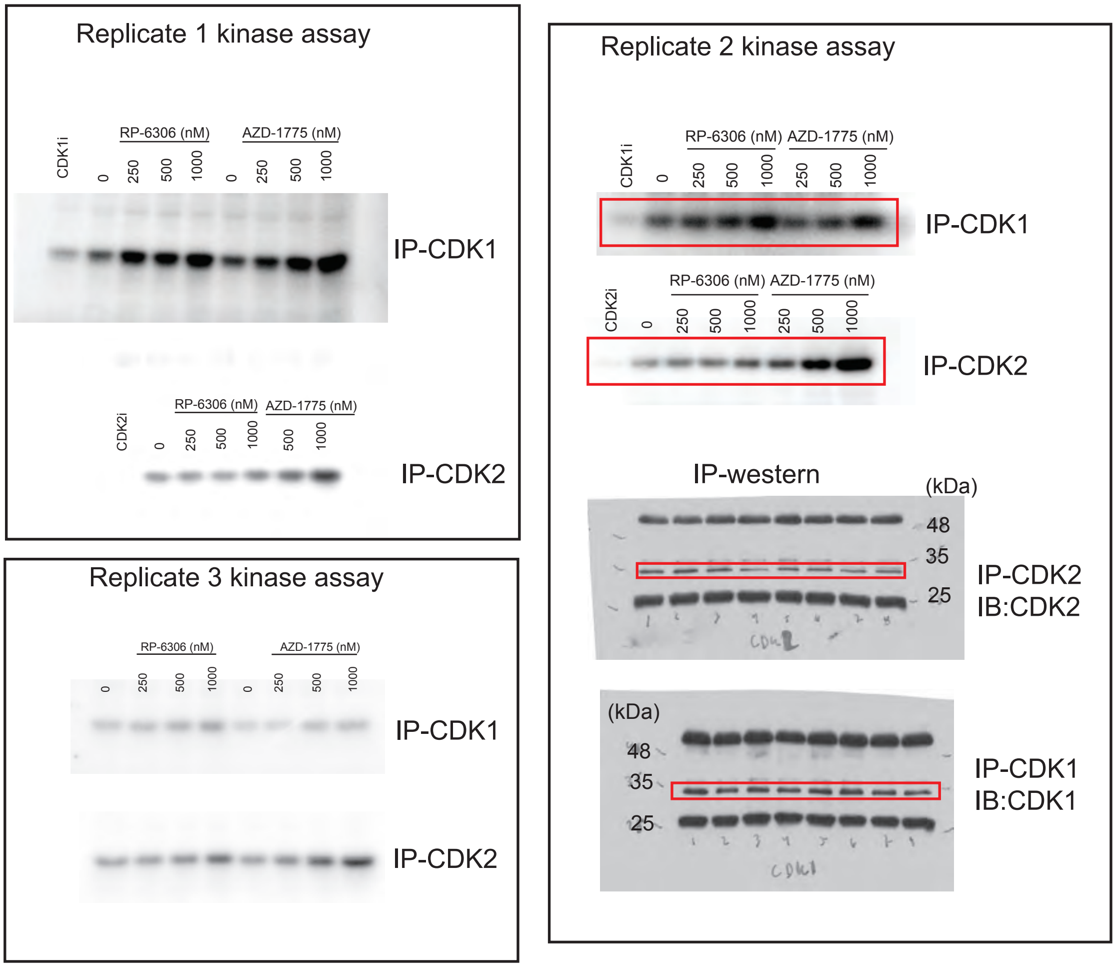

ED. figure 2f

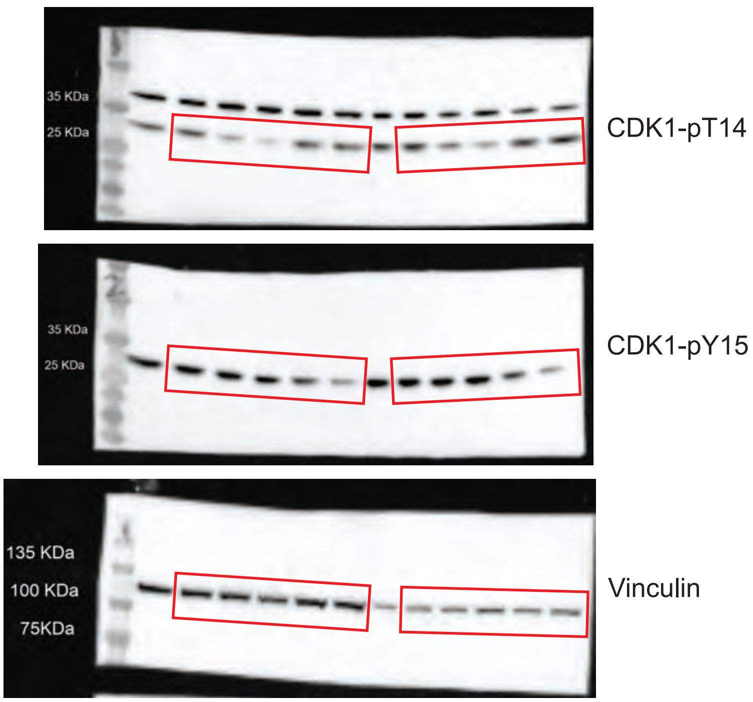

ED. figure 2i

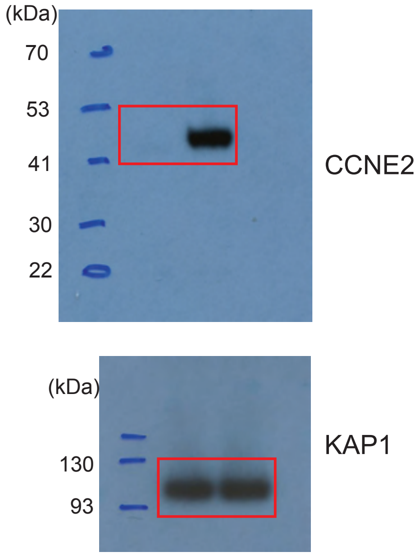

ED. figure 4f

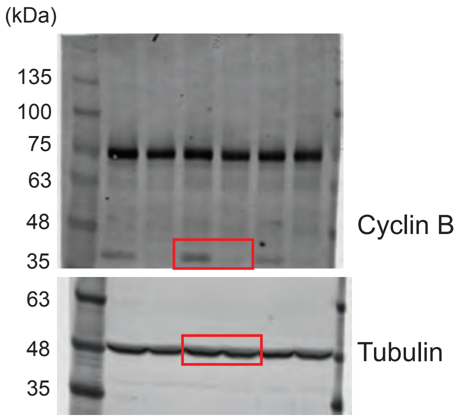

ED. figure 6e

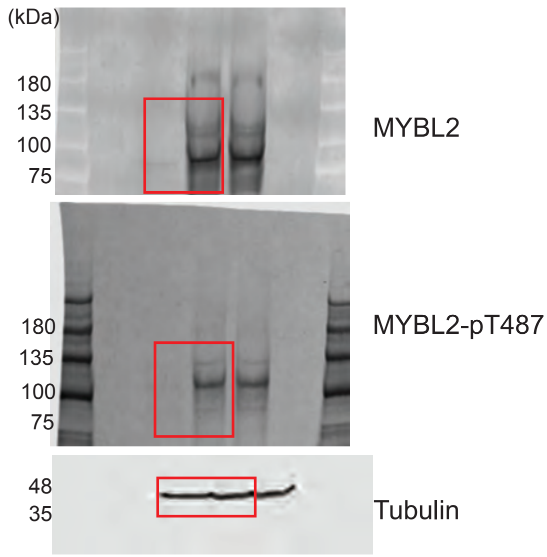

ED. figure 6f

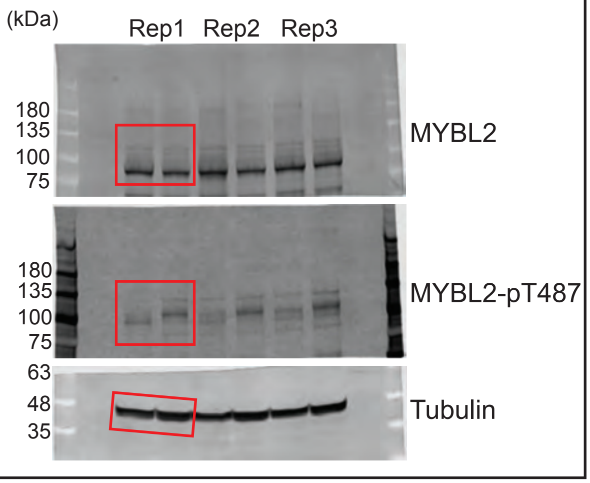

ED. figure 7a

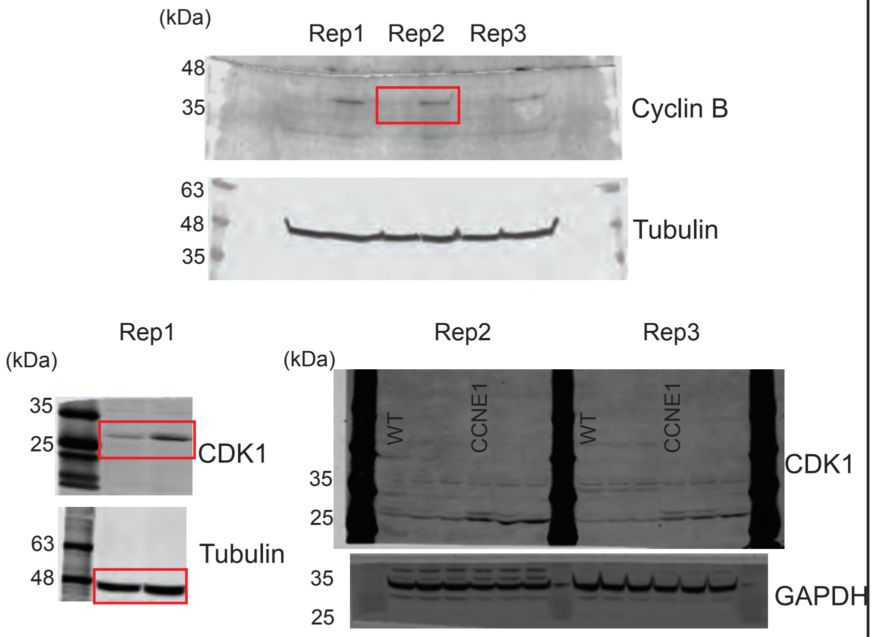

ED. figure 7h

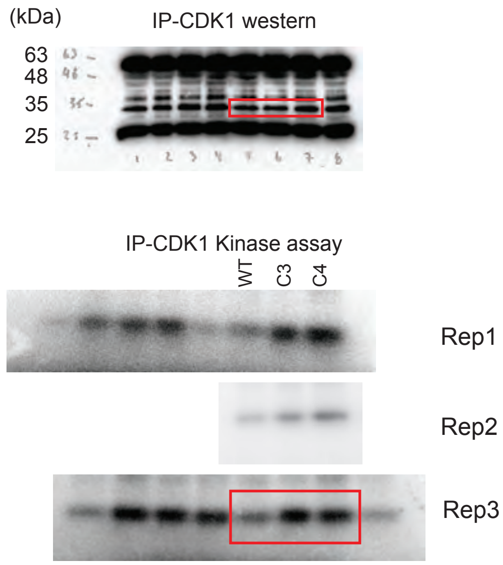

ED. figure 7i

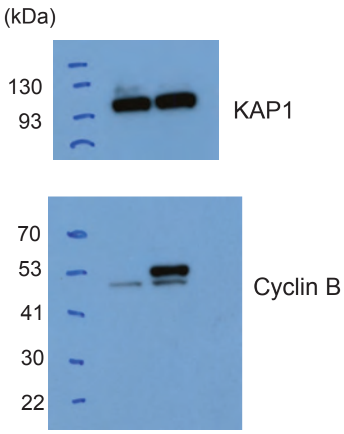

ED. figure 8e

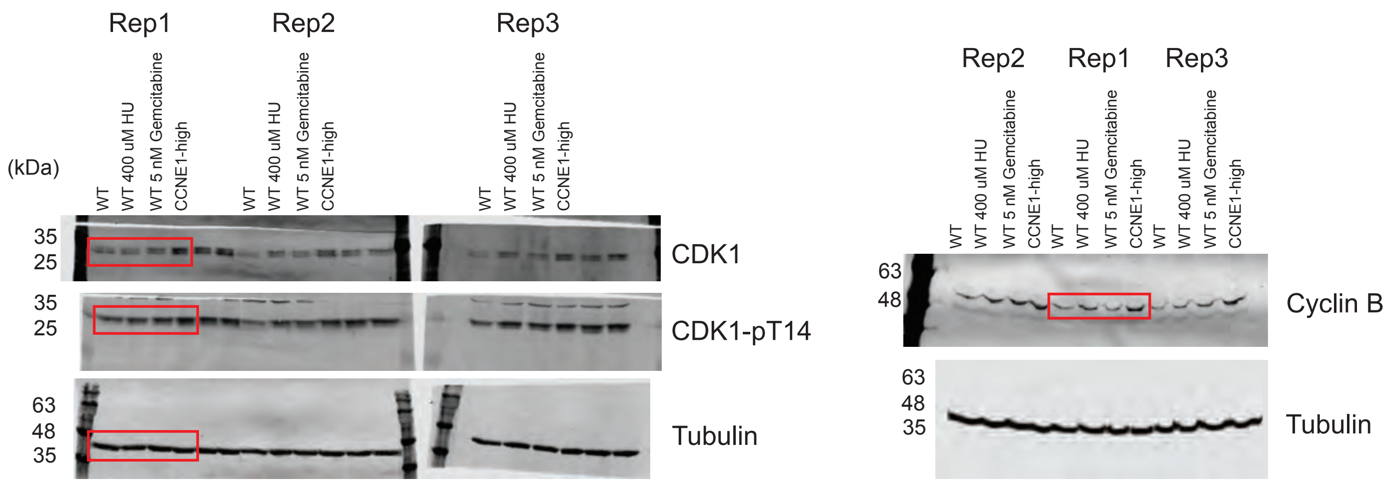

ED. figure 9a

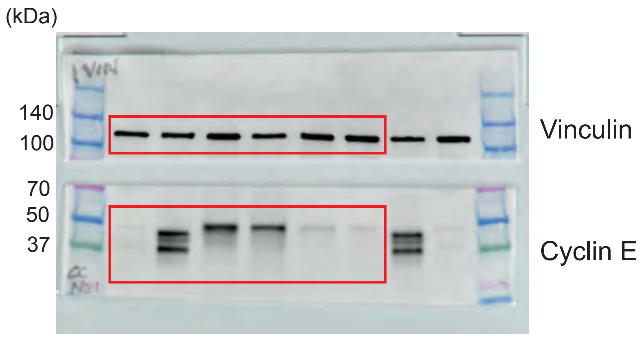

ED. figure 10b

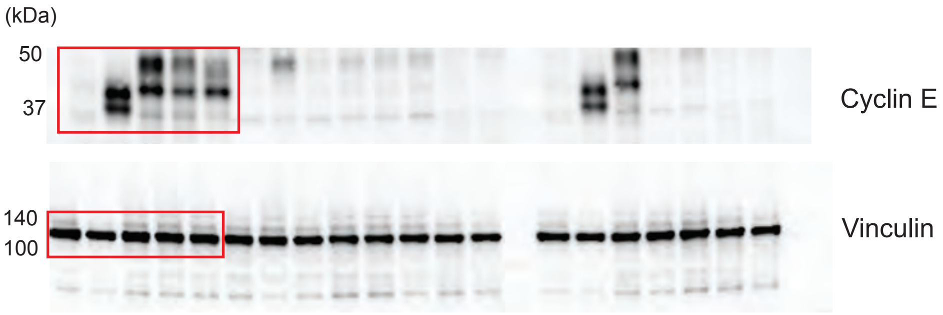

Supplementary Figure 2

a

FT282-hTERT *TP53*[R175H]

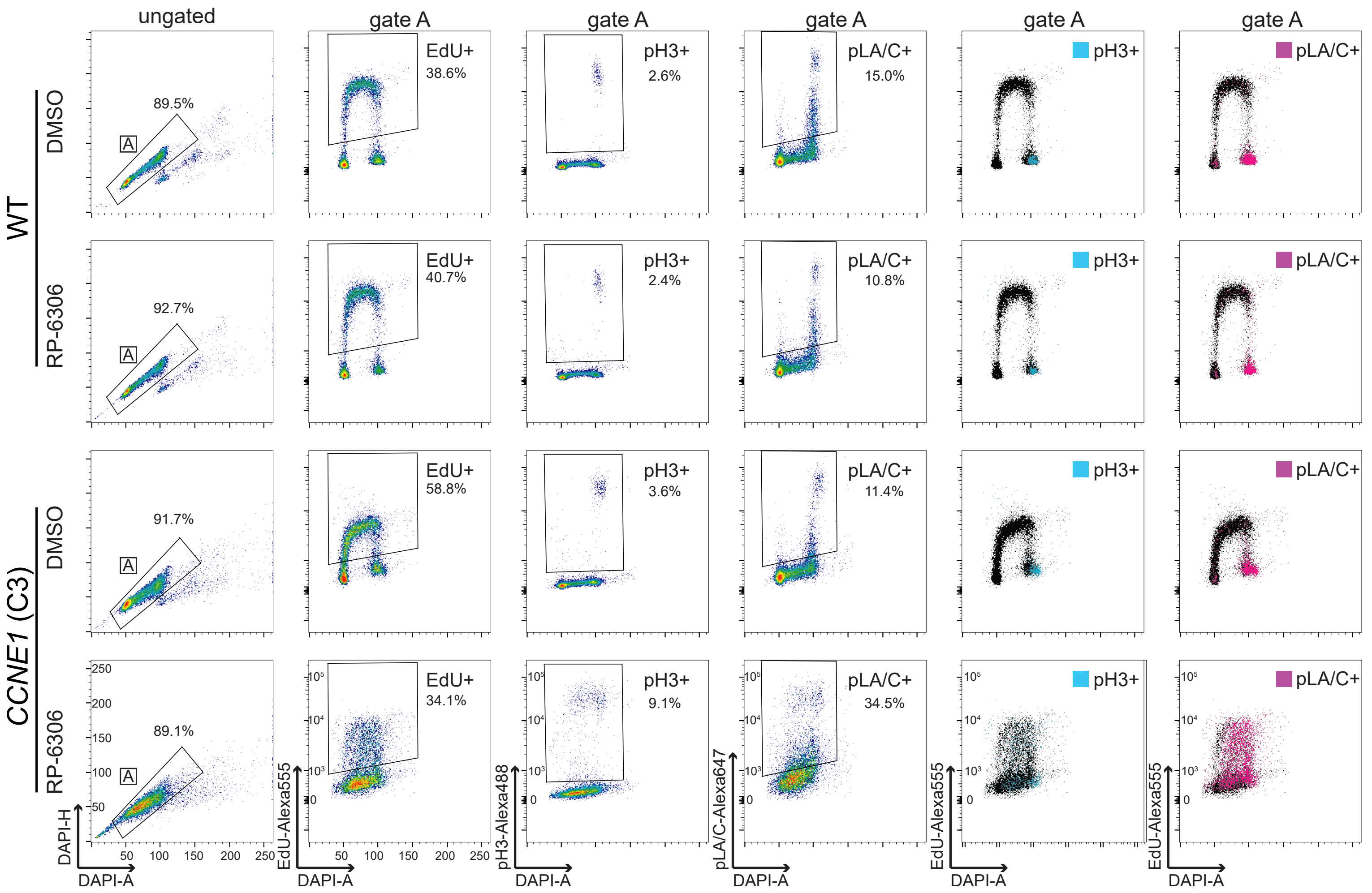

b

HCC1569

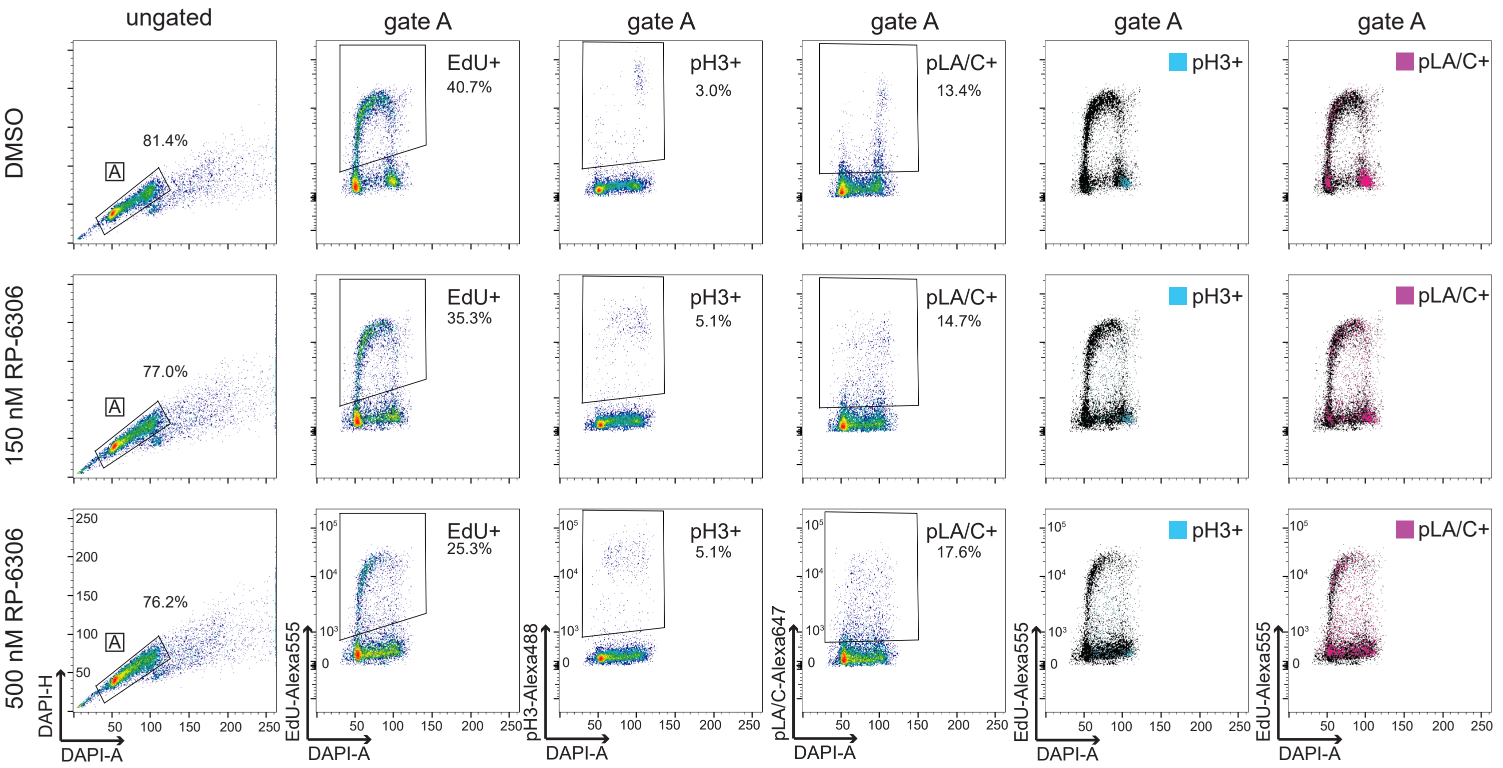

Supplementary Figure 2. FACS gating strategy. a,b,, Gating strategy for quantitation of EdU$^+$ H3-pS10$^+$ (pH3$^+$) and lamin A/C-pS22$^+$ (pLA/C$^+$) FT282-hTERT *TP53*^{R175H} (**a**) and HCC1569 (**b**) cells by FACS. Single cells were identified by gating events on DAPI-H/DAPI-A (gate A) to exclude doublets. EdU-Alexa555/DAPI, pH3-Alexa488/DAPI and pLA/C-Alexa647/DAPI were then plotted and EdU$^+$, H3-pS10$^+$ and lamin A/C-pS22$^+$ populations were identified using the gates displayed.

Supplementary Figure 3

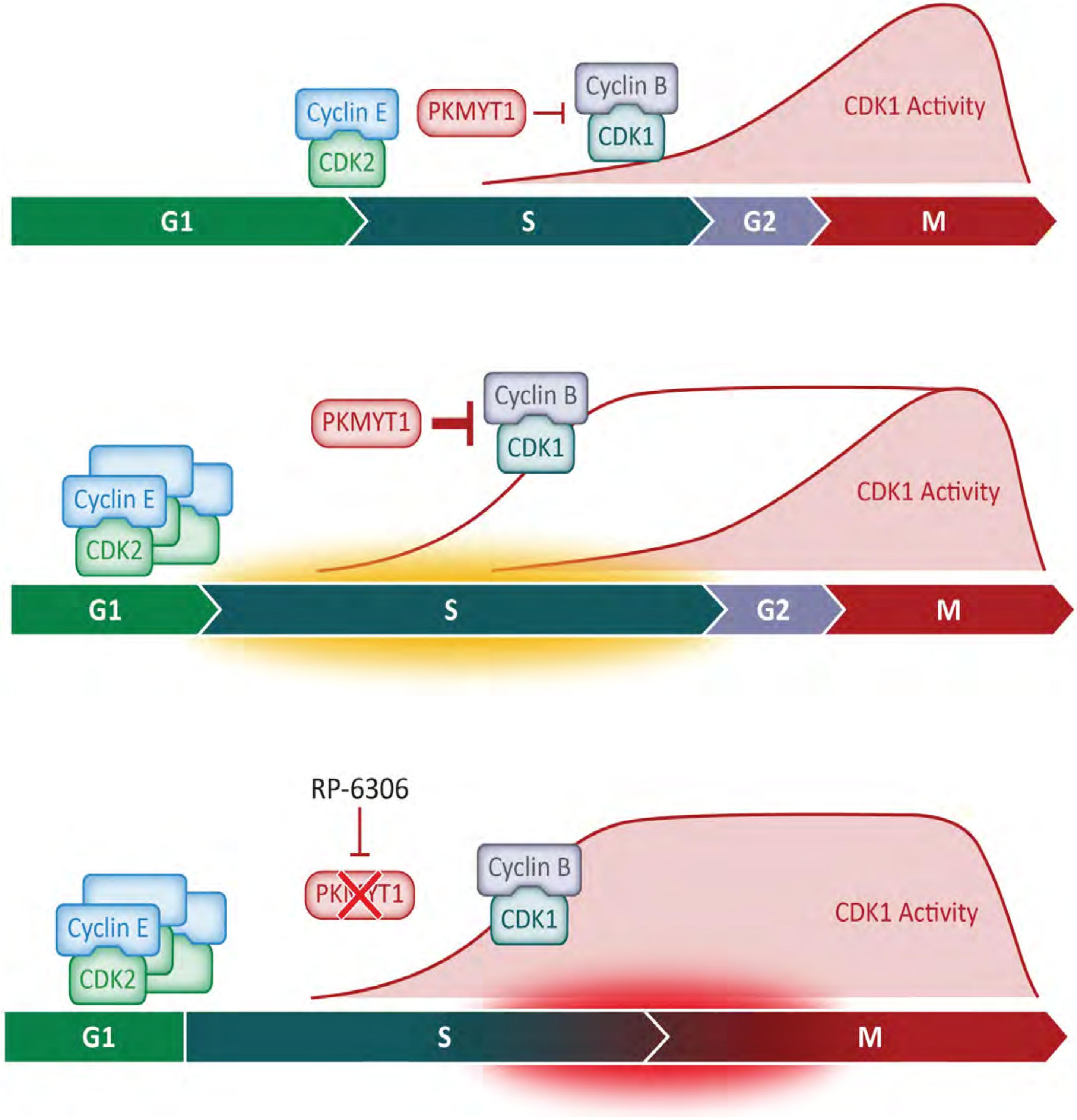

Supplementary Figure 3. ***Model of synthetic lethal relationship between CCNE1 amplification and PKMYT1 inhibition.*** In healthy cells cyclin B-CDK1 levels rise in the cytoplasm in G2-phase where PKMYT1 restricts CDK1 activation and nuclear translocation until the onset of mitosis. In pathological conditions when CCNE1 is amplified or overexpressed cells suffer from replication stress and cyclin B-CDK1 levels escalate earlier in the cell cycle from enhanced MMB-FOXM1 transcription. These conditions render PKMYT1 essential to keep CDK1 activity low until genome duplication is complete. Genetic ablation or pharmacological inhibition of PKMYT1 in *CCNE1*-amplified cells hyperactivates CDK1 and triggers mitosis before DNA replication is complete which pulverizes chromosomes causing catastrophic DNA damage and cell death.

Quantitative image-based cytometry (QIBC) workflow for cell cycle measurements

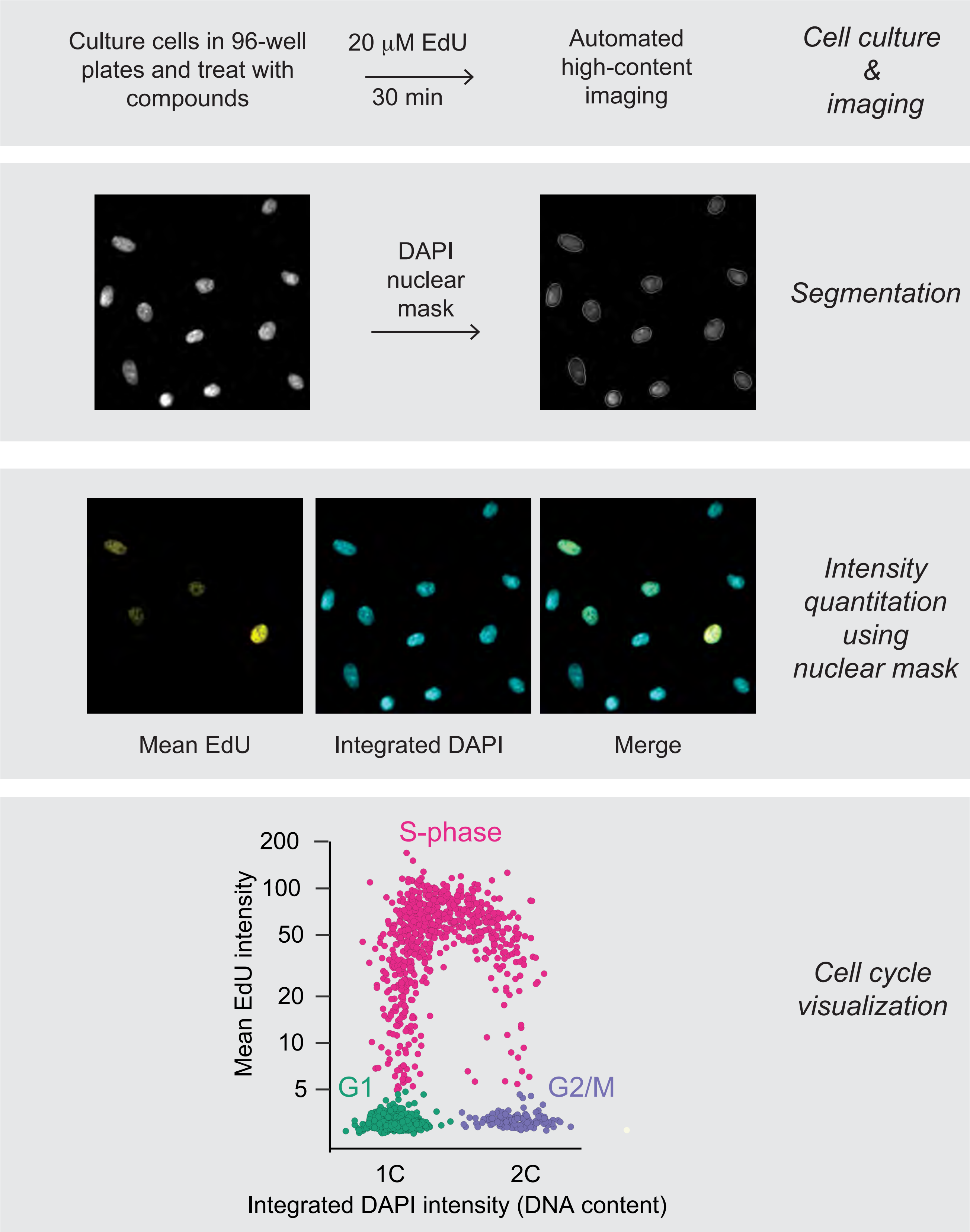

Supplementary Figure 4. ***Quantitative image-based cytometry (QIBC) workflow***. Cells were plated in 96-well plates and subjected to desired experimental treatments. Before harvesting, cells were pulsed with 20 μM EdU for 30 min to label nascent DNA. Plates were subjected to immunofluorescence analysis with antibodies directed against proteins and post-translational modifications of interest, DAPI to stain DNA and click chemistry to detect EdU incorporation in cells undergoing DNA replication. Following high-content image acquisition, nuclei were segmented using the DAPI channel and a nuclear mask was applied to each channel to quantitate the integrated DAPI intensity, mean EdU intensity and mean intensity of staining with other antibodies. The low and high end of the DAPI distribution corresponds to one genome copy (1C) or two genome copies (2C) respectively. To visualize cell cycle distributions, mean EdU intensity was plotted as a function of integrated DAPI intensity which allowed for clear identification of cells in G1 (1C, EdU^-), S-phase (EdU^+) and G2/M (2C, EdU^-).

Supplementary Table 2. Summary of TIDE editing analysis performed in the course of this study

Experiment	Cell line	Genotype	sgRNA used	indel score (%)
Related to Fig. 1d,e	RPE1 hTERT Cas9 *TP53*$^{-/-}$	WT	*PKMYT1*-4	85
			PKMYT1-6	91
			WEE1-1	94
			WEE1-3	91
			CDK2-1	81
			CDK2-2	96
		CCNE1-2A-GFP (C2)	*PKMYT1*-4	89
			PKMYT1-6	93
			WEE1-1	91
			WEE1-3	86
			CDK2-1	77
			CDK2-2	93
		CCNE1-2A-GFP (C21)	*PKMYT1*-4	81
			PKMYT1-6	97
			WEE1-1	89
			WEE1-3	96
			CDK2-1	73
			CDK2-2	89
Related to Fig. 1f,g	FT282 hTERT *TP53*^{R175H}	WT	*PKMYT1*-4	88
			PKMYT1-6	92
			WEE1-1	NA
			WEE1-3	NA
			CDK2-1	78
			CDK2-2	98
		CCNE1 (C3)	*PKMYT1*-4	91
			PKMYT1-6	90
			WEE1-1	NA
			WEE1-3	NA
			CDK2-1	89
			CDK2-2	94
		CCNE1 (C4)	*PKMYT1*-4	86
			PKMYT1-6	89
			WEE1-1	NA
			WEE1-3	NA
			CDK2-1	91
			CDK2-2	90
Related to Extended Data Fig.3g	FT282 hTERT *TP53*^{R175H}	WT	*PKMYT1*-4	90
			PKMYT1-6	88
		CCNE1 (C3)	*PKMYT1*-4	89
			PKMYT1-6	84

Related to Extended Data Fig.5e,f	FT282 hTERT *TP53*^{R175H}	*CCNE1* (C3)	*LMNA1-1*	81
Related to Fig. 4c and Extended Data Fig.6c	FT282 hTERT *TP53*^{R175H}	*CCNE1* (C3)	*MYBL2-1*	94
			MYBL2-2	90
			LIN54-1	68
			LIN54-2	85
			FOXM1-2	69
			FOXM1-3	90
Related to Extended Data Fig.6d and Extended Data Fig. 7f,g	FT282 hTERT *TP53*^{R175H}	*CCNE1* (C3)	*MYBL2-1*	94
			MYBL2-2	83
			LIN54-1	87
			LIN54-2	91
			FOXM1-2	86
			FOXM1-3	90

Supplementary Table 3. Pharmacokinetic parameters of RP-6306 in mice

PK parameter	RP-6306
CL (mL/min/kg)	30.4 ± 1.3
V_{ss} (L/kg)	2.46 ± 0.67
$T_{1/2\ oral}$ (h)	1.58 ± 0.35
%F	37.4 ± 0.9

CL = Apparent total clearance of the drug from blood, V_{ss} = Apparent volume of distribution at steady state, $T_{1/2oral}$ = Observed oral half-life, %F = Oral bioavailability

Supplementary Table 4. MMB-FOXM1 transcriptional signature

MMB-FOXM signature		
UBE2C	KPNA2	KIF14
CDC20	CDK1	TOP2A
CENPA	NUF2	CHEK2
KIF2C	KIF23	FAM83D
AURKB	CDKN3	HMMR
PLK1	SPC25	CENPF
CDCA8	DEPDC1	ARHGAP11B
KIF18B	KIF11	ASPM
TPX2	CKAP2L	H2AFZ
CCNB2	FAM64A	INCENP
CCNB1	PRC1	ECT2
HJURP	PTTG1	FBXO5
TTK	LMNB1	KNSTRN
CCNA2	ARHGAP11A	RFWD3
PIF1	KIF18A	HMGB2
ESPL1	NEK2	CKAP2
AURKA	NUSAP1	CDCA2
DLGAP5	SPAG5	KIF20B
UBE2S	NCAPD2	ATAD2
KIF20A	RACGAP1	TAF5

Supplementary Table 5. sgRNA guide sequences

sgRNA	sequence
PKMYT1-4	TCACCGGCAGTGAACTCAGG
PKMYT1-6	GGGCCATGGCTCCTACGGAG
PKMYT1-7	CAGGCCTCACAGTGTTGCTG
WEE1-1	GTCGCCGGTCAAGTCGCCGG
WEE1-3	TCATCAACAGAGCCCGCCAA
CDK2-1	AAGCAGAGAGATCTCTCGGA
CDK2-2	AATGGCAGAAAGCTAGGCCC
LMNA-1	CATCGACCGTGTGCGCTCGC
MYBL2-1	CAAGGTCAAATGGACCCATG
MYBL2-2	CTGTCCAAACTGCCTCACCA
LIN54-1	GGTGAATAGTTTGCTTCCAG
LIN54-2	CTGTTGGGTCTGTAACACAG
FOXM1-2	CACTGATTCTCAAAAGACGG
FOXM1-3	ATAGCCTATCCAACATCCAG
