# Supplementary material for: CCNE1 amplification is synthetic lethal with PKMYT1 kinase inhibition
Source: Nature. 2022 Apr 20;604(7907):749–56. doi: 10.1038/s41586-022-04638-9 (PMC9046089; doi:10.1038/s41586-022-04638-9)
Supplement: Supplementary file 2 — Reporting Summary [file 41586_2022_4638_MOESM2_ESM.pdf]

nature research

Corresponding author(s): Daniel Durocher & Gary Marshall

Last updated by author(s): Feb 25, 2022

Reporting Summary

Nature Research wishes to improve the reproducibility of the work that we publish. This form provides structure for consistency and transparency in reporting. For further information on Nature Research policies, see our Editorial Policies and the Editorial Policy Checklist.

Statistics

For all statistical analyses, confirm that the following items are present in the figure legend, table legend, main text, or Methods section.

n/a	Confirmed	
☐	☒	The exact sample size (*n*) for each experimental group/condition, given as a discrete number and unit of measurement
☒	☐	A statement on whether measurements were taken from distinct samples or whether the same sample was measured repeatedly
☐	☒	The statistical test(s) used AND whether they are one- or two-sided<br>*Only common tests should be described solely by name; describe more complex techniques in the Methods section.*
☒	☐	A description of all covariates tested
☒	☐	A description of any assumptions or corrections, such as tests of normality and adjustment for multiple comparisons
☐	☒	A full description of the statistical parameters including central tendency (e.g. means) or other basic estimates (e.g. regression coefficient) AND variation (e.g. standard deviation) or associated estimates of uncertainty (e.g. confidence intervals)
☐	☒	For null hypothesis testing, the test statistic (e.g. *F*, *t*, *r*) with confidence intervals, effect sizes, degrees of freedom and *P* value noted<br>*Give P values as exact values whenever suitable.*
☒	☐	For Bayesian analysis, information on the choice of priors and Markov chain Monte Carlo settings
☒	☐	For hierarchical and complex designs, identification of the appropriate level for tests and full reporting of outcomes
☒	☐	Estimates of effect sizes (e.g. Cohen's *d*, Pearson's *r*), indicating how they were calculated

Our web collection on statistics for biologists contains articles on many of the points above.

Software and code

Policy information about availability of computer code

Data collection	Flow cytometry: FACSDIVA v8.0.1<br>Clonogenics: GelCount(Oxford Optronix)<br>Cell proliferation assays: Incucyte S3 Software (Sartorius)<br>Synergy scores: SynergyFinder v2.0 (https://synergyfinder.fimm.fi)<br>Immunoblotting: Image Studio Lite v5.2.5<br>Immunofluorescence and cytogenetics: ZEN 2.3 SP1<br>Kinase assays: Typhoon FLA 9500 Control Software ((GE Healthcare Life Sciences)<br>QIBC: InCell Analyzer 6000 Acquisition Software v1.0<br>ADP-GLO, NanoBRET and ELISA AlphaLISA: EnVision plate reader (Perkin-Elmer)<br>Immunohistochemistry: Aperio AT2 (Leica Biosystems)<br>Animal studies: Studylog v4.4
Data analysis	Data visualization and statistical analysis: GraphPad PRISM 9; RStudio v1.2.5019; Microsoft Excel 2011<br>QIBC: Cellprofiler 3.1.9<br>Immunofluorescence, Kinase assays and cytogenetics: ImageJ v2.0.0<br>Immunoblotting: Image Studio Lite v5.2.5<br>Flow cytometry: FlowJo v10<br>RNA-seq: FastQC v0.11.9; Salmon v1.4.0; edgeR v3.30.3; heatmap3 v1.1.9<br>Immunohistochemistry: HALO (Indica labs)

For manuscripts utilizing custom algorithms or software that are central to the research but not yet described in published literature, software must be made available to editors and reviewers. We strongly encourage code deposition in a community repository (e.g. GitHub). See the Nature Research guidelines for submitting code & software for further information.

Data

Policy information about availability of data

All manuscripts must include a data availability statement. This statement should provide the following information, where applicable:

- Accession codes, unique identifiers, or web links for publicly available datasets
- A list of figures that have associated raw data
- A description of any restrictions on data availability

CRISPR screen raw counts of sequencing reads with quality scores in FASTQ format have been deposited in NCBI's SRA and are accessible through BioProject accession number PRJNA808613 (https://www.ncbi.nlm.nih.gov/bioproject/808613). Read counts of the CRISPR screens in figure 1a-b and 4a are available in the files Dataset 1, Dataset 2 and Dataset3. CRISPR dependency data (CERES scores) and gene-level copy number data were downloaded from the 2021 Q1 DepMap release (https://depmap.org/portal/download). RNA-seq raw counts of sequencing reads with quality scores in FASTQ format and normalized transcript abundance measurements in figure 4b and Extended data figure 8a,b have been deposited in NCBI's Gene Expression Omnibus and are accessible through GEO Series accession number GSE171453 (https://www.ncbi.nlm.nih.gov/geo/query/acc.cgi?acc=GSE171453). FASTQ files were aligned to the GENCODE GRCh38 v36 primary assembly of the human genome (https://www.gencodegenes.org/human/release_36). All data supporting the findings of this study are available from the corresponding authors on reasonable request.

Field-specific reporting

Please select the one below that is the best fit for your research. If you are not sure, read the appropriate sections before making your selection.

☒ Life sciences ☐ Behavioural & social sciences ☐ Ecological, evolutionary & environmental sciences

For a reference copy of the document with all sections, see nature.com/documents/nr-reporting-summary-flat.pdf

Life sciences study design

All studies must disclose on these points even when the disclosure is negative.

Sample size

All Genetic screens were conducted once with two technical replicates. In vitro cell biology experiments were completed with at least 3 replicates or more if indicated. Kinase assays were completed with 3 replicates with one representative phosphor image displayed. RNA-seq was conducted with 3 biological replicates for each sample For QIBC experiments a minimum of 1000 cells was used for each measurement. For flow cytometry a minimum of 9,000 single cells was used for each measurement. Immunoblots were conducted once unless indicated. For in vivo studies N=3 mice were used for PKPD studies with n=2 technical replicates in the ELISA per sample and N=6-8 mice were used for efficacy studies. All sample sizes were chosen based on standard practices in the field.

Data exclusions

No data was excluded from the in vitro analyses. The following exclusions are from the in vivo efficacy studies:
Figure 5b: 2 mice died on day 12 in the vehicle group (their tumor volumes and body weight is included up to day 11)
1 mouse in the 10 mg/kg treatment group had a tumor volume that was an outlier determined by ROUT's test (Prism) and was excluded
Figure 5d: 1 mouse in the 7.5 mg/kg treatment group had a tumor volume that was an outlier determined by ROUT's test (Prism) and was excluded
Figure 5d: 1 mouse in the 10 mg/kg treatment group had a tumor volume that was an outlier determined by ROUT's test (Prism) and was excluded.
No dose holidays were given in any of the experiments.

Replication

In addition to the noted biological replicates within experiments, we demonstrated that PKMYT1 is essential in two distinct clones in two different cell lines engineered to over express CCNE1 (RPE1 C2, C21 and FT282 C3, C4). We show that three different CCNE1-amplified cell lines (HCC1569, OVCAR3 and SNU8) are more sensitive to RP-6306 that CCNE1-normal cell lines (KYSE30, TOV112D, NUGC3) and BRCA-mutated cell lines (SUM149PT, COV362, DOTC24510). We demonstrate RP-6306 induces pan-gH2AX in CCNE1-OE (RPE1 C2 and FT282 C3) and CCNE1-amp HCC1569. We show that FT282 CCNE1-OE C3 and HCC1569 cells have pulverized chromosomes after RP-6306 treatment and that RP-6306 synergizes with HU and gemcitabine. We also demonstrate that RP-6306 has tumor growth inhibition alone or tumor regressions in combination with gemcitabine in two CDX models (HCC1569 and OVCAR3) All other attempts at replication were successful

Randomization

Samples used for in vitro and cell biology experiments were not randomized as it is not relevant to this these experiments. For QIBC analysis a fixed number of images (24-48) for each condition were collected depending on cell density. Cells were segmented, quantified and the number of cells used for each condition was reduced to match the sample with the lowest cell count. randomization was conducted using the sample_n method in R. Mice for efficacy and PK/PD experiments were randomized according to tumor volume and body weight using the "stratified" method in Studylogv4.4 software.

Blinding

In cell biology and animal experiments the investigators were not blinded during data collection and analysis as there was no explicit need to do so.

Reporting for specific materials, systems and methods

We require information from authors about some types of materials, experimental systems and methods used in many studies. Here, indicate whether each material, system or method listed is relevant to your study. If you are not sure if a list item applies to your research, read the appropriate section before selecting a response.

Materials & experimental systems

n/a	Involved in the study	
☐	☒	Antibodies
☐	☒	Eukaryotic cell lines
☒	☐	Palaeontology and archaeology
☐	☒	Animals and other organisms
☒	☐	Human research participants
☒	☐	Clinical data
☒	☐	Dual use research of concern

Methods

n/a	Involved in the study	
☒	☐	ChIP-seq
☐	☒	Flow cytometry
☒	☐	MRI-based neuroimaging

Antibodies

Antibodies used

Primaries:
histone H2A.X (phospho-S139, Cell Signalling Technologies #2577)
histone H2A.X (phospho-S139, Millipore Sigma #05-636)
CDK1 (Thermo Fisher Scientific #33-1800)
CDK1-phosphoT14 (Abcam #ab58509)
CDK1-phoshoY15 (Cell Signaling Technology #9111)
PKMYT1 (Bethyl A302-424A)
Histone H3-phosphoS10 (Cell Signaling Technology #9706)
lamin A/C (Cell Signaling Technology 4C11 #4777)
lamin A/C-phosphoS22 (Cell Signaling Technology D2B2E #13448)
Cyclin B1 (Cell Signalling Technologies #2577)
Tubulin (Millipore DM1A CP06)
CDK2 (Upstate 05-596)
Cyclin B1-phosphoS126 (Abcam ab55184)
MCM2 (BD Biosciences 610700)
MCM4 (Novus Biologicals H0004137-B01P)
CHK1-phosphoS345 (Bethyl 2348)
Cyclin E1 (Abcam ab3927 or Cell Marque #AC0120RUO)
Alpha Actinin (Millipore Sigma 05-384)
Vinculin (Cell Signaling 13901S)
Agarose-coupled primaries
CDK1 (Santa Cruz sc-54 AC)
CDK2 (Santa Cruz sc-6248 AC)
MYBL2 (Millipore MABE886)
MYBL2-pT487 (Abcam ab76009)
Secondaries:
anti-mouse Irdye 800CW, anti-rabbit IRdye 680RD (926-32210 and 926-68071; LiCOR)
anti-Mouse IgG HRP (Cedarlane # NA931-1ML)
anti-Rabbit IgG HRP (Cedarlane # 111-035-144)
anti-Rabbit IgG HRP (abcam 97051)
anti-Rabbit IgG HRP (Jackson Immunoresearch #111-035-144)
AlexaFluor488 donkey anti-rat IgG (Thermo Fisher Scientific A21208)
AlexaFluor647 donkey anti-mouse IgG (Thermo Fisher Scientific A31571)
AlexaFluor488 goat anti-mouse IgG (Thermo Fisher Scientific A11029)
AlexaFluor647 goat anti-rabbit IgG (Thermo Fisher Scientific A21244)
AlphaLISA anti-rabbit IgG Acceptor beads (Perkin Elmer #AL104C)
AlphaLISA anti-mouse IgG Donor beads (Perkin Elmer #AS104D)

Validation

The Cyclin B1-phosphoS126 and lamin A/C-phosphoS22 antibodies were validated by depletion of CCNB1 in Extended data figure 5h and depletion of LMNA in Extended data figure 7e,f. The MYBL2 and MYBL2-pT487 antibodies were validated by over-expression of MYBL2 in Extended data figure 8e. All other antibodies were not explicitly validated but were either used in previous publications and/or produced bands at the expected molecular weight or the appropriate signal

Eukaryotic cell lines

Policy information about cell lines

Cell line source(s)

RPE1-hTERT p53-/- Cas9 cells were developed by Dan Durocher (PMID: 29973717)
FT282-hTERT p53-R175H WT and CCNE1 C3 and C4 were obtain from Ronny Drapkin (PMID:24366882)
All other cell lines were obtained form:
American Type Culture Collection (ATCC): HEK293T, HCC1569, OVCAR3, TOV112D, DOTC24510
Korean Cell line Bank (KCLB): SNU8
Asterand Bioscience: SUM149PT
German Collection of Microorganisms and Cell Cultures GmbH (DSMZ): KYSE30
Millipore Sigma: COV362
The Japanese Collection of Research Bioresources (JCRB): NUGC3
Sigma: A2780

Authentication	None of the cell lines used were authenticated after reception
Mycoplasma contamination	All cell lines used tested negative for mycoplasm contamination using MycoAlert
Commonly misidentified lines (See ICLAC register)	No commonly misidentified cell lines were used in this study

Animals and other organisms

Policy information about studies involving animals; ARRIVE guidelines recommended for reporting animal research

Laboratory animals	Female CB17-SCID, SCID-beige and NOD-SCID mice (5-7 weeks old; Charles River) were used for cell line-derived xenograft studies. Female BALB/c nude mice (5-7 weeks old) were used for patient-derived xenograft studies
Wild animals	The study did not involve wild animals
Field-collected samples	The study did not involve field samples
Ethics oversight	For CDX studies animals were housed and experiments were performed at Repare Therapeutics (NEOMED site, Montreal, Canada), which is a CCAC (Canadian Council on Animal Care) accredited vivarium. Studies were conducted under a protocol approved by the NEOMED Institutional Animal Care Committee (NIACC). For PDX studies animals were housed and experiments were performed at Crown Biosciences Inc. (Taicang, China) and were reviewed and approved by the Institutional Animal Care and Use Committee (IACUC) of CrownBio prior to execution. During the study, the care and use of animals were conducted in accordance with the regulations of the Association for Assessment and Accreditation of Laboratory Animal Care (AAALAC).

Note that full information on the approval of the study protocol must also be provided in the manuscript.

Flow Cytometry

Plots

Confirm that:

☒ The axis labels state the marker and fluorochrome used (e.g. CD4-FITC).

☒ The axis scales are clearly visible. Include numbers along axes only for bottom left plot of group (a 'group' is an analysis of identical markers).

☒ All plots are contour plots with outliers or pseudocolor plots.

☒ A numerical value for number of cells or percentage (with statistics) is provided.

Methodology

Sample preparation	Cells were pulsed with 20 µM EdU (5-ethynyl-2-deoxyuridine, Life Technologies #A10044) for 30 min, collected by trypsinization, resuspended as single cells, washed 1 x in PBS and pelleted at 600x g for 3 min at 4°C. All subsequent centrifugations were performed in this manner. Cells were fixed in 4% PFA/PBS for 15 min at RT, excess ice cold PBSB (1% BSA in PBS, 0.2 [illegible]M filtered) was added before pelleting. Cells were resuspended in permeabilization buffer (PBSB, 0.5% Triton-X 100) and incubated at RT for 15 min. Excess blocking buffer (PBSB, 0.1% NP-40) was added, cells were pelleted, resuspended in blocking buffer containing primary antibodies and incubated at RT for 1 h. Excess blocking buffer containing secondary antibodies was added, cells were pelleted, resuspended in blocking buffer and incubated at RT for 30 min. Excess blocking buffer was added, cells were pelleted and washed one additional time in PBSB. Cells were resuspended in EdU staining buffer (150 mM Tris-Cl pH 8.8, 1mM $CuSO_4$, 100 mM ascorbic acid and 10 µM AlexaFluor 555 azide (Life Technologies, #A20012)) and incubated at RT for 30 min. Excess PBSB was added, cells were pelleted and washed one additional time in PBSB. Cell were resuspended in analysis buffer (PBSB, 0.5 µg/ml DAPI, 250 µg/µl RNase A (Sigma-Aldrich, #R4875)) and incubated at 37°C for 30 min or left at 4°C overnight.
Instrument	Fortessa X-20 (Becton Dickinson)
Software	Collection: FACSDIVA v8.0.1<br>Analysis: FlowJo v10
Cell population abundance	At least 9,000 cells were analyzed for each population
Gating strategy	Cell cycle analysis (SI figure XX):<br>Single cells were selected by plotting DAPI-A vs. DAPI-H and selecting cells that to exclude doublets that appear as populations with a 2:1 DAPI-A:DAPI-H ratio (gate A). EdU+, pH3+ and pLaminA/C+ populations were selected by using the gate A population and plotting DAPI-A vs. EdU-Alexa555 , pH3-Alexa488 or pLaminA/C-647 respectively and selecting populations with high expression. The proportion of pH3+ and pLaminA/C+ within the EdU+ population was calculated and reported.

☒ Tick this box to confirm that a figure exemplifying the gating strategy is provided in the Supplementary Information.
